# Tris(pentafluoroethyl)difluorophosphorane: A Versatile Fluoride Acceptor for Transition Metal Chemistry

**DOI:** 10.1002/chem.202004885

**Published:** 2021-01-26

**Authors:** Steffen A. Föhrenbacher, Mirjam J. Krahfuss, Ludwig Zapf, Alexandra Friedrich, Nikolai V. Ignat'ev, Maik Finze, Udo Radius

**Affiliations:** ^1^ Institute of Inorganic Chemistry Julius-Maximilians-Universität Würzburg Am Hubland 97074 Würzburg Germany; ^2^ Institute for Sustainable Chemistry & Catalysis with Boron (ICB) Julius-Maximilians-Universität Würzburg Am Hubland 97074 Würzburg Germany; ^3^ Consultant Merck KGaA Frankfurter Strasse 250 64293 Darmstadt Germany

**Keywords:** copper, nickel, phosphoranes, titanium, weakly coordinating anions

## Abstract

Fluoride abstraction from different types of transition metal fluoride complexes [L_*n*_MF] (M=Ti, Ni, Cu) by the Lewis acid tris(pentafluoroethyl)difluorophosphorane (C_2_F_5_)_3_PF_2_ to yield cationic transition metal complexes with the tris(pentafluoroethyl)trifluorophosphate counterion (**FAP** anion, [(C_2_F_5_)_3_PF_3_]^−^) is reported. (C_2_F_5_)_3_PF_2_ reacted with *trans*‐[Ni(*i*Pr_2_Im)_2_(Ar^F^)F] (*i*Pr_2_Im=1,3‐diisopropylimidazolin‐2‐ylidene; Ar^F^=C_6_F_5_, **1 a**; 4‐CF_3_‐C_6_F_4_, **1 b**; 4‐C_6_F_5_‐C_6_F_4_, **1 c**) through fluoride transfer to form the complex salts *trans*‐[Ni(*i*Pr_2_Im)_2_(solv)(Ar^F^)]**FAP** (**2 a**‐**c[solv]**; solv=Et_2_O, CH_2_Cl_2_, THF) depending on the reaction medium. In the presence of stronger Lewis bases such as carbenes or PPh_3_, solvent coordination was suppressed and the complexes *trans*‐[Ni(*i*Pr_2_Im)_2_(PPh_3_)(C_6_F_5_)]**FAP** (***trans*** **‐2 a[PPh_3_]**) and *cis*‐[Ni(*i*Pr_2_Im)_2_(Dipp_2_Im)(C_6_F_5_)]**FAP** (***cis*** **‐2 a[Dipp_2_Im]**) (Dipp_2_Im=1,3‐bis(2,6‐diisopropylphenyl)imidazolin‐2‐ylidene) were isolated. Fluoride abstraction from [(Dipp_2_Im)CuF] (**3**) in CH_2_Cl_2_ or 1,2‐difluorobenzene led to the isolation of [{(Dipp_2_Im)Cu}_2_]^2+^2 **FAP^−^** (**4**). Subsequent reaction of **4** with PPh_3_ and different carbenes resulted in the complexes [(Dipp_2_Im)Cu(LB)]**FAP** (**5 a**–**e**, LB=Lewis base). In the presence of C_6_Me_6_, fluoride transfer afforded [(Dipp_2_Im)Cu(C_6_Me_6_)]**FAP** (**5 f**), which serves as a source of [(Dipp_2_Im)Cu)]^+^. Fluoride abstraction of [Cp_2_TiF_2_] (**7**) resulted in the formation of dinuclear [FCp_2_Ti(μ‐F)TiCp_2_F]**FAP** (**8**) (Cp=η^5^‐C_5_H_5_) with one terminal fluoride ligand at each titanium atom and an additional bridging fluoride ligand.

## Graphical abstract

**A series of titanium, nickel**, and copper complexes stabilized by the weakly coordinating anion (WCA) [(C_2_F_5_)_3_PF_3_]^−^ (FAP anion) was obtained on reaction of the parent fluoride complexes with the strong Lewis acid (C_2_F_5_)_3_PF_2_. The reaction products depend on the solvent used or additional Lewis bases present during the fluoride transfer from the metal complexes to the phosphorane.

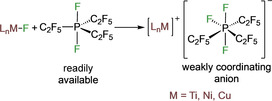

## Introduction

Weakly coordinating anions (WCAs)^[1]^ play an important role in the stabilization of electrophilic cations,^[2]^ as the anionic part of ionic liquids,^[3]^ or in materials applications such as lithium‐ion batteries.^[4]^ A common feature of most WCAs is a central atom surrounded by fluorine atoms and/or perfluorinated organic groups. Among the most easily accessible and cheapest WCAs are the main group element complex fluorides [PF_6_]^−^, [BF_4_]^−^, and [SbF_6_]^−^. These relatively stable anions can be introduced into new compounds either by salt metathesis or by use of the corresponding neutral Lewis acids PF_5_, BF_3_, or SbF_5_ as fluoride acceptor. The latter method is elegant, as the cation is synthesized in the same reaction step as the WCA. For example, the compounds O_2_[BF_4_] and O_2_[PF_6_] can be prepared by fluoride transfer from O_2_F_2_ to BF_3_ or PF_5_ accompanied by the release of 0.5 equiv of elemental fluorine.^[5]^ The complexes [M(CO)_6_]^2+^2 [BF_4_]^−^ (M=Fe, Ru, Os) are accessible through oxidation of [M_3_(CO)_12_] with F_2_ in anhydrous HF (aHF) and subsequent reaction with CO and HF/BF_3_.^[6]^ However, BF_3_ and PF_5_ are gases with low boiling points and are extremely sensitive towards moisture, which makes handling of these substances in the laboratory difficult. Antimony pentafluoride is also an excellent fluoride acceptor. Due to the high fluoride‐ion affinity (FIA)^[7]^ of SbF_5_, for example, the salt [H_3_SO_4_][SbF_6_] was obtained by treatment of (Me_3_SiO)_2_SO_2_ with HF/SbF_5_.^[8]^ The high Lewis acidity of SbF_5_ was also exploited in the first chemical synthesis of elemental fluorine in 1986 by Christe, who used K_2_[MnF_6_] as fluorine source.^[9]^ However, the use of SbF_5_ in the laboratory also has some major drawbacks, as it is a hygroscopic liquid at room temperature that undergoes rapid hydrolysis in contact with water or atmospheric moisture liberating HF. It is a strong oxidizing agent, corrosive to metals, and very hazardous to the eyes, skin, and tissue. SbF_5_ belongs to a group of heavy metal reagents subject to restrictions for practical applications.

More complex WCAs are formed by formal substitution of the fluoro substituents with strongly electron withdrawing (per)fluorinated organic and inorganic groups. The fluorine atoms of [BF_4_]^−^ can be substituted with different groups without losing or even enhancing the properties as a WCA. For example, an “inorganic” replacement would be substitution with teflate groups OTeF_5_. The salt [Tl(mesitylene)_2_][B(OTeF_5_)_4_] was obtained by transfer of the teflate group from Tl(OTeF_5_) to the Lewis acid B(OTeF_5_)_3_ in mesitylene.^[10]^ [B(OTeF_5_)_4_]^−^ is less coordinating than the teflate anion [OTeF_5_]^−^, which is a good metal coordinator, for example, in the nickel complexes [Ni(Hacac)_2_(OTeF_5_)_2_] and [Ni(*i*Pr_2_Im)_2_(OTeF_5_)_2_] (acac=acetylacetonate; *i*Pr_2_Im=1,3‐diisopropylimidazolin‐2‐ylidene).^[11]^

Perfluoroalkyl(fluoro)borates are another class of boron‐based WCAs.[^1^, ^12^] The tetrakis(trifluoromethyl)borate anion [B(CF_3_)_4_]^−[12a, 13]^ was used for the stabilization of reactive cations such as [H(OEt_2_)_2_]^+ [14]^ and N_5_^+^.^[15]^ Mixed perfluoroalkyl(fluoro)borate anions of the general formula [R^F^_*n*_BF_4−*n*_]^−^, for example, [(C_2_F_5_)BF_3_]^−[16]^ and [(C_2_F_5_)_3_BF]^−^,^[17]^ and related anions, [(C_2_F_5_)BF_3−*x*_(CN)_*x*_]^−^,^[18]^ have also been synthesized and studied as WCAs. However, perfluoroalkylboron Lewis acids R^F^_*n*_BF_3−*n*_ (*n*=1–3), which would be ideal starting materials for the aforementioned boron‐based WCAs, are rare.[^12^, ^19^] Trifluoromethyl(fluoro)boranes (CF_3_)_*n*_BF_3−*n*_ (*n*=1–3), bis(perfluoroalkyl)fluoroboranes R^F^_2_BF, and tris(perfluoroalkyl)boranes R^F^_3_B are unstable; only mono(perfluoroalkyl)difluoroboranes C_*x*_F_2*x*+1_BF_2_ (*x*>1) are available.[^12a^, ^19^] It was shown that (CF_3_)_3_BCO is a valuable synthon for the Lewis acid B(CF_3_)_3_, as it can be used as starting material for a variety of WCAs of the form [(CF_3_)_3_BX]^−^ (X=Cl, C(O)F, C(O)NH_2,_ CP, CAs, etc.).[^12a^, ^20^] For example, the reaction of (CF_3_)_3_BCO with Co_2_(CO)_8_ in hexane afforded the Lewis acid–base adduct [Co_2_(CO)_7_COB(CF_3_)_3_],^[21]^ whereas in aHF the ionic compound [Co(CO)_5_][(CF_3_)_3_BF] was formed.^[22]^

**Figure 1:**
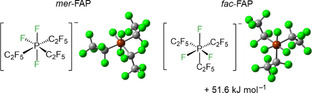
Isomers of the **FAP** anion: *mer*‐**FAP** (left) and *fac*‐**FAP** (right) and their relative energies calculated at the DFT/PBE0/def2‐TZVP‐level of theory.

Perfluoroalkyl(fluoro)phosphate anions are another important class of WCAs. In particular, the tris(pentafluoroethyl)trifluorophosphate anion (**FAP** anion, [(C_2_F_5_)_3_PF_3_]^−^) has been extensively studied in recent years. The **FAP** anion, which has been commercialized, is readily accessible from the Lewis acid tris(pentafluoroethyl)difluorophosphorane (C_2_F_5_)_3_PF_2_ and a fluoride source. The two isomers of the **FAP** anion, *mer*‐**FAP** and *fac*‐**FAP**, differ by 51.6 kJ mol^−1^ in energy (see Figure 1). Typically, the thermodynamically favorable isomer *mer*‐**FAP** is by far the dominant species in the reaction mixtures, and the less stable *fac*‐**FAP** is observed as minor component only by ^19^F and ^31^P NMR spectroscopy. The **FAP** anion is less coordinating than the parent hexafluorophosphate anion [PF_6_]^−^ and it is more stable with respect to hydrolysis and decomposition.^[23]^ Thus, salts such as Li**FAP** were studied as a substitute for Li[PF_6_] as the electrolyte in Li‐ion batteries.^[24]^ Furthermore, ionic liquids based on the **FAP** anion have been developed and their properties have been determined.^[25]^

Tris(pentafluoroethyl)difluorophosphorane (C_2_F_5_)_3_PF_2_ is accessible by electrochemical fluorination of Et_3_P in aHF^[26]^ according to the Simons process on an industrial scale.^[27]^ (C_2_F_5_)_3_PF_2_ is a liquid with b.p. 91–92 °C.^[23]^ It is only slowly hydrolyzed on contact with ice‐water, liberating HF. The phosphorane (C_2_F_5_)_3_PF_2_ is known to react as a strong Lewis acid with various nucleophiles, such as F^−^,[^23^, ^26b^] Cl^−^,^[28]^ H^−^,^[29]^ HO^−^, and CH_3_C(O)O^−^.^[30]^ Its FIA has been reported to be 389.3 kJ mol^−1^.[^23^, ^26b^] We re‐evaluated the FIA according to the procedure presented by Krossing et al.^[31]^ (see Supporting Information) to be 405.4 kJ mol^−1^. Thus, (C_2_F_5_)_3_PF_2_ is more Lewis acidic than PF_5_ (395.0 kJ mol^−1^) and in the range of AsF_5_ (FIA=427.6 kJ mol^−1^), but still a weaker Lewis acid than SbF_5_ (FIA=476.1 kJ mol^−1^). (C_2_F_5_)_3_PF_2_ can abstract fluoride from PF_6_ salts with formation of **FAP** salts and liberation of PF_5_.[^26b^, ^32^] Due to its high Lewis acidity the phosphorane (C_2_F_5_)_3_PF_2_ has been used as a catalyst, for example, in Diels–Alder reactions^[33]^ and Michael additions.^[34]^ It was shown that (C_2_F_5_)_3_PF_2_ enhances the Brønsted acidity of acetonitrile, which, in the presence of Et_3_N, enabled the synthesis of [Et_3_NH][(C_2_F_5_)_3_PF_2_(CH_2_CN)] with deprotonated acetonitrile CH_2_CN^−^ coordinated to the phosphorus center.^[35]^ As noted above, the **FAP** anion [(C_2_F_5_)_3_PF_3_]^−^ can be obtained from (C_2_F_5_)_3_PF_2_ and a suitable fluoride source. Thus, **FAP** salts have been obtained by fluoride abstraction from main group element fluorides. For example, Ignat'ev, Hoge, and co‐workers recently reported on fluoride transfer from Ph_3_PF_2_ and Ph_3_BiF_2_ to (C_2_F_5_)_3_PF_2_ providing [Ph_3_PF]**FAP** and [Ph_3_BiF]**FAP**,^[36]^ respectively, and on the generation of neutral (C_2_F_5_)_3_GeF by halide abstraction from [(C_2_F_5_)_3_GeF_2_]^−^ with (C_2_F_5_)_3_PF_2_.^[37]^

A few cationic transition metal complexes with the **FAP** anion are known. All of them have been synthesized by anion metathesis. Wasserscheid et al. obtained a series of silver salts by metathesis using AgNO_3_ and K**FAP** in different solvents and investigated propene/propane separation using these salts.^[38]^ The complexes [Co(η^5^‐C_5_H_5_)_2_]**FAP** and [Ru(η^5^‐C_5_H_5_)(η^6^‐C_6_H_6_)]**FAP** have been prepared by anion exchange of the parent metal chloride complexes with Na**FAP**.^[39]^

**Scheme 1:**
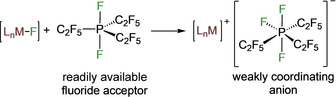
Convenient synthesis of cationic transition metal complexes stabilized by the **FAP** anion through fluoride transfer.

Surprisingly, fluoride abstraction from transition metal fluoride complexes by tris(pentafluoroethyl)difluorophosphorane is unexplored. So, we initiated a systematic investigation of the suitability of (C_2_F_5_)_3_PF_2_ as a fluoride acceptor in transition metal chemistry (see Scheme 1). Various 3d metal fluoride complexes of the transition metals nickel, copper, and titanium were studied, as these earth‐abundant metals^[40]^ are becoming more and more relevant for catalytic applications.^[41]^ Metal fluoride complexes of the 3d metals are often readily accessible.^[42]^

## Results and Discussion

### Nickel complexes

**Scheme 2:**
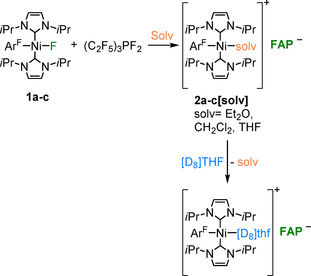
Synthesis of [Ni(*i*Pr_2_Im)_2_(solv)(Ar^F^)]**FAP** (**2 a**‐**c[solv]**).

Some of us reported previously on nickel‐mediated C−F bond activation in stoichiometric and catalytic reactions.[^42b^, ^42c^, ^43^] Complexes of the type [Ni(NHC)_2_] (NHC=*N*‐heterocyclic carbene) react with aryl fluorides Ar^F^F with insertion into the C−F bonds to yield *trans*‐[Ni(NHC)_2_(Ar^F^)F] complexes.[^42b^, ^42c^] Thus, we started our investigation on tris(pentafluoroethyl)difluorophosphorane (C_2_F_5_)_3_PF_2_ as fluoride acceptor with *trans*‐[Ni(*i*Pr_2_Im)_2_(Ar^F^)F] (Ar^F^=C_6_F_5_, **1 a**; 4‐CF_3_‐C_6_F_4_, **1 b**; 4‐C_6_F_5_‐C_6_F_4_, **1 c**). We found that the choice of the solvent is crucial for these reactions, because 1) the nickel complex/phosphorane mixture polymerized solvents such as THF, 2) halogenated solvents such as trichloromethane led to halide exchange at nickel, and 3) other solvents, especially benzene and toluene, led to solubility issues (the phosphorane is not soluble in these solvents). Finally, diethyl ether was found to be the solvent of choice. The characteristic NiF resonance in the ^19^F NMR spectrum of **1 a**–**c** in the region between −370 and −375 ppm vanished on reaction with (C_2_F_5_)_3_PF_2_ in Et_2_O. After addition of the phosphorane to a suspension of complexes **1 a**–**c** in Et_2_O, clear yellow solutions were obtained immediately, which led to the isolation of *trans*‐[Ni(*i*Pr_2_Im)_2_(OEt_2_)(Ar^F^)]**FAP** (Ar^F^=C_6_F_5_, **2 a[OEt_2_]**; 4‐CF_3_‐C_6_F_4_, **2 b[OEt_2_]**; 4‐C_6_F_5_‐C_6_F_4_, **2 c[OEt_2_]**) as yellow powders (see Scheme 2).

**Figure 2:**
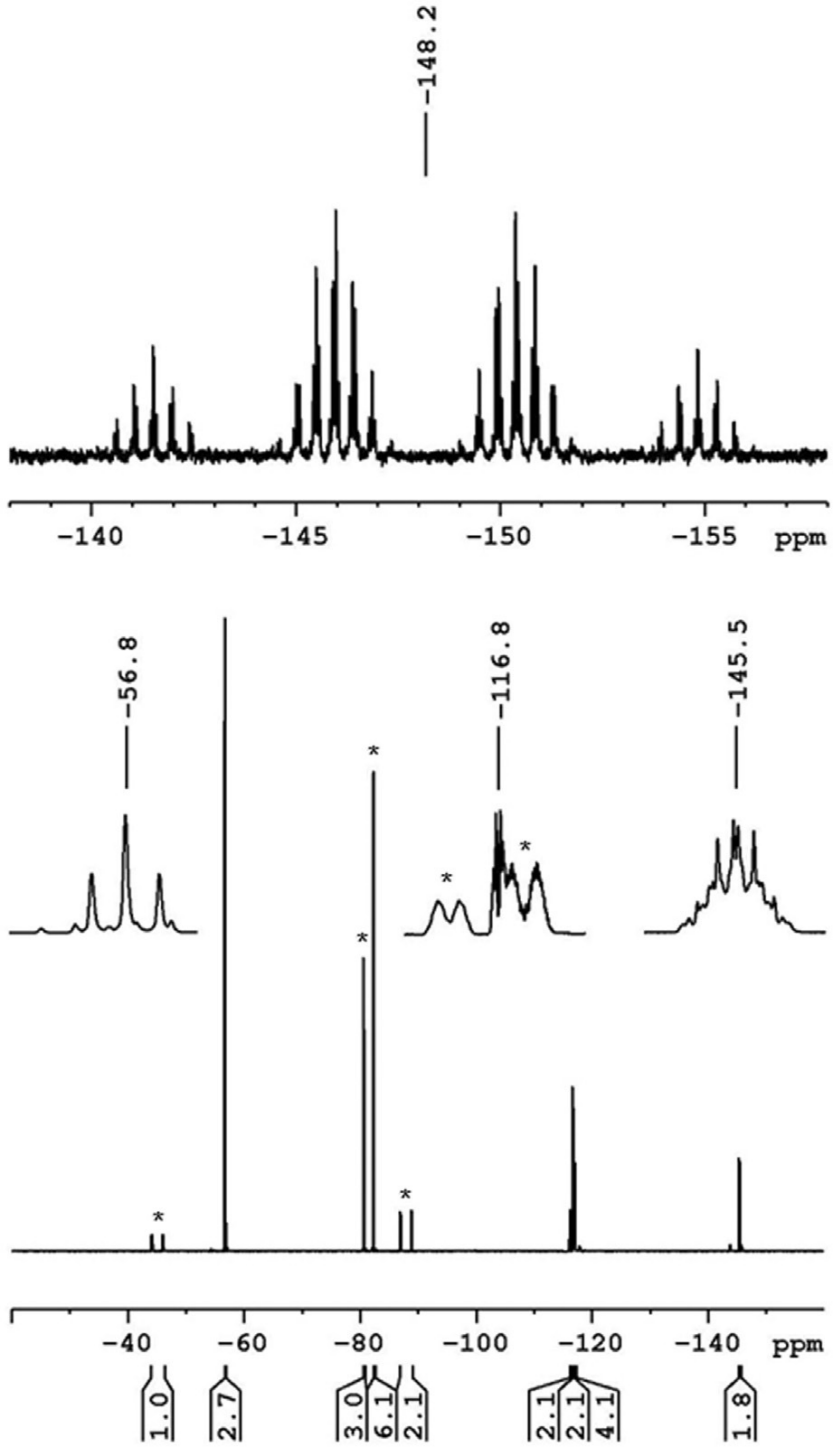
^31^P (top) and ^19^F NMR spectra (bottom) of **2 b[OEt_2_]**; the ^19^F NMR signals of the **FAP** anion are marked with asterisks.

The abstraction of fluoride from the nickel complexes and the formation of the **FAP** anion was evident from both ^19^F and ^31^P NMR spectra. The reaction of the nickel fluorides with (C_2_F_5_)_3_PF_2_ afforded the more stable *mer*‐**FAP** ion of lower symmetry (with respect to the *fac*‐isomer, see Figure 1), according to ^31^P and ^19^F NMR spectroscopy. In the ^31^P NMR spectra of the isolated products in [D_8_]THF solution, the characteristic signal of the FAP anion was detected at −148.2 ppm (see Figure 2, top). The chemical shift and the multiplicity (tdm) with ^1^ *J*_P–F_ coupling constants of 905 and 891 Hz were in accordance with those reported for the **FAP** anion previously.[^32a^, ^36^] Furthermore, the **FAP** anion gave rise to resonances at −45.1 (P*F*), −80.7 (C*F*_3_), −82.4 (C*F*_3_), −88.0 (P*F*_2_), −116.4 (C*F*_2_), and −117.0 ppm (C*F*_2_) with relative intensities of 1:3:6:2:2:4 in the ^19^F NMR spectra of the complexes (see Figure 2, bottom). The NiF resonances were no longer detected in the ^19^F NMR spectra which indicated complete fluoride transfer from nickel to phosphorus. The signals of the fluoroaryl ligands were almost unaffected (see Table S1 in the Supporting Information). The ether ligand in *trans*‐[Ni(*i*Pr_2_Im)_2_(OEt_2_)(Ar^F^)]**FAP** is only loosely bound to the nickel atom. In the ^1^H NMR spectrum of **2 a[OEt_2_]** in [D_8_]THF, a triplet at 1.12 ppm and a quartet at 3.39 ppm were observed for uncoordinated Et_2_O,^[44]^ that is, in [D_8_]THF ligand exchange occurred and the Et_2_O ligand was replaced with [D_8_]THF. The NHC ligands gave rise to two sharp doublets at 1.36 and 1.57 ppm for the *i*Pr methyl groups, a septet at 6.18 ppm (^3^ *J*_H‐H_=6.7 Hz) for the methine protons, and a singlet at 7.40 ppm for the olefinic protons of the backbone. The integration of the signals of the *i*Pr methyl groups and the methyl groups of Et_2_O resulted in a 1:1 molar ratio of complex:Et_2_O. After removal of all volatile substances in vacuo and redissolution in [D_8_]THF, the signals at 1.12 and 3.39 ppm were no longer detected. Ether exchange was also observed for *trans*‐[Ni(*i*Pr_2_Im)_2_(OEt_2_)(4‐CF_3_‐C_6_F_4_)]**FAP** (**2 b[OEt_2_]**) and *trans*‐[Ni(*i*Pr_2_Im)_2_(OEt_2_)(4‐C_6_F_5_‐C_6_F_4_)]**FAP** (**2 c[OEt_2_]**), which does not affect the signals of the **FAP** anion.

In the ^13^C{^1^H} MAS NMR spectrum of **2 a[OEt_2_]** two sets of signals were observed for the coordinated Et_2_O (see Figure S5 of the Supporting Information). In the solid‐state ^31^P{^19^F} MAS NMR spectrum two signals for the counterion were observed as well (see Figure S6 of the Supporting Information). Both observations are indicative of two independent positions of the complex salt in the solid state. The formation of cationic metal complexes was also evident from high‐resolution mass spectra of complexes **2 a**–**c[OEt_2_]**. In each spectrum, a signal at *m*/*z* 444.94 was detected in the negative‐ion mode for the **FAP** anion and a signal for the [Ni(*i*Pr_2_Im)_2_(Ar^F^)]^+^ cation (**2 a**: *m*/*z* 529.19; **2 b**: *m*/*z* 579.19; **2 c**: *m*/*z* 677.18) in the positive‐ion mode.

**Figure 3:**
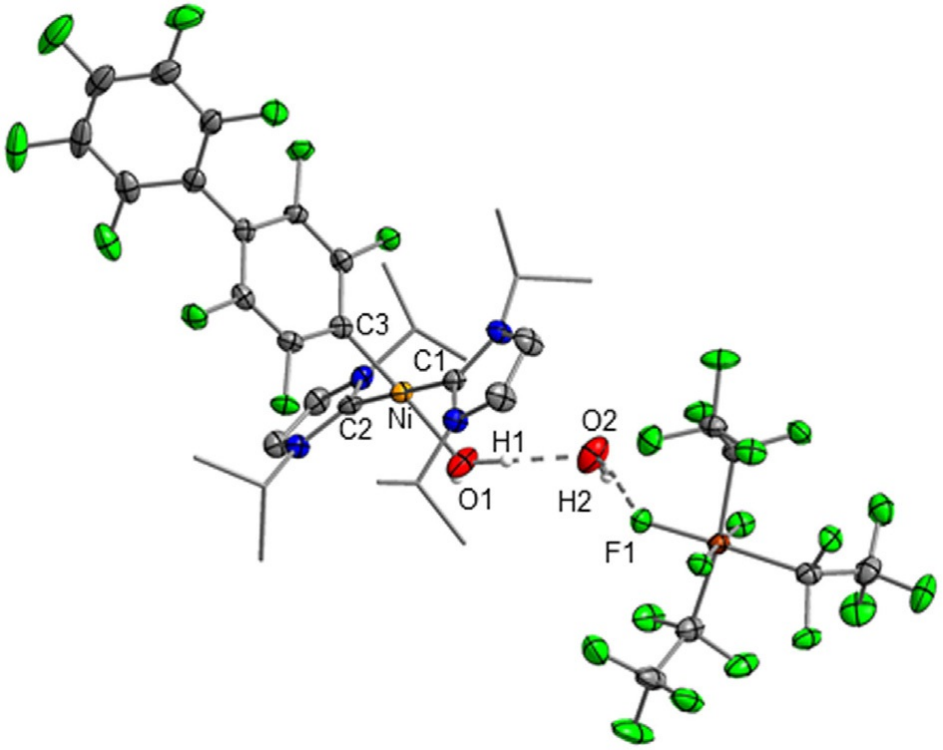
Molecular structure of ***trans*** **‐2 c[OH_2_]⋅H_2_O** in the solid state (ellipsoids set at the 50 % probability level; *i*Pr groups are shown as wire‐and‐stick models). Hydrogen atoms (except those of the H_2_O moieties) are omitted for clarity. Bond lengths [Å] and angles [°]: C1−Ni 1.920(2), C2−Ni 1.931(2), C3−Ni 1.8851(18), O1−Ni 1.9425(16), H1⋅⋅⋅O2 1.74(4), H2⋅⋅⋅F1 2.04(4); C1‐Ni‐C2 179.01(8), C1‐Ni‐C3 89.96(8), C1‐Ni‐O1 89.55(8), C2‐Ni‐C3 90.97(8), C2‐Ni‐O1 89.53(8), C3‐Ni‐O1 177.00(8).

We were not successful in growing high‐quality crystals of **2 a**–**c[OEt_2_]**, although a variety of solvent mixtures was tested. However, addition of pentane to a solution of **2 c[OEt_2_]** in wet toluene led to formation of crystals of the hydrolysis product *trans*‐[Ni(*i*Pr_2_Im)_2_(OH_2_)(4‐C_6_F_5_‐C_6_F_4_)]**FAP⋅**H_2_O (***trans*** **‐2 c[OH_2_]⋅H_2_O**) suitable for X‐ray diffraction (see Figure 3). The complex ***trans*** **‐2 c[OH_2_]⋅H_2_O** crystallizes in the triclinic space group $P\bar{1}$ with one molecule in the asymmetric unit. The molecular structure confirms the ionic nature of [Ni(*i*Pr_2_Im)_2_(OH_2_)(4‐C_6_F_5_‐C_6_F_4_)]**FAP⋅**H_2_O. The nickel atom is almost ideally square‐planar coordinated with the carbene ligands in *trans* positions. The distances of the carbenic carbon atoms C1 and C2 to the nickel atom are identical within 3 *σ* and the fluoride transfer has no influence on the C_NHC_−Ni bond lengths (cf. 1.924(2), 1.933(2) Å for **1 a** ^[43a]^ and 1.932(8), 1.911(8) Å for **1 b** ^[42c]^). The structure reveals the presence of the *mer*‐isomer of the **FAP** anion in agreement with the ^19^F NMR spectrum in solution, which shows only the signals of this isomer. The solid‐state structures of the metal complexes presented previously by Mochida and Kimata^[39]^ and Wasserscheid et al.^[38]^ as well as the fluorophosphonium salt reported by Hoge et al.^[36]^ also contain the *mer*‐isomer of the **FAP** anion.

The water molecule coordinated to the nickel center and a second water molecule bridging the cation and the anion form a hydrogen‐bond motif and interconnect the cation and the anion (see Figure 3). The O1−Ni distance of 1.9425(16) Å is slightly longer than the O−Ni distance observed in [Ni{MesIm(C_2_OMe)}_2_(H_2_O)_2_]^2+^2 [PF_6_]^−^ (1.907(4) Å) (MesIm(C_2_OMe)=1‐(2,4,6‐trimethylphenyl)‐3‐(2‐methoxyethyl)imidazolin‐2‐ylidene).^[45]^ The H1−O2 distance of 1.74(4) Å is in the range expected for hydrogen bonds formed by water (1.72–2.19 Å).^[46]^ The H2−F1 distance of 2.03(4) Å corresponds to a rather short fluorine hydrogen bond, which might be due to the fact that the fluorine atom involved is part of an anion and thus electron rich. This requirement for short fluorine hydrogen bonds was already pointed out previously by Dunitz and Taylor.^[47]^

To gain further insight into the coordination of the solvent, the fluoride abstraction of **1 a** was performed in Et_2_O/THF (1:1). Interestingly, the mixture of these two solvents and the phosphorane did not lead to polymerization of THF. After workup, *trans*‐[Ni(*i*Pr_2_Im)_2_(thf)(C_6_F_5_)]**FAP** (**2 a[thf]**) was obtained as a yellow powder with THF coordinated to nickel. The ^1^H NMR spectrum of this powder in CD_2_Cl_2_ shows, besides the signals of the NHC ligands, two resonances for the THF ligand at 1.83 and 3.20 ppm, which are only slightly shifted relative to noncoordinated THF in CD_2_Cl_2_ (1.81 and 3.67 ppm) but indicative of THF coordination. Similarly, the resonances of the coordinated THF (25.7 and 74.1 ppm) are shifted compared to uncoordinated THF (26.0 and 68.2 ppm) in the ^13^C{^1^H} NMR spectrum.

As Et_2_O and THF are rather strongly coordinating solvents, we examined the fluoride transfer from **1 a** to (C_2_F_5_)_3_PF_2_ in CH_2_Cl_2_ and 1,2‐difluorobenzene, which are both weaker donor solvents. The reaction of **1 a** with the phosphorane in 1,2‐difluorobenzene led to partial decomposition of the nickel complex, whereas CH_2_Cl_2_ proved to be a suitable solvent for this reaction. Although the phosphorane is not soluble in CH_2_Cl_2_, clear solutions were obtained after addition of the phosphorane to solutions of **1 a**–**c** in CH_2_Cl_2_. Removal of all volatile substances and drying in vacuo led to isolation of the CH_2_Cl_2_‐ coordinated complexes *trans*‐[Ni(*i*Pr_2_Im)_2_(ClCH_2_Cl)(C_6_F_5_)]**FAP** (**2 a[ClCH_2_Cl]**) and *trans*‐[Ni(*i*Pr_2_Im)_2_(ClCH_2_Cl)(4‐C_6_F_5_‐C_6_F_4_)]**FAP** (**2 c[ClCH_2_Cl]**). The presence of one equivalent of CH_2_Cl_2_ was confirmed by ^1^H NMR spectroscopy of the compounds in [D_8_]THF. The reaction of (C_2_F_5_)_3_PF_2_ with **1 b** in CH_2_Cl_2_ resulted in an impure, sticky, oily residue after removal of all volatile substances in vacuo. To obtain further evidence for solvent coordination, differential scanning calorimetry (DSC) of complexes **2 a[OEt_2_]**, **2 a[ClCH_2_Cl]**, and **2 a[thf]** was carried out (see Figure S66 of the Supporting Information). The DSC curves revealed endothermic processes at onset temperatures of 50 (**2 a[ClCH_2_Cl]**), 73 (**2 a[OEt_2_]**) and 125 °C (**2 a[thf]**), which correspond to solvent removal and lie significantly higher than the boiling points of the corresponding solvents. According to the onset temperatures and the differences compared with the boiling points of the solvents, it can be concluded that the M–solvent binding energy increases in the order CH_2_Cl_2_<Et_2_O<THF, which is in agreement with the exchange of diethyl ether and CH_2_Cl_2_ with [D_8_]THF observed. Furthermore, the complexes **2 a[ClCH_2_Cl]** and **2 a[OEt_2_]** decompose slowly at room temperature in the solid state and in solution, whereas **2 a[thf]** is stable under ambient conditions. However, all complexes including **2 a[ClCH_2_Cl]** and **2 a[OEt_2_]** can be stored in the solid state at −80 °C for weeks without any decomposition.

**Scheme 3:**
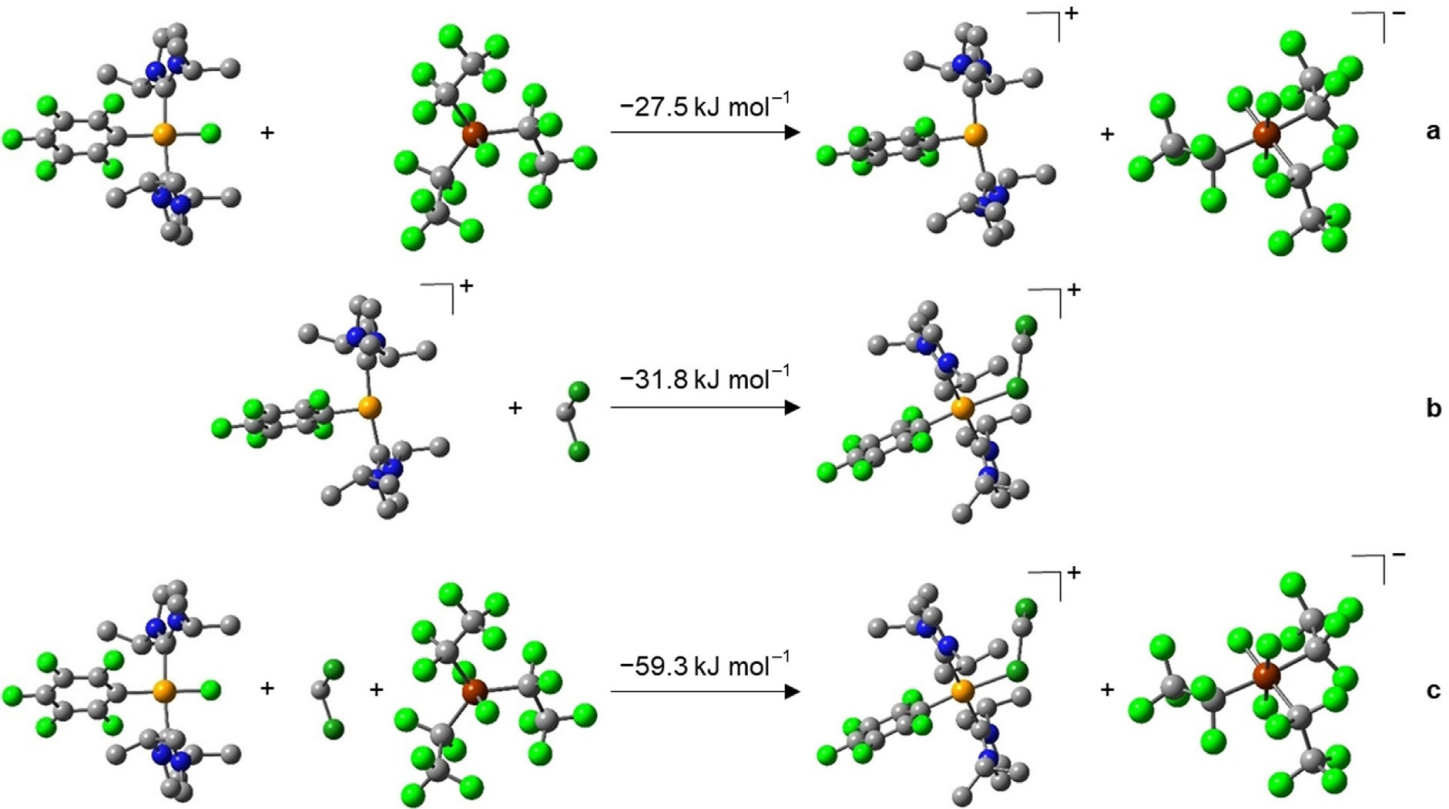
Optimized geometries (hydrogen atoms are omitted for clarity) and calculated reaction energies (DFT, PBE0/def2‐TZVP/COSMO) of the fluoride transfer from **1 a** to (C_2_F_5_)_3_PF_2_ and subsequent formation of **2 a[ClCH_2_Cl]**.

Quantum chemical calculations (DFT, PBE0/def2‐TZVP; for details, see the Supporting Information) were performed on the fluoride transfer from *trans*‐[Ni(*i*Pr_2_Im)_2_(C_6_F_5_)F] **1 a** to (C_2_F_5_)_3_PF_2_ (see Scheme 3). Fluoride transfer from the nickel complex to the phosphorane to yield the three‐coordinate nickel cation and the **FAP** anion is exothermic (−27.5 kJ mol^−1^) and should thus occur even in the absence of an additional donor solvent that binds to the nickel cation (see Scheme 3 a). However, coordination even of the weak donor CH_2_Cl_2_ contributes significantly to the stabilization of the cationic nickel complex (−31.8 kJ mol^−1^, see Scheme 3 b) and thus to the exothermicity (−59.3 kJ mol^−1^, see Scheme 3 c) of the formation of **2 a[ClCH_2_Cl]**.

**Scheme 4:**
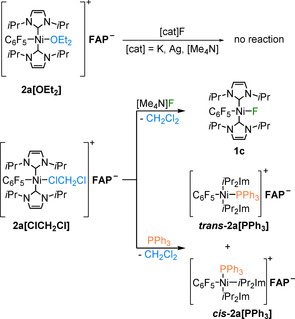
Reactivity of **2 a[solv]** towards two‐electron donors.

For further examination of the reactivity of the complex cations **2 a**‐**c[OEt_2_]** with two‐electron donor ligands we first tried to re‐establish the Ni−F bond using different fluorinating agents (see Scheme 4). All attempts to refluorinate **2 a[OEt_2_]** with AgF, [Me_4_N]F, KF, and [C_7_H_14_ClFN_2_]^2+^2 [BF_4_]^−^ (Selectfluor) failed according to ^19^F NMR spectroscopy. However, as solvent‐coordinated complexes were used as starting materials, we finally succeeded by using CH_2_Cl_2_ as a less coordinating solvent compared to Et_2_O. In contrast to **2 a[OEt_2_]**, the complex **2 a[ClCH_2_Cl]** was refluorinated by using [Me_4_N]F as fluoride source to yield **1 a**, which was confirmed by ^19^F NMR spectroscopy (Scheme 4; Figure S23 of the Supporting Information).

**Figure 4:**
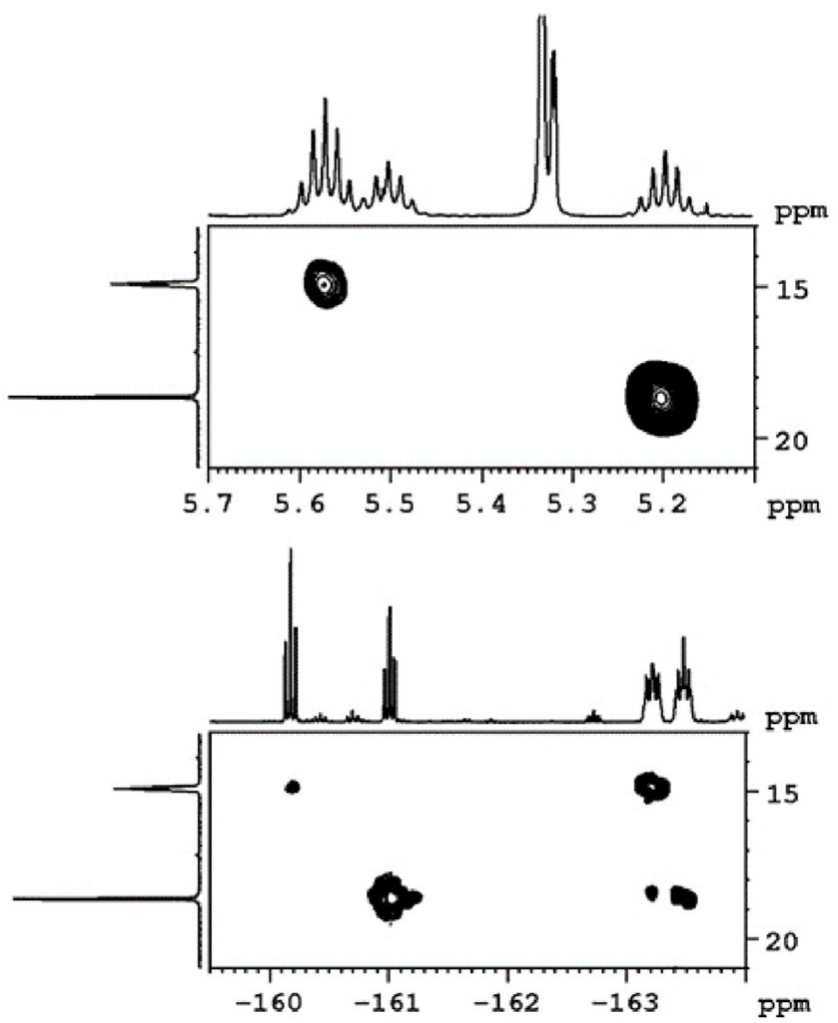
^31^P,^1^H HMQC (top), and ^31^P,^19^F HMQC spectra (bottom) of the reaction mixture of **2 a[ClCH_2_Cl]** with PPh_3_ in CD_2_Cl_2_.

As the nickel cation [Ni(*i*Pr_2_Im)_2_(C_6_F_5_)]^+^ is stabilized by the solvent, we also tried to substitute the solvent by other Lewis bases. For example, treating **2 a[ClCH_2_Cl]** with PPh_3_ in CD_2_Cl_2_ led to two additional resonances in the ^31^P NMR spectrum at 14.9 and 18.6 ppm (cf. noncoordinated PPh_3_ at −5.4 ppm), indicative of formation of a 1:1 mixture of ***cis***‐**2 a[PPh_3_]** and ***trans***‐**2 a[PPh_3_]**. The formation of these stereoisomers was confirmed by ^1^H and ^19^F NMR spectra of the reaction mixture. Three septets were observed for the methine protons of the *i*Pr groups at 5.20, 5.50, and 5.57 ppm with relative intensities of 2:2:4. For the *cis* isomer, the resonances of the NHC ligands were split into two septets at 5.20 and 5.50 ppm. Therefore, the septet at 5.57 ppm was assigned to the *trans* isomer. Furthermore, in the ^31^P,^1^H HMQC spectrum, the methine protons at 5.20 ppm revealed coupling to the phosphorus signal at 18.6 ppm, and the septet at 5.57 ppm with the phosphorus resonance at 14.9 ppm (see Figure 4, top). In the ^19^F NMR spectrum, two sets of resonances for the fluoro substituents of the aryl ligand were observed. In the ^31^P,^19^F HMQC spectrum (see Figure 4, bottom), coupling between the phosphorus nucleus with *δ*=14.9 ppm and the fluorine atoms with resonances at −115.3, −160.2, and −163.2 ppm was observed. The fluoro substituents assigned to the signals at −116.4, −161.0, and −163.5 ppm showed coupling to the signal of the phosphorus atom of the isomer at 18.6 ppm.

**Figure 5:**
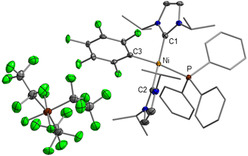
Molecular structure of ***trans*** **‐2 a[PPh_3_]** in the solid state (ellipsoids set at the 50 % probability level; *i*Pr and phenyl groups are shown as wire‐and‐stick models). Hydrogen atoms are omitted for clarity. Bond lengths [Å] and angles [°]: C1−Ni 1.905(4), C2−Ni 1.936(4), C3−Ni 1.942(4), P−Ni 2.2588(12); C1‐Ni‐C2 166.15(16), C1‐Ni‐C3 88.68(16), C1‐Ni‐P 93.33(12), C2‐Ni‐C3 88.25(16), C2‐Ni‐P 90.52(12), C3‐Ni‐P 167.5(12).

After addition of pentane to a solution of a 1:1 mixture of both stereoisomers in CH_2_Cl_2_, yellow blocks of ***trans*** **‐2 a[PPh_3_]** suitable for X‐ray diffraction formed overnight (see Figure 5). The complex ***trans*** **‐2 a[PPh_3_]** crystallizes in the triclinic space group $P\bar{1}$ with one molecule in the asymmetric unit. The nickel atom is coordinated to two NHC ligands, the phosphine ligand, and the fluoroaryl group and thus adopts a distorted square‐planar geometry. The PPh_3_ ligand is *trans* to the perfluoroaryl ligand. The Ni−P distance of 2.2588(12) Å is close to the average Ni−P distance in [Ni(PPh_3_)_3_(SO_2_)] (2.260(4) Å)^[48]^ and slightly longer than *d*(Ni−P) observed for the cationic unit in *trans*‐[Ni((MeO)_2_Im)_2_(PPh_3_)Br][PF_6_] (2.1811(8) Å) [(MeO)_2_Im=1,3‐dimethoxyimidazolin‐2‐ylidene],^[49]^ or for neutral [Ni(PPh_3_)_3_(N≡CPh)] (average Ni−P distance: 2.190(5) Å).^[50]^ The C2−Ni (1.936(4) Å) and C3−Ni (1.942(4) Å) distances are identical within 3 *σ* and slightly longer than *d*(C1−Ni) (1.905(4) Å).

**Figure 6:**
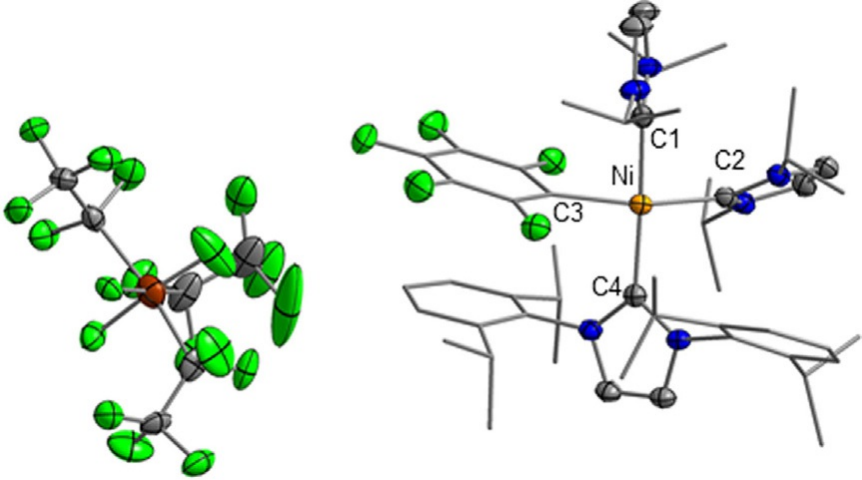
Molecular structure of ***cis*** **‐2 a[Dipp_2_Im]** in the solid state (ellipsoids set at the 50 % probability level; *i*Pr and phenyl groups are shown as wire‐and‐stick models). Hydrogen atoms are omitted for clarity. Bond lengths [Å] and angles [°]: C1−Ni 1.9545(18), C2−Ni 1.9709(18), C3−Ni 1.9548(18), C4−Ni 1.9904(17), C1‐Ni‐C2 89.35(7), C1‐Ni‐C3 82.91(7), C1‐Ni‐C4 173.88(7), C2‐Ni‐C3 163.00(7), C2‐Ni‐C4 94.92(7), C3‐Ni‐C4 94.09(7).

The fluoride abstraction from **1 a** with (C_2_F_5_)_3_PF_2_ in the presence of PPh_3_ also resulted in the formation of a ***cis*** **/*trans*‐2 a[PPh_3_]** mixture according to ^31^P NMR spectroscopy [*δ*(^31^P): 18.6, 14.9 ppm] and HRMS ([Ni(*i*Pr_2_Im)_2_(PPh_3_)(C_6_F_5_)]^+^: *m*/*z* 791.28; ESI+ MS). However, the crude material from this reaction typically contains some unknown impurities, and all attempts to purify the product further have failed, so far. Fluoride abstraction from **1 a** in the presence of NHCs was also studied, but these reactions were unspecific and typically gave mixtures of products, which were not separated. For example, the reaction of **1 a** with (C_2_F_5_)_3_PF_2_ and Dipp_2_Im in Et_2_O afforded, after removal of all volatile substances in vacuo, a yellow powder containing a mixture of products according to ^1^H NMR spectroscopy (the resonances were not assigned completely). However, one of the reaction products is most probably the imidazolium salt [Dipp_2_ImH]**FAP**, as the characteristic signals of the olefinic protons were observed as a doublet at 8.22 ppm, and the imidazolium proton was detected as triplet at 9.59 ppm. This is unexpected, because no protic solvent was used during the reaction and NMR characterization. Furthermore, we isolated a small number of crystals suitable for X‐ray diffraction by vapor‐diffusion of hexane into a solution of the reaction products in 2‐propanol. The crystal structure (see Figure 6) revealed the formation of *cis*‐[Ni(*i*Pr_2_Im)_2_(Dipp_2_Im)(C_6_F_5_)]**FAP** (***cis*** **‐2 a[Dipp_2_Im]**).

The complex ***cis*** **‐2 a[Dipp_2_Im]** crystallizes in the triclinic space group $P\bar{1}$ with one molecule in the asymmetric unit. The environment around the nickel atom is distorted square‐planar, with the two *cis*‐*i*Pr_2_Im ligands. The C1‐Ni‐C2 angle is 89.35(7)° and the C−Ni distances of both the carbenic carbon atoms and the fluoroaryl ligand are similar (1.9545(18)–1.9904(17) Å). The angle between Dipp_2_Im and the *trans*‐*i*Pr_2_Im ligand is 173.88(7)°. The molecule is further stabilized by π‐stacking[^41f^, ^41h^, ^51^] between the C_6_F_5_ ligand and one of the Dipp_2_Im phenyl substituents, as the angle between the best planes through the fluoroaryl ligand and the Dipp_2_Im phenyl substituents is 8.67(9) ° with a distance between the centroids of these two aromatic rings of 3.2402 Å (cf. 3.35 Å in graphite).^[52]^

### Copper complexes

Copper complexes are of increasing importance as catalysts, which tempted us to examine the reactivity of the copper fluoride complex [(Dipp_2_Im)CuF] (**3**) towards the phosphorane (C_2_F_5_)_3_PF_2_. Complex **3** and the parent chloride derivative [(Dipp_2_Im)CuCl] were intensively studied as catalysts for organic transformations.^[53]^

**Scheme 5:**
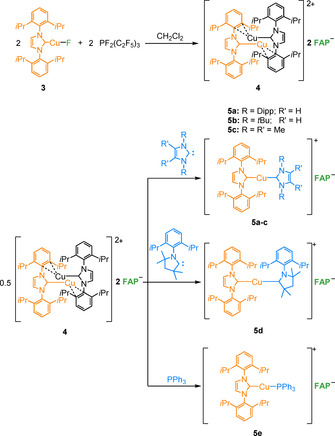
Fluoride transfer from [(Dipp_2_Im)CuF] (**3**) to (C_2_F_5_)_3_PF_2_ and follow‐up reactions of [{(Dipp_2_Im)Cu}_2_]^2+^2 **FAP^−^** (**4**).

**Figure 7:**
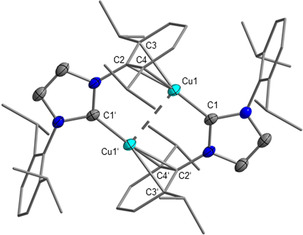
Molecular structure of the dication of [{(Dipp_2_Im)Cu}_2_]^2+^2 **FAP^−^** (**4**) in the solid state (ellipsoids set at the 50 % probability level; *i*Pr groups and phenyl groups are shown as wire‐and‐stick models). Hydrogen atoms are omitted for clarity. Bond lengths [Å] and angles [°]: C1−Cu1 1.890(4), C2−Cu1 2.156(4), C3−Cu1 2.312(5), C4−Cu1 2.355(5), Cu1−Cu1′ 2.8014(13); C1‐Cu1‐C2 170.22(19).

On reaction of **3** with (C_2_F_5_)_3_PF_2_ in CH_2_Cl_2_ or 1,2‐difluorobenzene, the dinuclear complex [{(Dipp_2_Im)Cu}_2_]^2+^2 **FAP** ^−^ (**4**) was obtained as a colorless solid (see Scheme 5). Crystals of **4** suitable for X‐ray diffraction were obtained from a saturated solution of **4** in CH_2_Cl_2_ (see Figure 7). Compound **4** crystallizes in the monoclinic space group *P*2_1_/*c* in the solid state and has half of the centrosymmetric dicationic dinuclear fragment [{(Dipp_2_Im)Cu}_2_]^2+^, one disordered *mer*‐isomer **FAP** counterion, and one disordered CH_2_Cl_2_ solvent molecule in the asymmetric unit (see Figure S67 of the Supporting Information). An inversion center is located between the copper atoms. Besides the coordination of the carbene ligands, the copper atom of the [(Dipp_2_Im)Cu]^+^ unit coordinates to a NHC Dipp phenyl group of the adjacent [(Dipp_2_Im)Cu]^+^ moiety in an η^3^ mode to the *ipso* and *ortho* carbon atoms. Two of these units form the dimeric structure of the dication of **4**. Such η^*n*^ coordination of the metal atom to aryl substituents at the NHC imidazoline nitrogen atoms is rather common, as observed, for example, for the low‐valent, dinuclear NHC complexes [{(Ar_2_Im)M}_2_] (Ar=Mes, M=Fe; Ar=Dipp, M=Co, Ni)^[54]^ and [{(Dipp_2_Im)IrH}_2_]^2+^2 [BF_4_]^−^,^[55]^ in which each NHC ligand is end‐on coordinated to the metal center and one of the aryl substituents is attached through the π system to the adjacent metal atom. The Cu−C distance to the carbenic carbon atom of 1.890(4) Å in compound **4** is slightly longer than the distances observed in the parent fluoride complex [(Dipp_2_Im)CuF] (1.857(3) Å)^[56]^ and the *tert*‐butoxide complex [(Dipp_2_Im)Cu(O*t*Bu)] (1.8641(18) Å)^[57]^ but shorter than those in the related chloride complex [(Dipp_2_Im)CuCl] (1.953(8) Å)^[53c]^ or in the bis‐NHC complexes [(Dipp_2_Im)_2_Cu][PF_6_] (1.938(5) Å) and [(Dipp_2_Im)_2_Cu][BF_4_] (1.939(18) Å).^[58]^ The Cu−C distance to the *ipso* carbon atom C2 (2.156(4) Å) is significantly shorter than the distances to the carbon atoms in *ortho* positions C3 and C4 (2.312(5) and 2.355(5) Å, respectively). Similar η^3^ coordination was observed by Khan and co‐workers, who realized halide abstraction from [(Dipp_2_Im)CuBr] with Ag[SbF_6_] in the presence of hexamethylbenzene to give [(Dipp_2_Im)Cu(C_6_Me_6_)][SbF_6_].^[59]^ The distances between the copper atom and both the carbenic carbon atom and the *ipso*/*ortho* carbon atoms of hexamethylbenzene in this complex are very similar to those found in **4**.^[59]^ Additionally, there is a “cuprophilic” interaction^[60]^ with a Cu**⋅⋅⋅**Cu distance of 2.8014(13) Å, which is slightly longer than those in similar NHC‐stabilized dinuclear dicationic complexes [{(^R^NHCP^*t*Bu^)Cu}_2_]^2+^2 [PF_6_]^−^ (^R^NHCP^*t*Bu^=3‐alkyl/aryl‐1‐bis(di‐*tert*‐butylphosphino)‐imidazolin‐2‐ylidene; R=Me, Mes) and [{(^R^NHC(CH_2_)P^*t*Bu^)Cu}_2_]^2+^2 [PF_6_]^−^ (^R^NHC(CH_2_)P^*t*Bu^=3‐alkyl/aryl‐1‐(di‐*tert*‐butylphosphinomethyl)‐imidazolin‐2‐ylidene; R=Me, Mes) reported by Hofmann et al. (2.575–2.744 Å).^[61]^

The bis‐NHC complex [(Dipp_2_Im)_2_Cu]**FAP** (**5 a**) was formed in small amounts during the reaction of [(Dipp_2_Im)CuF] (**3**) with (C_2_F_5_)_3_PF_2_, as is evident from the ^1^H NMR spectrum. This complex was subsequently prepared from [{(Dipp_2_Im)Cu}_2_]^2+^2 **FAP** ^−^ (**4**) and the free carbene (Scheme 5). The ^1^H NMR resonances of **4** in CD_2_Cl_2_ are very broad at room temperature. On cooling, these signals sharpened and were well resolved at −40 °C (see Figure S33 of the Supporting Information). All signals of the carbene ligand, except those of the olefinic backbone, are split into two sets due to the coordination of one of the aromatic moieties to copper, which results in local asymmetry of the carbene moiety. This is also reflected in the ^13^C{^1^H} NMR spectrum, in which two sets of signals for all but the carbenic carbon atoms and the carbon atoms of the backbone were observed (see Figure S35 of the Supporting Information). In [D_8_]THF solution only one set of signals was observed for the Dipp_2_Im ligand, which may be rationalized by THF coordination with cleavage of the copper–aryl coordination. The doublets of the *i*Pr methyl groups of the carbene ligands in [D_8_]THF were observed at 1.25 and 1.27 ppm. The corresponding methine protons give rise to a septet at 2.63 ppm, and the protons of the phenyl groups were detected at 7.42 and 7.55 ppm. Additionally, the olefinic protons of the backbone were observed at 7.66 ppm.

**Scheme 6:**
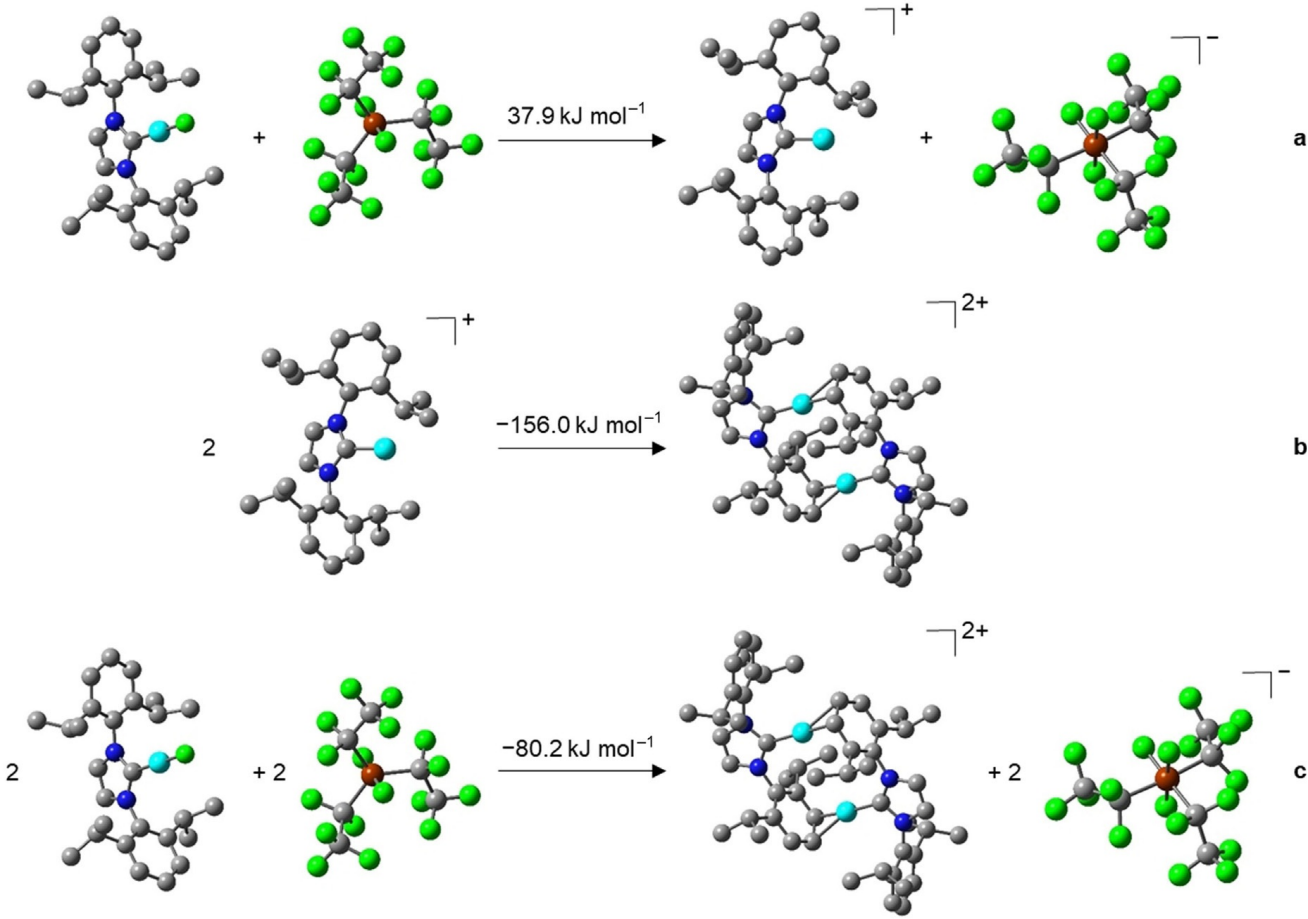
Optimized geometries (hydrogen atoms are omitted for clarity) and calculated reaction energies (DFT, PBE0/def2‐TZVP/COSMO) of the fluoride transfer from [(Dipp_2_Im)CuF] (**3**) to (C_2_F_5_)_3_PF_2_ and subsequent formation of [{(Dipp_2_Im)Cu}_2_]^2+^2 **FAP^−^** (**4**). The calculations demonstrate that the Dipp arene ligand of **4** is rather η^2^‐bonded to the adjacent Cu atom.

The ^19^F and ^31^P NMR spectrum of **4** show the presence of the *mer*‐isomer of the **FAP** anion, similar to all other complexes presented herein. The formation of the dinuclear species [{(Dipp_2_Im)Cu}_2_]^2+^ is in agreement with quantum chemical calculations (DFT, PBE0/def2‐TZVP/COSMO; see Scheme 6). The transfer of a fluoride anion from [(Dipp_2_Im)CuF] (**3**) to the phosphorane is an endothermic process (+37.9 kJ mol^−1^; see Scheme 6 a), but dimerization of two cations [(Dipp_2_Im)Cu]^+^ to form the dication of **4** is exothermic by −156.0 kJ mol^−1^ (−78 kJ mol^−1^ per copper atom, Scheme 6 b), and contributes significantly to the exothermicity (−80.2 kJ mol^−1^) of the overall process starting from [(Dipp_2_Im)CuF] (**3**) and the phosphorane to yield **4** (Scheme 6 c).

A solvent‐coordinated cationic fragment [(Dipp_2_Im)Cu(thf)]^+^ was detected at *m*/*z*=523.27 in the HR mass spectrum of **4** from THF solutions of this complex, which nicely demonstrates that dinuclear **4** serves as a source of the mononuclear [(Dipp_2_Im)Cu]^+^ cation. Thus, we treated complex **4** with PPh_3_ and selected carbenes and subsequently isolated the complexes [(Dipp_2_Im)Cu(NHC)]**FAP** (NHC=Dipp_2_Im, **5 a**; *t*Bu_2_Im, **5 b**; Me_2_Im^Me^, **5 c**; cAAC^Me^, **5 d**; *t*Bu_2_Im=1,3‐di(*tert*‐butyl)‐imidazolin‐2‐ylidene, Me_2_Im^Me^=1,3,4,5‐tetramethyl‐imidazolin‐2‐ylidene, cAAC^Me^=1‐(2,6‐di‐isopropylphenyl)‐2,2,4,4‐tetramethyl‐pyrrolidine) and [(Dipp_2_Im)Cu(PPh_3_)]**FAP** (**5 e**) (see Scheme 5). Complexes **5 a**–**e** were characterized by ^1^H, ^13^C, ^19^F, and ^31^P NMR spectroscopy, elemental analysis, IR spectroscopy, and mass spectrometry (see Supporting Information).

Table 1 summarizes selected chemical shifts of the ^1^H and ^13^C NMR spectra of **4** and **5 a**–**e** in [D_8_]THF solution. There are no significant differences in the ^19^F or ^31^P NMR spectra of any of the compounds.

**Table 1:** Selected ^1^H and ^13^C NMR chemical shifts [ppm] of the Dipp_2_Im ligands of **4** and **5 a**–**e** in [D_8_]THF.

	*δ*(^13^C)	*δ*(^1^H)				
	NCN	NCHCHN	C_*para*_−H	C_*meta*_−H	*i*Pr‐CH	*i*Pr‐CH_3_
**4**	180.1	7.56	7.42	7.42	2.63	1.27/1.25
**5 a**	178.1	7.38	7.48	7.20	2.37	1.02/0.90
**5 b**	179.8	7.75	7.55	7.43	2.79	1.27/1.23
**5 c**	180.7	7.75	7.56	7.43	2.64	1.27
**5 d**	178.8	7.61	7.52	7.34	2.52	1.14/1.13
**5 e**	178.0	7.81	7.65	7.46	2.63	1.26/1.15

**Figure 8:**
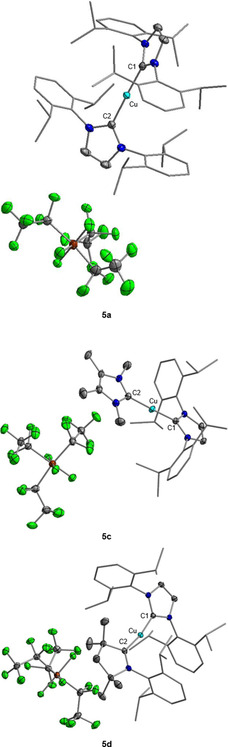
Molecular structures of [(Dipp_2_Im)_2_Cu]**FAP** (**5 a**) (top), [(Dipp_2_Im)Cu(Me_2_Im^Me^)]**FAP** (**5 c**) (middle), and [(Dipp_2_Im)Cu(cAAC^Me^)]**FAP** (**5 d**) (bottom) in the solid state (ellipsoids set at the 50 % probability level; *i*Pr and phenyl groups are shown as wire‐and‐stick models). Hydrogen atoms are omitted for clarity. Bond lengths [Å] and angles [°]: **5 a**: C1−Cu 1.9272(15), C2−Cu 1.9272(15); C1−Cu−C2 178.27(6); **5 c**: C1−Cu 1.8955(19), C2−Cu 1.898(2); C1‐Cu‐C2 176.23(8); **5 d**: C1−Cu 1.9129(14), C2−Cu 1.9136(14); C1‐Cu‐C2 170.41(6).

The cations of **5 a**,[^58^, ^62^] **5 b** ^[63]^ and **5 e** ^[64]^ were reported previously but synthesized by different routes. Crystals of **5 a**, **5 c**, and **5 d** suitable for X‐ray diffraction were obtained by evaporation of a solution of **5 a** in CH_2_Cl_2_ and vapor‐diffusion of hexane into solutions of **5 c** in THF and **5 d** in toluene (see Figure 8). Complex **5 a** crystallizes in the triclinic space group $P\bar{1}$ and compounds **5 c** and **5 d** crystallize in the monoclinic space group *P*2_1_/*n*. All three structures show the corresponding cations [(Dipp_2_Im)Cu(NHC)]^+^ and the *mer*‐isomer of the **FAP** anion. The copper atom in these complexes is linearly coordinated by the two NHC ligands. In **5 a**, **5 c**, and **5 d** the distances between the carbenic carbon atoms and the copper atom are the same within the standard deviation (**5 a**: C1−Cu=C2−Cu 1.9272(15) Å; **5 c**: C1−Cu 1.8955(19), C2−Cu 1.898(2) Å; **5 d**: C1−Cu 1.9129(14), C2−Cu 1.9136(14) Å). The distances between the carbenic carbon atoms and the copper atom in **5 c** are slightly shorter than those in **5 a** and **5 d**. The C−Cu distances in **5 a** are similar to those of [(Dipp_2_Im)_2_Cu][X] (X=PF_6_: 1.938(5) Å;^[58]^ BF_4_: 1.939(18) Å;^[58]^ BPh_4_: 1.872(4) Å;^[62a]^ Bneop_2_: 1.9204(14) Å;^[62b]^ Bpin_2_: 1.920(4) Å;^[62c]^ neop=neopentyl glycolato, pin=pinacolato) with the same cation but different counterions. The C1−Cu bond lengths in **5 c** and **5 d** are in the range of the distances within standard deviation of the carbenic carbon atoms of the Dipp_2_Im ligands to the copper atom in the heteroleptic bis‐NHC complexes reported by Cazin et al. (1.898(6)–1.908(7) Å).^[63]^ The C2−Cu distances in **5 c** (1.898(2) Å) and **5 d** (1.9136(14) Å) are slightly longer than *d*(C−Cu) in the related chloride complexes [(Me_2_Im^Me^)CuCl] (1.878(2) Å)^[65]^ and [(cAAC^Me^)CuCl] (1.878(2) Å).^[66]^ The corresponding cationic copper ions were detected in the high resolution mass spectra of **5 a**–**e** (**5 a**: *m*/*z* 839.50; **5 b**: *m*/*z* 631.38; **5 c**: *m*/*z* 575.31; **5 d**: *m*/*z* 736.46; **5 e**: *m*/*z* 729.30). Additionally, the signal of the **FAP** anion was observed in the negative‐ion mode of the mass spectra of the aforementioned salts (see Supporting Information).

The cationic hexamethylbenzene Cu^I^ complex of [(Dipp_2_Im)Cu(C_6_Me_6_)][SbF_6_]^[59]^ is accessible as [(Dipp_2_Im)Cu(C_6_Me_6_)]**FAP** (**5 f**) as the sole product starting from the fluoride complex [(Dipp_2_Im)CuF] (**3**), (C_2_F_5_)_3_PF_2_, and C_6_Me_6_. Complex **5 f** was also prepared by addition of C_6_Me_6_ to a solution of **4** in CD_2_Cl_2_. On dissolution of **5 f** in [D_8_]THF the signal of free hexamethylbenzene was observed at 2.17 ppm, which shows that hexamethylbenzene is easily replaced by other donors. In addition, solutions of both **4** and **5 f** in [D_8_]THF showed the same signals in the ^1^H NMR spectra. Consequently, in both **4** and **5 f** the aryl ligand can be substituted by other donors, which was also previously observed for the related complex [(Dipp_2_Im)Cu(toluene)][SbF_6_].^[67]^ However, fluoride abstraction with a fluoride‐ion acceptor such as (C_2_F_5_)_3_PF_2_ is advantageous compared to bromide abstraction with a silver salt of a WCA, since 1) it is a more atom‐efficient process, 2) the Lewis acid (C_2_F_5_)_3_PF_2_ is cheaper than silver salts of WCAs, and 3) it is not necessary to remove any byproduct such as AgBr.

### Titanium complexes

To demonstrate the scope of the phosphorane (C_2_F_5_)_3_PF_2_ as fluoride abstractor with respect to electron‐deficient early 3d metal fluoride complexes, we expanded our study to the model reaction of (C_2_F_5_)_3_PF_2_ with [Cp_2_TiF_2_] (**6**; Cp=η^5^‐C_5_H_5_). The Ti−F bond energy was experimentally found to be 569 kJ mol^−1^ in the gas phase.^[68]^ For Ni−F and Cu−F significantly smaller dissociation energies of 430^[69]^ and 413 kJ mol^−1 [70]^ have been reported, respectively. These experimental values are based on mass spectrometric data for Ti^[68]^ and Cu^[70]^ and on IR spectroscopic data for Ni.^[69]^ Thus, the abstraction of a fluoride ion from titanium should require a much stronger Lewis acid compared with copper or nickel.

**Scheme 7:**
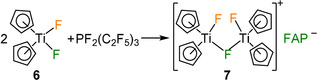
Synthesis of [FCp_2_Ti(*μ*‐F)TiCp_2_F]**FAP** (**7**).

After addition of one equivalent of (C_2_F_5_)_3_PF_2_ to a solution of **6** in CH_2_Cl_2_, the color immediately changed from yellow to orange. After 15 min of stirring, an orange solid precipitated. Removal of the solvent in vacuo led to isolation of the dinuclear complex [FCp_2_Ti(*μ*‐F)TiCp_2_F]**FAP** (**7**). Thus, only one of the fluoride ligands of **6** was transferred to the phosphorane. The outcome is independent of the stoichiometry used, as the reaction of **6** with 0.5 and 5 equiv of (C_2_F_5_)_3_PF_2_ also gave **7** as sole product. In the cationic complex **7** a fluoride ligand bridges two titanium atoms (Scheme 7). The formation of the *mer*‐**FAP** anion can be deduced from ^19^F and ^31^P NMR spectra. Besides the resonances of the *mer*‐**FAP** anion, additional signals were observed in the ^19^F NMR spectrum at 168.2 and −121.9 ppm for the terminal (168.2 ppm) and the bridging (−121.9 ppm) fluoride ligand(s),^[71]^ which is also in agreement with the integration of the ^19^F NMR spectrum of **7** (see Figure S63 of the Supporting Information).

The proposed structure of **7** was also confirmed by elemental analysis and the HR mass spectrum of the isolated complex. In the positive‐mode HR mass spectrum the dinuclear cation [FCp_2_Ti(*μ*‐F)TiCp_2_F]^+^ was detected at *m*/*z* 413.05. The signal of the **FAP** anion was observed at *m*/*z* 444.95. Additionally, crystals of **7** suitable for X‐ray diffraction were obtained by diffusion of hexane into a solution of **7** in 1,2‐difluorobenzene (see Figure S65 of the Supporting Information). Although the quality of the data is insufficient for a detailed analysis of the bonding parameters, the solid‐state structure proves the connectivity of the dinuclear cation featuring a bridging fluoride ligand and the **FAP** anion (*mer*‐isomer) as a not coordinated counterion.

**Scheme 8:**
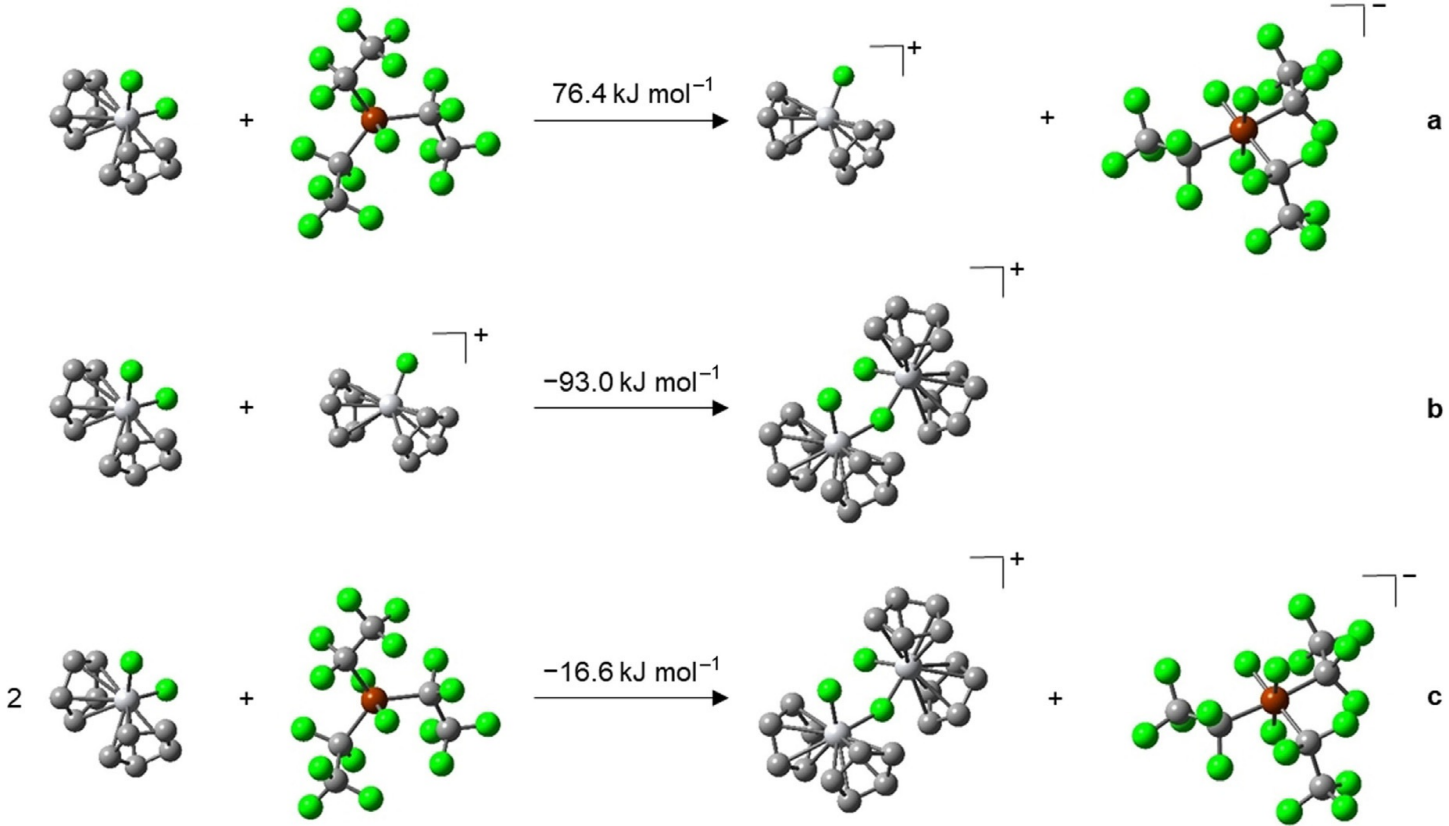
Optimized geometries (hydrogen atoms are omitted for clarity) and calculated reaction energies (DFT, PBE0/def2‐TZVP/COSMO) of the fluoride transfer from [Cp_2_TiF_2_] (**6**) to (C_2_F_5_)_3_PF_2_ and subsequent formation of [FCp_2_Ti(*μ*‐F)TiCp_2_F]**FAP** (**7** *)*.

The formation of a dinuclear complex is further supported by quantum chemical calculations (Scheme 8). Similar to the copper complex **3**, the formation of a non‐stabilized mononuclear cation [Cp2TiF]^+^ is thermodynamically not feasible, as fluoride transfer is highly endothermic (+76.4 kJ mol^−1^, see Scheme 8 a). As this process is more endothermic for titanium (76.4 kJ mol^−1^) than for copper (37.9 kJ mol^−1^), the metal–fluoride bond in **6** should be stronger than that in **3**. The addition of [Cp_2_TiF_2_] to the cation [Cp_2_TiF]^+^, however, proved to be crucial, as this addition is exothermic (−93.0 kJ mol^−1^; see Scheme 8 b) and provides the driving force necessary to make the overall reaction exothermic (−16.6 kJ mol^−1^; see Scheme 8 c).

## Conclusion

We have demonstrated that fluoride abstraction from 3d transition metal fluoride complexes by the readily available and easy‐to‐handle (liquid) strong Lewis acid tris(pentafluoroethyl)difluorophosphorane (C_2_F_5_)_3_PF_2_ is a general approach to prepare cationic complexes, as exemplified for fluoride complexes of nickel, copper, and titanium. The cationic metal complexes are stabilized by the weakly coordinating tris(pentafluoroethyl)trifluorophosphate anion (**FAP** anion, [(C_2_F_5_)_3_PF_3_]^−^). Fluoride abstraction from *trans*‐[Ni(*i*Pr_2_Im)_2_(Ar^F^)F] (**1 a**–**c**) led to a series of solvent‐coordinated cationic complexes or cations stabilized by classical Lewis bases if the reaction was conducted in the presence of a Lewis base such as a carbene or a phosphine. Fluoride abstraction from [(Dipp_2_Im)CuF] (**3**) resulted in the formation of the dinuclear species [{(Dipp_2_Im)Cu}_2_]^2+^2 **FAP^−^** (**4**), in which the copper complex cations are stabilized by copper–arene interactions with NHC aryl groups of the adjacent [(Dipp_2_Im)Cu]^+^ moiety. This weak interaction can be cleaved by weak two‐electron donors such as THF (as evidenced by HRMS and ^1^H NMR spectroscopy), but not by the weakly coordinating **FAP** anion. Reaction of **4** with Lewis bases such as a phosphine and carbenes resulted in the formation of complexes of the type [(Dipp_2_Im)Cu(LB)]**FAP** (**5 a**–**e**), indicating that the dinuclear complex **4** serves as a synthon for [(Dipp_2_Im)Cu]^+^. When fluoride transfer from [(Dipp_2_Im)CuF] (**3**) was conducted in the presence of C_6_Me_6_, the salt [(Dipp_2_Im)Cu(C_6_Me_6_)]**FAP** (**5 f**) was obtained in good yield. Fluoride abstraction from [Cp_2_TiF_2_] (**6**) with (C_2_F_5_)_3_PF_2_ afforded the dinuclear complex [FCp_2_Ti(μ‐F)TiCp_2_F]**FAP** (**7**), in which the two titanium centers are bridged by a fluoride ligand.

In all cases the phosphorane (C_2_F_5_)_3_PF_2_, which has a FIA of 405.4 kJ mol^−1^ close to that of AsF_5_ (FIA=427.6 kJ mol^−1^), serves as a versatile fluoride acceptor to give the weakly coordinating **FAP** anion. Fluoride abstraction is thus feasible for a wide range of transition metal complexes, including those of fluorophilic titanium, and the **FAP** anion stabilizes reactive cationic complexes, as exemplified by cationic copper, nickel, and titanium complexes. We believe that fluoride abstraction by the phosphorane (C_2_F_5_)_3_PF_2_ and cation stabilization with the resulting **FAP** anion formed are generally applicable for the stabilization of reactive transition metal complex cations and cationic intermediates, and future studies along these lines are in progress.

## Experimental Section

**Crystallographic data**: Deposition Number(s) 2042381 (for ***trans*****‐2c[OH_2_]⋅H_2_O**), 2042382 (for ***trans*****‐2a[PPh_3_]**), 2042386 (for ***cis*****‐2a[Dipp_2_Im]**), 2042385 (for **4**), 2042383 (for **5a**), 2042384 (for **5c**), and 2042387 (for **5d**) contain(s) the supplementary crystallographic data for this paper. These data are provided free of charge by the joint Cambridge Crystallographic Data Centre and Fachinformationszentrum Karlsruhe Access Structures service www.ccdc.cam.ac.uk/structures.

## Conflict of interest

The authors declare no conflict of interest.

## Supporting information

As a service to our authors and readers, this journal provides supporting information supplied by the authors. Such materials are peer reviewed and may be re‐organized for online delivery, but are not copy‐edited or typeset. Technical support issues arising from supporting information (other than missing files) should be addressed to the authors.

Supplementary
