# Supplementary material for: Tris(pentafluoroethyl)difluorophosphorane: A Versatile Fluoride Acceptor for Transition Metal Chemistry
Source: Chemistry. 2021 Jan 26;27(10):3504–16. doi: 10.1002/chem.202004885 (PMC7898530; doi:10.1002/chem.202004885)
Supplement: Supplementary file 1 — Supplementary [file CHEM-27-3504-s001.pdf]

Chemistry–A European Journal

Supporting Information

Tris(pentafluoroethyl)difluorophosphorane: A Versatile Fluoride Acceptor for Transition Metal Chemistry

Steffen A. Föhrenbacher,[a] Mirjam J. Krahfuss,[a] Ludwig Zapf,[a, b] Alexandra Friedrich,[a, b] Nikolai V. Ignat'ev,[a, b, c] Maik Finze,*[a, b] and Udo Radius*[a]

Contents

1) Experimental Section

2) NMR Spectra of Compounds

3) Additional Crystallographic Data

4) Additional Tables and Figures

5) Computational Details – Optimized Geometries

1) Experimental Section

General: Compounds **1a**–**c**, **3** and **6** were prepared according to literature procedures.[S1] Either commercially available $(C_2F_5)_3PF_2$ was used or the phosphorane was synthesized via electrochemical fluorination (ECF) starting from triethylphosphine as described in the literature.[S2] All other starting materials were purchased from commercial sources and used without further purification. All solvents for synthetic reactions were HPLC grade, further treated to remove traces of water using an Innovative Technology Inc. Pure-Solv Solvent Purification System. All reactions and subsequent manipulations were performed under an argon atmosphere in an Innovative Technology Inc. glovebox or using standard Schlenk techniques. NMR spectra were recorded on a Bruker Avance NEO 400, a Bruker Avance Nanobay 400 and a Bruker Avance 500 spectrometer, using d_8-THF or CD_2Cl_2 as solvent. The solid-state magic-angle spinning (MAS) NMR spectra were recorded on a Bruker DSX-400 solid state spectrometer and with a 4 mm o.d. rotor. The assignment of the ^{1}H NMR spectra was supported by $^{1}H,^{1}H$ and $^{13}C,^{1}H$ correlation experiments. ^{13}C NMR spectra were broad-band proton-decoupled ($^{13}C\{^{1}H\}$). Assignment of the ^{13}C NMR data was supported by $^{13}C,^{1}H$ correlation experiments. Chemical shifts are listed in parts per million (ppm) and were calibrated against residual solvent signals ($\delta(^{1}H)$: d_7-THF 3.58,1.72; $CDHCl_2$ 5.32; $\delta(^{13}C)$: d_8-THF 67.21, 25.31, CD_2Cl_2 53.84)[S3] or external $CFCl_3$ ($\delta(^{19}F)$: 0) and 85 % H_3PO_4 ($\delta(^{31}P)$: 0). If not otherwise noted ^{19}F and ^{31}P NMR spectra were not proton-decoupled. Coupling constants are quoted in Hertz. IR spectra were recorded as solid samples on a Bruker Alpha FT-IR spectrometer using an ATR unit at room temperature. Values are given in cm^{-1}. Elemental analyses were performed in the microanalytical laboratory of the Institute of Inorganic Chemistry of the University of Würzburg with an Elementar vario micro cube. High-resolution mass spectrometry analyses were performed with a Thermo Scientific Exactive Plus mass spectrometer, equipped with an Orbitrap Mass Analyzer. Thermal analyses were conducted with a DSC 204 F1 Phoenix (Netzsch) in the temperature range of –20 to 500 °C with a heating rate of 10 K/min.

Preparation of the Complexes: Although the solvent coordinated complexes **2a**–**c[solv]** were formed, the d_8-THF coordinated compounds **2a**–**c[*d*8-thf]** were observed by NMR spectroscopy, as d_8-THF substitutes the coordinating solvent in solution. In the $^{13}C\{^{1}H\}$ NMR spectra of these compounds the resonances of the fluoroaryl ligands

were typically not observed under the conditions of the measurement. The signals of the carbon atoms of the perfluorinated alkyl groups of the **FAP** anion were also not observed.

trans*-[Ni(*i*Pr$_2$Im)$_2$(OEt$_2$)(C$_6$F$_5$)]FAP (2a[OEt$_2$]):** The phosphorane $(C_2F_5)_3PF_2$ (70.0 µL, 297 µmol) was added at room temperature to a suspension of **1a** (162 mg, 295 µmol) in Et_2O (7.5 mL). Upon addition the cloudy suspension cleared up immediately. The solution was stirred for 20 min at room temperature. All volatiles were removed under reduced pressure and the remaining solid was dried *in vacuo* yielding **2a[OEt$_2$]** (214 mg, 204 µmol, 69 %) as a yellow solid. ^{1}H NMR (500.1 MHz, d_8-THF, 298 K): δ = 7.40 (s, 4H, NC*H*C*H*N), 6.18 (sept, 4H, $^3J_{H-H}$ = 6.7 Hz, *i*Pr-C*H*), 3.38 (q, 4H, $^3J_{H-H}$ = 7.0 Hz, CH$_3$-C*H*$_2$), 1.57 (d, 12H, $^3J_{H-H}$ = 6.7 Hz, *i*Pr-C*H*$_3$), 1.36 (d, 12H, $^3J_{H-H}$ = 6.7 Hz, *i*Pr-C*H*$_3$), 1.11 (t, 6H, $^3J_{H-H}$ = 7.0 Hz, CH$_2$-C*H*$_3$); ^{13}C{^{1}H} NMR (125.8 MHz, d_8-THF, 298 K): δ = 164.5 (N*C*N), 120.0 (N*C*H*C*HN), 66.1 (CH$_3$-*C*H$_2$), 53.6 (*i*Pr-*C*H), 23.9 (*i*Pr-*C*H$_3$), 22.9 (*i*Pr-*C*H$_3$), 15.5 (CH$_2$-*C*H$_3$); ^{19}F NMR (470.6 MHz, d_8-THF, 298 K): δ = −45.1 (dm, 1F, $^1J_{P-F}$ = 891 Hz, P*F*), −80.7 (m, 3F, C*F*$_3$), −82.4 (m, 6F, C*F*$_3$), −88.0 (dm, 2F, $^1J_{P-F}$ = 905 Hz, P*F*$_2$), −116.4 (dm, 2F, $^2J_{P-F}$ = 83 Hz, C*F*$_2$), −117.0 (dm, 4F, $^2J_{P-F}$ = 97 Hz, C*F*$_2$), −117.7 (m, 2F, aryl-C$_{ortho}$*F*), −162.5 (t, 1F, $^3J_{F-F}$ = 19.8 Hz, aryl-C$_{para}$*F*), −165.1 (m, 2F, aryl-C$_{meta}$*F*); ^{31}P NMR (202.4 MHz, d_8-THF, 298 K): δ = −148.2 (tdm, $^1J_{P-F}$ = 905 Hz, $^1J_{P-F}$ = 891 Hz); IR ([cm^{-1}]): 2985 (w), 2924 (vw), 2883 (vw), 1631 (vw), 1568 (vw), 1504 (m), 1453 (m), 1442 (m), 1426 (w), 1412 (w), 1398 (m), 1377 (m), 1296 (m), 1208 (vs), 1184 (vs), 1128 (s), 1098 (s), 1058 (s), 998 (vw), 955 (s), 897 (vw), 882 (vw), 815 (m), 784 (w), 762 (w), 719 (m), 706 (m), 673 (w), 637 (w), 618 (vs), 579 (m), 553 (w), 496 (w), 451 (w), 438 (w), 429 (w); HRMS (ESI) *m/z* [*M*−OEt$_2$]$^+$ calcd. for $C_{24}H_{32}F_5N_4Ni$: 548.3352, found: 548.3331; *m/z* ***FAP$^-$ calcd. for $C_6F_{18}P$: 444.9450, found: 444.9440; elemental analysis calcd. (%) for $C_{34}H_{42}F_{23}N_4NiOP$: C 38.92, H 4.03, N, 5.34; found C 38.17, H 4.10, N 5.32.

***trans*-[Ni(*i*Pr$_2$Im)$_2$(ClCH$_2$Cl)(C$_6$F$_5$)]FAP (2a[ClCH$_2$Cl]):** The phosphorane $(C_2F_5)_3PF_2$ (270 µL, 1.15 mmol) was added at room temperature to a solution of **1a** (646 mg, 1.18 mmol) in CH_2Cl_2 (15 mL). The solution was stirred for 2 h at room temperature. All volatiles were removed under reduced pressure and the remaining solid was dried *in vacuo* yielding **2a[ClCH$_2$Cl]** (1.01 g, 962 µmol, 81 %) as a yellow solid. ^{1}H NMR (500.1 MHz, d_8-THF, 298 K): δ = 7.39 (s, 4H, NC*H*C*H*N), 6.17 (sept, 4H, $^3J_{H-H}$ = 6.7 Hz, *i*Pr-C*H*), 5.49 (s, 2H, C*H*$_2$Cl$_2$), 1.57 (d, 12H, $^3J_{H-H}$ = 6.7 Hz, C*H*$_3$), 1.36 (d, 12H, $^3J_{H-H}$ =

6.7 Hz, *i*Pr-C*H*$_3$); ^{13}C{^{1}H} NMR (125.8 MHz, *d*$_8$-THF, 298 K): δ = 164.5 (N*C*N), 120.0 (N*C*H*C*HN), 54.7 (s, *C*H$_2$Cl$_2$), 53.6 (*i*Pr-*C*H), 23.9 (*i*Pr-*C*H$_3$), 22.9 (*i*Pr-*C*H$_3$); ^{19}F NMR (470.6 MHz, *d*$_8$-THF, 298 K): δ = −45.1 (dm, 1F, $^1J_{P-F}$ = 891 Hz, P*F*), −80.8 (m, 3F, C*F*$_3$), −82.4 (m, 6F, C*F*$_3$), −88.0 (dm, 2F, $^1J_{P-F}$ = 905 Hz, P*F*$_2$), −116.4 (dm, 2F,$^2J_{P-F}$ = 83 Hz, C*F*$_2$), −117.0 (dm, 4F, $^2J_{P-F}$ = 97 Hz, C*F*$_2$), −117.8 (m, 2F, aryl-C$_{ortho}$*F*), −162.5 (t, 1F, $^3J_{F-F}$ = 19.8 Hz, aryl-C$_{para}$*F*), −165.1 (m, 2F, aryl-C$_{meta}$*F*); ^{31}P NMR (202.4 MHz, *d*$_8$-THF, 298 K): δ = −148.2 (tdm, $^1J_{P-F}$ = 905 Hz, $^1J_{P-F}$ = 891 Hz); IR ([cm^{-1}]): 3185 (vw), 2985 (w), 2944 (vw), 2883 (vw), 1623 (w), 1568 (vw), 1505 (m), 1453 (m), 1440 (w), 1428 (w), 1412 (w), 1398 (m), 1377 (m), 1302 (m), 1270 (w), 1209 (vs), 1182 (vs), 1137 (s), 1129 (s), 1090 (s), 1057 (s), 1001 (w), 971 (m), 954 (s), 883 (vw), 824 (m), 785 (m), 765 (w), 751 (vw), 742 (w), 727 (m), 702 (s), 682 (s), 637 (w), 617 (vs), 579 (m), 535 (m), 505 (w), 495 (w), 450 (w), 440 (w), 429 (m); HRMS (ESI) *m/z* [*M*−CH$_2$Cl$_2$]$^+$ calcd. for C$_{24}$H$_{32}$F$_5$N$_4$Ni: 529.1901, found: 529.1889; *m/z* ***FAP***$^-$ calcd. for C$_6$F$_{18}$P: 444.9450, found: 444.9436; elemental analysis calcd. (%) for C$_{31}$H$_{34}$Cl$_2$F$_{23}$N$_4$NiP: C 35.12, H 3.23, N, 5.28; found C 35.33, H 3.52, N 5.90.

***trans*-[Ni(*i*Pr$_2$Im)$_2$(thf)(C$_6$F$_5$)]FAP (2a[thf]):** The phosphorane (C$_2$F$_5$)$_3$PF$_2$ (45.0 µL, 191 µmol) was added at room temperature to a solution of **1a** (100 mg, 182 µmol) in a mixture of Et$_2$O (3 mL) and THF (3 mL). The solution was stirred for 2 h at room temperature. All volatiles were removed under reduced pressure and the remaining solid was dried *in vacuo* yielding **2a[thf]** (165 mg, 158 µmol, 87 %) as a yellow solid. ^{1}H NMR (500.1 MHz, CD$_2$Cl$_2$, 298 K): δ = 7.04 (s, 4H, NC*H*C*H*N), 6.11 (sept, 4H, $^3J_{H-H}$ = 6.7 Hz, *i*Pr-C*H*), 3.20 (m, 4H, thf-C$_{2,5}$*H*$_2$), 1.83 (m, 4H, thf-C$_{3,4}$*H*$_2$), 1.54 (d, 12H, $^3J_{H-H}$ = 6.7 Hz, *i*Pr-C*H*$_3$), 1.35 (d, 12H, $^3J_{H-H}$ = 6.7 Hz, C*H*$_3$); ^{13}C{^{1}H} NMR (125.8 MHz, CD$_2$Cl$_2$, 298 K): δ = 164.4 (N*C*N), 119.2 (N*C*H*C*HN), 74.1 (THF-*C*$_{2,5}$), 53.4 (*i*Pr-*C*H), 25.6 (THF-*C*$_{3,4}$), 24.4 (*i*Pr-*C*H$_3$), 23.3 (*i*Pr-*C*H$_3$); ^{19}F NMR (470.6 MHz, CD$_2$Cl$_2$, 298 K): δ = −45.1 (dm, 1F, $^1J_{P-F}$ = 890 Hz, P*F*), −80.6 (m, 3F, C*F*$_3$), −82.3 (m, 6F, C*F*$_3$), −88.5 (dm, 2F, $^1J_{P-F}$ = 906 Hz, P*F*$_2$), −116.1 (dm, 2F, $^2J_{P-F}$ = 83 Hz, C*F*$_2$), −116.7 (dm, 4F, $^2J_{P-F}$ = 98 Hz, C*F*$_2$), −117.9 (m, 2F, aryl-C$_{ortho}$*F*), −160.6 (t, 1F, $^3J_{F-F}$ = 19 8 Hz, aryl-C$_{para}$*F*), −163.9 (m, 2F, aryl-C$_{meta}$*F*); ^{31}P NMR (202.4 MHz, CD$_2$Cl$_2$, 298 K): δ = −147.6 (tdm, $^1J_{P-F}$ = 905 Hz, $^1J_{P-F}$ = 891 Hz); IR ([cm^{-1}]): 2983 (vw), 2939 (vw), 1570 (vw), 1503 (w), 1455 (w), 1440 (w), 1409 (w), 1397 (w), 1376 (w), 1311 (w), 1299 (w), 1221 (m), 1207 (s), 1195 (s), 1186 (s), 1144 (w), 1134 (m), 1089 (m), 1060 (m), 1028 (w), 956 (m), 869 (w), 814 (m), 783 (w), 765 (m), 737 (w), 726 (s), 703 (m), 673 (vw), 636 (w), 617 (vs),

579 (w), 534 (w), 505 (w), 496 (w), 448 8w), 429 (w), 422 (w); HRMS (ESI) *m/z* [*M*−thf]$^+$ calcd. for $C_{24}H_{32}F_5N_4Ni$: 529.1901, found: 529.1897; *m/z* ***FAP***$^-$ calcd. for $C_6F_{18}P$: 444.9450, found: 444.9438; elemental analysis calcd. (%) for $C_{34}H_{40}F_{23}N_4NiOP$: C 38.99, H 3.85, N, 5.35; found C 38.39, H 3.98, N 5.35.

trans*-[Ni(*i*Pr$_2$Im)$_2$(OEt$_2$)(4-CF$_3$-C$_6$F$_4$)]FAP (2b[OEt$_2$]):** The phosphorane $(C_2F_5)_3PF_2$ (50.0 µL, 212 µmol) was added at room temperature to a suspension of **1b** (125 mg, 209 µmol) in Et_2O (5 mL). Upon addition the cloudy suspension cleared up immediately. The solution was stirred for 2 h at room temperature. All volatiles were removed under reduced pressure and the remaining solid was dried *in vacuo* yielding **2b[OEt$_2$]** (117 mg, 106 µmol, 51 %) as a yellow solid. ^{1}H NMR (500.1 MHz, d_8-THF, 298 K): δ = 7.43 (s, 4H, NC*H*C*H*N), 6.17 (sept, 4H, $^3J_{H\text{-}H}$ = 6.7 Hz, *i*Pr-C*H*), 3.38 (q, 4H, $^3J_{H\text{-}H}$ = 7.0 Hz, CH_3-C*H*$_2$), 1.58 (d, 12H, $^3J_{H\text{-}H}$ = 6.7 Hz, *i*Pr-C*H*$_3$), 1.38 (d, 12H, $^3J_{H\text{-}H}$ = 6.7 Hz, C*H*$_3$), 1.10 (t, 6H, $^3J_{H\text{-}H}$ = 7.0 Hz, CH_2-C*H*$_3$); ^{13}C{^{1}H} NMR (125.8 MHz, d_8-THF, 298 K): δ = 163.6 (N*C*N), 120.2 (N*C*H*C*HN), 66.1 (s, CH_3-*C*H$_2$), 53.7 (*i*Pr-*C*H), 23.9 (*i*Pr-*C*H$_3$), 22.9 (*i*Pr-*C*H$_3$), 15.5 (CH_2-*C*H$_3$); ^{19}F NMR (470.6 MHz, d_8-THF, 298 K): δ = −45.1 (dm, 1F, $^1J_{P\text{-}F}$ = 891 Hz, P*F*), −56.8 (t, 3F, $^4J_{F\text{-}F}$ = 21.1 Hz, aryl-C*F*$_3$), −80.8 (m, 3F, CF_2-C*F*$_3$), −82.4 (m, 6F, CF_2-C*F*$_3$), −88.0 (dm, 2F, $^1J_{P\text{-}F}$ = 905 Hz, P*F*$_2$), −116.4 (dm, 2F, $^2J_{P\text{-}F}$ = 83 Hz, C*F*$_2$), −116.8 (m, 2F, aryl-C_{ortho}*F*), −117.0 (dm, 4F, $^2J_{P\text{-}F}$ = 97 Hz, C*F*$_2$), −145.5 (dm, 2F, aryl-C_{meta}F); ^{31}P NMR (202.4 MHz, d_8-THF, 298 K): δ = −148.2 (tdm, $^1J_{P\text{-}F}$ = 905 Hz, $^1J_{P\text{-}F}$ = 891 Hz); IR ([cm^{-1}]): 3184 (vw), 2986 (w), 2944 (vw), 1624 (w), 1568 (vw), 1501 (vw), 1439 (m), 1398 (v), 1377 (m), 1311 (s), 1209 (vs), 1183 (vs), 1128 (vs), 1096 (m), 1085 (m), 1044 (w), 996 (vw), 957 (s), 915 (w), 895 (vw), 880 (w), 819 (m), 780 (w), 762 (m), 722 (s), 708 (s), 674 (vw), 637 (w), 617 (vs), 579 (m), 534 (w), 505 (w), 496 (w), 456 (vw), 429 (w); HRMS (ESI) *m/z* [*M*−OEt$_2$]$^+$ calcd. for $C_{25}H_{32}F_7N_4Ni$: 579.1869, found: 579.1852; *m/z* ***FAP$^-$ calcd. for $C_6F_{18}P$: 444.9450, found: 444.9436; elemental analysis calcd. (%) for $C_{35}H_{42}F_{25}N_4NiOP$: C 38.24, H 3.85, N, 5.10; found C 38.18, H 3.87, N 5.23.

***trans*-[Ni(*i*Pr$_2$Im)$_2$(OEt$_2$)(4-C$_6$F$_5$-C$_6$F$_4$)]FAP (2c[OEt$_2$]):** The phosphorane $(C_2F_5)_3PF_2$ (140 µL, 595 µmol) was added at room temperature to a suspension of **1c** (401 mg, 575 µmol) in Et_2O (8 mL). Upon addition, the cloudy suspension cleared up immediately. The solution was stirred for 2 h at room temperature. All volatiles were removed under reduced pressure and the remaining solid was dried *in vacuo* yielding **2c[OEt$_2$]** (610 mg, 509 µmol, 89 %) as a yellow solid. ^{1}H NMR (500.1 MHz, d_8-THF,

298 K): δ = 7.43 (s, 4H, NC*H*C*H*N), 6.17 (sept, 4H, $^3J_{H-H}$ = 6.6 Hz, *i*Pr-C*H*), 3.38 (q, 4H, $^3J_{H-H}$ = 7.0 Hz, CH_3-CH_2), 1.59 (d, 12H, $^3J_{H-H}$ = 6.6 Hz, CH_3), 1.38 (d, 12H, $^3J_{H-H}$ = 6.6 Hz, CH_3), 1.11 (t, 6H, $^3J_{H-H}$ = 7.0 Hz, CH_2-CH_3); ^{13}C{^{1}H} NMR (125.8 MHz, *d*$_8$-THF): δ = 164.3 (N*C*N), 120.1 (N*C*H*C*HN), 66.1 (CH_3-*C*H_2), 53.7 (*i*Pr-*C*H), 23.9 (*i*Pr-CH_3), 22.8 (*i*Pr-*C*H_3), 15.5 (CH_2-*C*H_3); ^{19}F NMR (470.6 MHz, *d*$_8$-THF, 298 K): δ = −45.1 (dm, 1F, $^1J_{P-F}$ = 891 Hz, P*F*), −80.8 (m, 3F, CF_3), −82.4 (m, 6F, CF_3), −88.0 (dm, 2F, $^1J_{P-F}$ = 905 Hz, PF_2), −116.4 (dm, 2F, $^2J_{P-F}$ = 83 Hz, CF_2), −117.0 (dm, 4F, $^2J_{P-F}$ = 97 Hz, CF_2), −117.7 (m, 2F, aryl-$C_{2,6}$*F*), −140.5 (m, 2F, aryl-$C_{2',6'}$*F*), −143.1 (m, 2F, aryl-$C_{3,5}$*F*), −154.1 (t, 1F, $^3J_{F-F}$= 20.7 Hz, aryl-$C_{4'}$*F*) −163.7 (m, 2F, aryl-$C_{3',5'}$*F*); ^{31}P NMR (202.4 MHz, *d*$_8$-THF, 298 K): δ = −148.2 (tdm, $^1J_{P-F}$ = 905 Hz, $^1J_{P-F}$ = 891 Hz); IR ([cm^{-1}]): 3183 (vw), 2984 (w), 2944 (vw), 1634 (w), 1568 (w), 1528 (w), 1499 (m), 1469 (w), 1431 (m), 1398 (w), 1378 (w), 1297 (w), 1209 (vs), 1184 (s), 1127 (m), 1098 (m), 1069 (w), 1031(vw), 995 (w), 973 (w), 945 (m), 882(vw), 815 (m), 764 (w), 717 (s), 705 (s), 672 (w), 637 (w), 618 (vs), 580 (m), 533 (w), 505 (vw), 495 (vw), 438 (vw), 429 (vw); HRMS (ESI) *m/z* [*M*−OEt_2]$^+$ calcd. for $C_{30}H_{32}F_9N_4Ni$: 677.1837, found: 677.1813. *m/z* ***FAP***$^-$ calcd. for $C_6F_{18}P$: 444.9450, found: 444.9428; elemental analysis calcd. (%) for $C_{40}H_{42}F_{27}N_4NiOP$: C 40.12, H 3.54, N, 4.68; found C 39.48, H 3.49, N 4.21.

[{($Dipp_2Im$)Cu}$_2$]$^{2+}$2FAP$^-$ (4): The phosphorane $(C_2F_5)_3PF_2$ (340 µL, 1.44 mmol) was added at room temperature to a solution of **3** (685 mg, 1.45 mmol) in CH_2Cl_2 (10 mL). The colorless solution was stirred for 2 h at room temperature. All volatiles were removed under reduced pressure and the remaining solid was dried *in vacuo* yielding **4** (1.16 g, 649 µmol, 89 %) as a colorless solid. ^{1}H NMR (500.1 MHz, CD_2Cl_2, 258°K): δ = 7.77 (t, 2H, $^3J_{H-H}$ = 7.7 Hz, aryl-C_{para}*H*), 7.52 (d, 4H, $^3J_{H-H}$ = 7.7 Hz, aryl-C_{meta}*H*), 7.43 (br, 2H, NC*H*), 7.31 (br, 2H, NC*H*), 7.28 (d, 4H, $^3J_{H-H}$ = 6.8 Hz, aryl-C_{meta}*H*), 6.55 (t, 2H, $^3J_{H-H}$ = 7.7 Hz, aryl-C_{para}*H*), 2.28 (sept, 4H, $^3J_{H-H}$ = 6.8 Hz, *i*Pr-C*H*), 2.18 (sept, 4H, $^3J_{H-H}$ = 6.8 Hz, *i*Pr-C*H*), 1.33 (d, 12H, $^3J_{H-H}$ = 6.8 Hz, *i*Pr-CH_3), 1.20 (d, 12H, $^3J_{H-H}$ = 6.8 Hz, *i*Pr-CH_3), 1.09 (d, 12H, $^3J_{H-H}$ = 6.8 Hz, *i*Pr-CH_3), 1.07 (d, 12H, $^3J_{H-H}$ = 6.8 Hz, *i*Pr-CH_3); ^{13}C{^{1}H} NMR (125.8 MHz, CD_2Cl_2, 258°K): δ = 176.2 (N*C*N), 144.6 (aryl-C_{ortho}), 141.8 (aryl-C_{ortho}), 133.2 (aryl-C_{ipso}), 132.1 (aryl-C_{para}), 131.1 (aryl-C_{para}), 127.8 (N*C*H), 126.6 (aryl-C_{ipso}), 124.8 (aryl-C_{meta}), 124.4 (N*C*H), 122.5 (aryl-C_{meta}), 29.3 (*i*Pr-*C*H), 28.9 (*i*Pr-*C*H), 24.8 (*i*Pr-*C*H_3), 24.1 (*i*Pr-*C*H_3), 23.4 (*i*Pr-*C*H_3), 23.2 (*i*Pr-*C*H_3); ^{19}F NMR (470.6 MHz, CD_2Cl_2, 258°K): δ = −45.7 (dm, 1F, $^1J_{P-F}$ = 890 Hz, P*F*), −80.6 (m, 3F, CF_3), −82.3 (m, 6F, CF_3), −89.5 (dm, 2F, $^1J_{P-F}$ = 900 Hz, PF_2), −116.8 (br., 6F, CF_2);

^{31}P NMR (202.4 MHz, CD_2Cl_2, 258°K): δ = −148.0 (tdm, $^1J_{P\text{-}F}$ = 900 Hz, $^1J_{P\text{-}F}$ = 890 Hz); IR ([cm^{-1}]): 3146 (vw), 2967 (m), 2930 (w), 2875 (w), 1623 (vw), 1594 (vw), 1552 (vw), 1469 (m), 1414 (w), 1386 (w), 1367 (w), 1296 (m), 1257 (w), 1213 (vs), 1185 (vs), 1140 (s), 1128 (s), 1101 (s), 1061 (m), 966 (m), 936 (w), 804 (m), 759 (m), 725 (m), 700 (m), 637 (m), 619 (vs), 581 (m), 533 (m), 494 (w), 465 (vw), 438 (w), 430 (w); HRMS (ESI) *m*/*z* [*M*+THF]$^+$ calcd. for $C_{31}H_{44}CuN_2O$: 523.2750, found: 523.2737; *m/z* ***FAP***$^-$ calcd. for $C_6F_{18}P$: 444.9450, found: 444.9430; elemental analysis calcd. (%) for $C_{66}H_{72}Cu_2F_{36}N_4P_2$: C 44.18, H 4.04, N, 3.12; found C 43.79, H 4.41, N 3.41.

[(Dipp$_2$Im)$_2$Cu]FAP (5a): 4 (100 mg, 56.0 µmol) and Dipp$_2$Im (43.0 mg, 111 µmol) were dissolved in EtOH (2 mL) at room temperature. The suspension was stirred for 15 min at room temperature and the suspension was filtered. All volatiles of the filtrate were removed under reduced pressure and the remaining solid was dried *in vacuo* yielding **5a** (50.0 mg, 38.9 µmol, 35 %) as a colorless solid. ^{1}H NMR (500.1 MHz, d_8-THF, 298°K): δ = 7.48 (t, 4H, $^3J_{H\text{-}H}$ = 7.8 Hz, aryl-C$_{para}$*H*), 7.38 (s, 4H, NC*H*C*H*N), 7.20 (d, 8H, $^3J_{H\text{-}H}$ = 7.8 Hz, aryl-C$_{meta}$*H*), 2.37 (sept, 8H, $^3J_{H\text{-}H}$ = 6.9 Hz, *i*Pr-C*H*), 1.02 (d, 24H, $^3J_{H\text{-}H}$ = 6.9 Hz, *i*Pr-C*H*$_3$), 0.90 (d, 24H, $^3J_{H\text{-}H}$ = 6.9 Hz, *i*Pr-C*H*$_3$); ^{13}C{^{1}H} NMR (125.8 MHz, d_8-THF, 298°K): δ = 178.1 (NCN), 145.9 (aryl-*C*$_{ortho}$), 135.9 (aryl-*C*$_{ipso}$), 131.3 (aryl-*C*$_{para}$), 126.5 (N*C*H*C*HN), 125.2 (aryl-*C*$_{meta}$), 29.4 (*i*Pr-*C*H), 24.7 (*i*Pr-*C*H$_3$), 24.2 (*i*Pr-*C*H$_3$); ^{19}F NMR (470.6 MHz, d_8-THF, 298°K): δ = −45.2 (dm, 1F, $^1J_{P\text{-}F}$ = 891 Hz, P*F*), −80.7 (m, 3F, C*F*$_3$), −82.4 (m, 6F, C*F*$_3$), −88.0 (dm, 2F, $^1J_{P\text{-}F}$ = 905 Hz, P*F*$_2$), −116.4 (dm, 2F, $^2J_{P\text{-}F}$ = 82 Hz, C*F*$_2$), −117.0 (dm, 4F, $^2J_{P\text{-}F}$ = 97 Hz, C*F*$_2$); ^{31}P NMR (202.4 MHz, d_8-THF, 298°K): δ = −148.2 (tdm, $^1J_{P\text{-}F}$ = 905 Hz, $^1J_{P\text{-}F}$ = 891 Hz); IR ([cm^{-1}]): 3180 (vw), 2965 (w), 2872 (vw), 1596 (vw) 1566 (vw), 1462 (w), 1404 (w), 1387 (w), 1366 (vw), 1328 (w), 1310 (w), 1295 (w), 1216 (vs), 1189 (s), 1145 (m), 1138 (m), 1127 (m), 1096 (m), 1067 (w), 974 (w), 960 (w), 945 (w), 854 (vw), 814 (m), 805 (m), 760 (s), 721 (s), 702 (w), 682 (vw), 638 (w), 620 (vs), 581 (w), 551 (vw), 533 (vw), 495 (w), 456 (w), 438 (w), 429 (w); HRMS (ESI) *m*/*z* [*M*]$^+$ calcd. for $C_{54}H_{72}CuN_4$: 839.5053, found: 839.5032; *m/z* ***FAP***$^-$ calcd. for $C_6F_{18}P$: 444.9450, found: 444.9435; elemental analysis calcd. (%) for $C_{60}H_{72}CuF_{18}N_4P$: C 56.05, H 5.64, N, 4.36; found C 55.78, H 5.64, N 4.61.

[(Dipp$_2$Im)Cu(*t*Bu$_2$Im)]FAP (5b): 4 (106 mg, 59.1 µmol) and *t*Bu$_2$Im (21.3 mg, 118 µmol) were dissolved in EtOH (1 mL) at room temperature. The suspension was stirred for 15 min at room temperature, filtered and the filtrate stored at −30 °C.

Overnight, a colorless solid precipitated. The supernatant solution was decanted off and the remaining solid was dried *in vacuo* yielding **5b** (50.0 mg, 46.4 μmol, 39 %) as a colorless solid. ^{1}H NMR (500.1 MHz, d_8-THF, 298°K): δ = 7.75 (s, 2H, Dipp$_2$Im-NC*H*C*H*N), 7.55 (t, 2H, $^{3}J_{H-H}$ = 7.8 Hz, aryl-C$_{para}$*H*), 7.43 (d, 4H, $^{3}J_{H-H}$ = 7.8 Hz, aryl-C$_{meta}$*H*), 7.21 (s, 2H, *t*Bu$_2$Im-NC*H*C*H*N), 2.79 (sept, 4H, $^{3}J_{H-H}$ = 6.9 Hz, *i*Pr-C*H*), 1.27 (d, 12H, $^{3}J_{H-H}$ = 6.9 Hz, *i*Pr-C*H*$_3$), 1.23 (d, 12H, $^{3}J_{H-H}$ = 6.9 Hz, *i*Pr-C*H*$_3$), 1.22 (s, 18H, C(C*H*$_3$)$_3$); ^{13}C{^{1}H} NMR (125.8 MHz, d_8-THF, 298°K): δ = 179.8 (Dipp$_2$Im-N*C*N), 171.8 (*t*Bu$_2$Im-N*C*N), 146.2 (aryl-*C*$_{ortho}$), 136.0 (aryl-*C*$_{ipso}$), 131.4 (aryl-*C*$_{para}$), 125.8 (Dipp$_2$Im-N*C*H*C*HN), 125.4 (aryl-*C*$_{meta}$), 118.4 (*t*Bu$_2$Im-N*C*H*C*HN), 57.7 (*C*(CH$_3$)$_3$), 31.9 (C(*C*H$_3$)$_3$), 29.5 (*i*Pr-*C*H), 24.6 (*i*Pr-*C*H$_3$), 23.9 (*i*Pr-*C*H$_3$); ^{19}F NMR (470.6 MHz, d_8-THF, 298°K): δ = −45.1 (dm, 1F, $^{1}J_{P-F}$ = 891 Hz, P*F*), −80.7 (m, 3F, C*F*$_3$), −82.4 (m, 6F, C*F*$_3$), −88.0 (dm, 2F, $^{1}J_{P-F}$ = 905 Hz, P*F*$_2$), −116.4 (dm, 2F, $^{2}J_{P-F}$ = 82 Hz, C*F*$_2$), −117.0 (dm, 4F, $^{2}J_{P-F}$ = 97 Hz, C*F*$_2$); ^{31}P NMR (202.4 MHz, d_8-THF, 298°K): δ = −148.2 (tdm, $^{1}J_{P-F}$ = 905 Hz, $^{1}J_{P-F}$ = 891 Hz); IR ([cm^{-1}]): 3173 (vw), 3140 (vw), 2965 (w), 2875 (vw), 1594 (vw), 1561 (vw), 1468 (w), 1406 (w), 1388 (w), 1375 (w), 1350 (vw), 1296 (m), 1211 (w), 1181 (vs), 1145 (vs), 1136 (m), 1125 (m), 1099 (s), 1071 (m), 973 (w), 961 (w), 946 (w), 809 (s), 763 (m), 719 (s), 702 (w), 655 (vw), 636 (w), 618 (vs), 581 (w), 533 (w), 495 (w), 455 (vw), 438 (w), 429 (w); HRMS (ESI) *m/z* [*M*]$^{+}$ calcd. for $C_{38}H_{56}CuN_4$: 631.3801, found: 631.3787; *m/z* ***FAP***$^{-}$ calcd. for $C_6F_{18}P$: 444.9450, found: 444.9436; elemental analysis calcd. (%) for $C_{44}H_{56}CuF_{18}N_4P$: C 49.05, H 5.24, N, 5.20; found C 49.02, H 5.23, N 5.44.

[(Dipp$_2$Im)Cu(Me$_2$Im^{Me})]FAP (5c): 4 (70 mg, 39 μmol) and Me$_2$Im^{Me} (11 mg, 89 μmol) were dissolved in THF (3 mL) at room temperature. The solution was stirred for 1 h at room temperature. All volatiles were removed under reduced pressure and the residue was washed with hexane (2x 5 mL) and dried *in vacuo* yielding **5c** (57 mg, 55 μmol, 71 %) as a colorless solid. ^{1}H NMR (500.1 MHz, d_8-THF, 298°K): δ = 7.75 (s, 2H, NC*H*C*H*N), 7.56 (t, 2H, $^{3}J_{H-H}$ = 7.8 Hz, aryl-C$_{para}$*H*), 7.43 (d, 4H, $^{3}J_{H-H}$ = 7.8 Hz, aryl-C$_{meta}$*H*), 2.90 (s, 6H, NC*H*$_3$) 2.64 (sept, 4H, $^{3}J_{H-H}$ = 6.8 Hz, *i*Pr-C*H*), 1.96 (s, 6H, NCC*H*$_3$), 1.27 (d, 24H, $^{3}J_{H-H}$ = 6.8 Hz, *i*Pr-C*H*$_3$); ^{13}C{^{1}H} NMR (125.8 MHz, d_8-THF, 298°K): δ = 180.7 (Dipp$_2$Im-N*C*N), 172.8 (Me$_2$Im^{Me}-N*C*N), 146.8 (aryl-*C*$_{ortho}$), 135.4 (aryl-*C*$_{ipso}$), 131.4 (aryl-*C*$_{para}$), 126.8 (N*CC*N), 125.1 (N*C*H*C*HN), 124.9 (aryl-*C*$_{meta}$), 34.6 (N*C*H$_3$), 29.5 (*i*Pr-*C*H), 25.2 (*i*Pr-*C*H$_3$), 23.7 (*i*Pr-*C*H$_3$), 8.1 (NC*C*H$_3$); ^{19}F NMR (470.5 MHz, d_8-THF, 298°K): δ = −45.1 (dm, 1F, $^{1}J_{P-F}$ = 891 Hz, P*F*), −80.8 (m, 3F,

C*F*$_3$), −82.4 (m, 6F, C*F*$_3$), −88.0 (dm, 2F, $^1J_{P-F}$ = 905 Hz, P*F*$_2$), −116.5 (dm, 2F, $^2J_{P-F}$ = 82 Hz, C*F*$_2$), −117.1 (dm, 4F, $^2J_{P-F}$ = 97 Hz, C*F*$_2$); ^{31}P NMR (202.4 MHz, *d*$_8$-THF, 298°K): δ = −148.2 (tdm, $^1J_{P-F}$ = 905 Hz, $^1J_{P-F}$ = 891 Hz); IR ([cm^{-1}]): 3141 (vw), 2956 (w), 2927 (w), 2873 (w), 1648 (vw), 1594 (vw), 1574 (vw), 1549 (vw), 1470 (w), 1460 (w), 1435 (w), 1414 (w), 1388 (w), 1367 (w), 1347 (vw), 1310 (w), 1295 (m), 1217 (vs), 1185 (vs), 1142 (m), 1124 (s), 1090 (m), 1059 (m), 972 (m), 961 (m), 936 (w), 846 (w), 820 (s), 811 (s), 783 (w), 767 (m), 742 (m), 729 (s), 701 (w), 636 (w), 618 (vs), 581 (m), 533 (w), 505 (w), 495 (w), 439 (w), 429 (w); HRMS (ESI) *m*/*z* [*M*]$^+$ calcd. for $C_{34}H_{48}CuN_4$: 575.3175, found: 575.3146; *m/z* ***FAP***$^-$ calcd. for $C_6F_{18}P$: 444.9450, found: 444.9453; elemental analysis calcd. (%) for $C_{40}H_{48}CuF_{18}N_4P$: C 47.04, H 4.74, N, 5.20; found C 47.68, H 4.94, N 5.62.

[(Dipp$_2$Im)Cu(cAAC^{Me})]FAP (5d): 4 (116 mg, 64.7 µmol) and cAAC^{Me} (37.0 mg, 130 µmol) were dissolved in THF (3 mL) at room temperature. The solution was stirred for 30 min at room temperature. Hexane (15 mL) was added and a solid precipitated. The solid was isolated by filtration and dried *in vacuo* yielding **5d** (111 mg, 93.9 µmol, 73 %) as a colorless solid. ^{1}H NMR (500.1 MHz, *d*$_8$-THF, 298°K): δ = 7.60 (s, 2H, NC*H*C*H*N), 7.51 (t, 2H, $^3J_{H-H}$ = 7.8 Hz, Dipp$_2$Im-aryl-C$_{para}$*H*), 7.37 (t, 1H, $^3J_{H-H}$ = 7.8 Hz, cAAC^{Me}-aryl-C$_{para}$*H*), 7.33 (d, 4H, $^3J_{H-H}$ = 7.8 Hz, Dipp$_2$Im-aryl-C$_{meta}$*H*), 7.19 (d, 2H, $^3J_{H-H}$ = 7.8 Hz, cAAC^{Me}-aryl-C$_{meta}$*H*), 2.59 (sept, 2H, $^3J_{H-H}$ = 6.8 Hz, cAAC^{Me}-*i*Pr-C*H*), 2.51 (sept, 2H, $^3J_{H-H}$ = 6.8 Hz, Dipp$_2$Im-*i*Pr-C*H*), 1.89 (s, 2H, C*H*$_2$), 1.21 (s, 6H, NC(C*H*$_3$)$_2$), 1.19 (d, 6H, $^3J_{H-H}$ = 6.8 Hz, cAAC^{Me}-*i*Pr-C*H*$_3$), 1.13 (d, 12H, $^3J_{H-H}$ = 6.8 Hz, Dipp$_2$Im-*i*Pr-C*H*$_3$), 1.12 (d, 12H, $^3J_{H-H}$ = 6.8 Hz, Dipp$_2$Im-*i*Pr-C*H*$_3$), 0.86 (s, 6H, CC(C*H*$_3$)$_2$), 0.70 (d, 6H, $^3J_{H-H}$ = 6.8 Hz, cAAC^{Me}-*i*Pr-C*H*$_3$); ^{13}C{^{1}H} NMR (125.8 MHz, *d*$_8$-THF, 298°K): δ = 248.7 (cAAC^{Me}-*C*Cu), 178.8 (Dipp$_2$Im-*C*Cu), 146.2 (Dipp$_2$Im-aryl-*C*$_{ortho}$), 145.3 (cAAC^{Me}-aryl-*C*$_{ortho}$), 135.7 (Dipp$_2$Im-aryl-*C*$_{ipso}$), 134.4 (cAAC^{Me}-aryl-*C*$_{ipso}$), 131.5 (Dipp$_2$Im-aryl-*C*$_{para}$), 130.6 (cAAC^{Me}-aryl-*C*$_{para}$), 126.4 (N*C*H*C*HN), 125.5 (cAAC^{Me}-aryl-*C*$_{meta}$), 125.0 (Dipp$_2$Im-aryl-*C*$_{meta}$), 84.3 (N*C*(CH$_3$)$_2$), 55.5 (C*C*(CH$_3$)$_2$), 49.1 (*C*H$_2$), 29.44 (cAAC^{Me}-*i*Pr-*C*H), 29.38 (Dipp$_2$Im-*i*Pr-*C*H), 29.0 (NC(*C*H$_3$)$_2$), 27.9 (CC(*C*H$_3$)$_2$), 27.8 (cAAC^{Me}-*i*Pr-*C*H$_3$), 24.8 (Dipp$_2$Im-*i*Pr-*C*H$_3$), 24.1 (Dipp$_2$Im-*i*Pr-*C*H$_3$), 22.2 (cAAC^{Me}-*i*Pr-*C*H$_3$); ^{19}F NMR (470.6 MHz, *d*$_8$-THF, 298°K): δ = −45.1 (dm, 1F, $^1J_{P-F}$ = 890 Hz, P*F*), −80.7 (m, 3F, C*F*$_3$), −82.4 (m, 6F, C*F*$_3$), −88.0 (dm, 2F, $^1J_{P-F}$ = 905 Hz, P*F*$_2$), −116.4 (dm, 2F, $^2J_{P-F}$ = 82 Hz, C*F*$_2$), −117.0 (dm, 4F, $^2J_{P-F}$ = 96 Hz, C*F*$_2$); ^{31}P NMR (202.4 MHz, *d*$_8$-THF, 298°K): δ = −148.3 (tdm, $^1J_{P-F}$ = 905 Hz, $^1J_{P-F}$ = 890 Hz); IR ([cm^{-1}]): 3180

(vw), 2965 (w), 2873 (w), 1592 (vw), 1524 (vw), 1461 (w), 1405 (vw), 1388 (vw), 1366 (vw), 1311 (w), 1295 (w), 1212 (vs), 1178 (s), 1135 (m), 1126 (m), 1098 (m), 1070 (w), 960 (w), 815 (m), 807 (m), 779 (w), 760 (s), 723 (s), 701 (w), 636 (w), 618 (vs), 581 (w), 550 (vw), 533 (w), 494 (w), 438 (w), 429 (w); HRMS (ESI) *m*/*z* [*M*]$^+$ calcd. for $C_{47}H_{67}CuN_3$: 736.4631, found: 736.4609; *m/z* ***FAP***$^-$ calcd. for $C_6F_{18}P$: 444.9450, found: 444.9439; elemental analysis calcd. (%) for $C_{53}H_{67}CuF_{18}N_3P$: C 53.85, H 5.71, N, 3.55; found C 54.16, H 5.48, N 3.61.

[(Dipp$_2$Im)Cu(PPh$_3$)]FAP (5e): 4 (106 mg, 59.1 µmol) and PPh_3 (31.0 mg, 118 µmol) were dissolved in EtOH (2 mL) at room temperature. The suspension was stirred for 15 min at room temperature, filtered and the filtrate stored at −30 °C. Overnight, a colorless solid precipitated. The supernatant solution was decanted off and the remaining solid was dried *in vacuo* yielding **5e** (65.0 mg, 56.1 µmol, 47 %) as a colorless solid. ^{1}H NMR (500.1 MHz, *d*$_8$-THF, 298°K): δ = 7.81 (s, 2H, NC*H*C*H*N), 7.65 (t, 2H, $^3J_{H\text{-}H}$ = 7.7 Hz, Dipp$_2$Im-C$_{meta}$*H*), 7.49 (t, 3H, $^3J_{H\text{-}H}$ = 7.6 Hz, PPh$_3$-C$_{para}$*H*), 7.46 (d, 4H, $^3J_{H\text{-}H}$ = 7.7 Hz, Dipp$_2$Im-C$_{meta}$*H*), 7.35 (t, 6H, $^3J_{H\text{-}H}$ = 7.6 Hz, PPh$_3$-C$_{meta}$*H*), 6.97 (br, 6H, aryl-PPh$_3$-C$_{ortho}$*H*), 2.63 (sept, 4H, $^3J_{H\text{-}H}$ = 6.9 Hz, *i*Pr-C*H*), 1.26 (d, 12H, $^3J_{H\text{-}H}$ = 6.9 Hz, *i*Pr-C*H*$_3$), 1.15 (d, 12H, $^3J_{H\text{-}H}$ = 6.9 Hz, *i*Pr-C*H*$_3$); ^{13}C{^{1}H} NMR (125.8 MHz, *d*$_8$-THF, 298°K): δ = 178.0 (N*C*N), 146.6 (Dipp$_2$Im-*C*$_{ortho}$), 135.1 (Dipp$_2$Im-*C*$_{ipso}$), 134.1 (PPh$_3$-*C*$_{ortho}$), 132.5 (PPh$_3$-*C*$_{para}$), 131.7 (Dipp$_2$Im-*C*$_{para}$), 130.2 (PPh$_3$-*C*$_{meta}$), 125.7 (N*C*H*C*HN), 125.2 (Dipp$_2$Im-*C*$_{meta}$), 29.6 (*i*Pr-*C*H), 25.3 (*i*Pr-*C*H$_3$), 23.6 (*i*Pr-*C*H$_3$). The resonance of the PPh$_3$ *ipso* carbon atom was not observed; ^{19}F NMR (470.6 MHz, *d*$_8$-THF, 298°K): δ = −45.1 (dm, 1F, $^1J_{P\text{-}F}$ = 891 Hz, P*F*), −80.7 (m, 3F, C*F*$_3$), −82.4 (m, 6F, C*F*$_3$), −88.0 (dm, 2F, $^1J_{P\text{-}F}$ = 905 Hz, P*F*$_2$), −116.4 (dm, 2F, $^2J_{P\text{-}F}$ = 82 Hz, C*F*$_2$), −117.0 (dm, 4F, $^2J_{P\text{-}F}$ = 98 Hz, C*F*$_2$); ^{31}P NMR (202.4 MHz, *d*$_8$-THF, 298°K): δ = 8.3 (s, *P*Ph$_3$), −148.2 (tdm, $^1J_{P\text{-}F}$ = 905 Hz, $^1J_{P\text{-}F}$ = 891 Hz, *P*F$_2$); IR ([cm^{-1}]): 3143 (vw), 3076 (vw), 2967 (w), 2929 (w), 2874 (vw), 1718 (vw), 1591 (vw), 1545 (vw), 1460 (w), 1438 (w), 1414 w), 1388 (vw), 1367 (vw), 1296 (m), 1215 (vs), 1181 (s), 1137 (s), 1125 (s), 1098 (s), 1061 (m), 1027 (vw), 998 (vw), 961 (m), 937 (w), 848 (vw), 811 (s), 760 (m), 743 (m), 721 (m), 693 (s), 618 (w), 580 (vs), 532 (w), 507 (m), 494 (m), 439 (m), 430 (w), 407 (vw); HRMS (ESI) *m*/*z* [*M*+O]$^+$ calcd. for $C_{45}H_{51}CuN_2OP$: 736.4631, found: 736.4609; *m/z* ***FAP***$^-$ calcd. for $C_6F_{18}P$: 444.9450, found: 444.9446; elemental analysis calcd. (%) for $C_{51}H_{51}CuF_{18}N_2P_2$: C 52.83, H 4.43, N, 2.42; found C 53.09, H 4.42, N 2.59.

[(Dipp$_2$Im)Cu(C$_6$Me$_6$)]FAP (**5f**): The phosphorane $(C_2F_5)_3PF_2$ (164 µL, 697 µmol) was added at room temperature to a solution of **3** (328 mg, 696 µmol) and C_6Me_6 (113 mg, 696 µmol) in CH_2Cl_2 (5 mL). The brownish solution was stirred for 2 h at room temperature. The solution was filtered through a plug of Celite and all volatiles were removed under reduced pressure. The remaining solid was suspended in hexane (10 mL), isolated by filtration and washed with hexane (5 mL). The solid was dried *in vacuo* yielding **5f** (494 mg, 466 µmol, 67 %) as an off-white solid; ^{1}H NMR (500.1 MHz, CD_2Cl_2, 298°K): δ = 7.61 (t, 2H, $^{3}J_{H-H}$ = 7.8 Hz, Dipp$_2$Im-C$_{meta}$*H*), 7.40 (d, 4H, $^{3}J_{H-H}$ = 7.8 Hz, C$_{meta}$*H*), 7.20 (s, 2H, NC*H*C*H*N), 2.32 (sept, 4H, $^{3}J_{H-H}$ = 6.9 Hz, *i*Pr-C*H*), 1.90 (s, 18H, C$_6$*Me*$_6$) 1.21 (d, 12H, $^{3}J_{H-H}$ = 6.9 Hz, *i*Pr-C*H*$_3$), 1.16 (d, 12H, $^{3}J_{H-H}$ = 6.9 Hz, *i*Pr-C*H*$_3$); $^{13}C\{^{1}H\}$ NMR (125.8 MHz, CD_2Cl_2, 298°K): δ = 177.8 (N*C*N), 145.8 (aryl-*C*$_{ortho}$), 135.0 (aryl-*C*$_{ipso}$), 131.3 (aryl-*C*$_{para}$), 129.8 (*C*$_6$Me$_6$), 124.8 (aryl-*C*$_{meta}$), 124.7 (N*C*H*C*HN), 29.2 (*i*Pr-*C*H), 24.4 (*i*Pr-*C*H$_3$), 24.0 (*i*Pr-*C*H$_3$), 17.5 (C$_6$*Me*$_6$); ^{19}F NMR (470.6 MHz, CD_2Cl_2, 298°K): δ = −45.2 (dm, 1F, $^{1}J_{P-F}$ = 891 Hz, P*F*), −80.6 (m, 3F, C*F*$_3$), −82.3 (m, 6F, C*F*$_3$), −88.6 (dm, 2F, $^{1}J_{P-F}$ = 905 Hz, P*F*$_2$), −116.2 (dm, 2F, $^{2}J_{P-F}$ = 83 Hz, C*F*$_2$), −116.7 (dm, 4F, $^{2}J_{P-F}$ = 98 Hz, C*F*$_2$); ^{31}P NMR (202.4 MHz, CD_2Cl_2, 298°K): δ = −147.6 (tdm, $^{1}J_{P-F}$ = 905 Hz, $^{1}J_{P-F}$ = 891 Hz); IR ([cm^{-1}]): 2967 (w), 2876 (vw), 1666 (vw), 1592 (vw), 1557 (vw), 1462 (w), 1410 (vw), 1388 (vw), 1366 (vw), 1312 (w), 1295 (w), 1213 (vs), 1179 (vs), 1144 (m), 1127 (m), 1097 (m), 1062 (w), 971 (w), 809 (s), 783 (vw), 763 (s), 744 (w), 725 (s), 698 (w), 637 (w), 618 (vs), 581 (w), 533 (w), 506 (vw), 495 (w), 439 (w), 429 (w); HRMS (ESI) *m/z* [*M*+O]$^{+}$ calcd. for $C_{39}H_{54}CuN_2$: 613.3583, found: 613.3570; *m/z* ***FAP***$^{-}$ calcd. for $C_6F_{18}P$: 444.9450, found: 444.9445; elemental analysis calcd. (%) for $C_{45}H_{54}CuF_{18}N_2P$: C 51.02, H 5.14, N, 2.64; found C 50.60, H 4.90, N 2.70.

[(F)(Cp)$_2$Ti(*µ*-F)Ti(Cp)$_2$(F)]FAP (7): The phosphorane $(C_2F_5)_3PF_2$ (100 µL, 425 µmol) was added at room temperature to a solution of **6** (170 mg, 787 µmol) in CH_2Cl_2 (30 mL). Upon addition the color of the solution changed from yellow to orange. The solution was stirred for 15 min at room temperature and an orange solid precipitated. All volatiles were removed under reduced pressure and the remaining solid was dried *in vacuo* yielding **7** (310 mg, 361 µmol, 92 %) as an orange solid. ^{1}H NMR (500.1 MHz, CD_2Cl_2, 298°K): δ = 6.71 (s); $^{13}C\{^{1}H\}$ NMR (125.8 MHz, CD_2Cl_2, 298°K): δ = 122.7; ^{19}F NMR (470.6 MHz, CD_2Cl_2, 298°K): δ = 168.2 (s, 2F, terminal-*F*), −44.9 (dm, 1F, $^{1}J_{P-F}$ = 891 Hz, P*F*), −80.5 (m, 3F, C*F*$_3$), −82.1 (m, 6F, C*F*$_3$), −88.4 (dm, 2F, $^{1}J_{P-F}$ = 900 Hz,

PF_2), −116.0 (dm, 2F, $^2J_{P\text{-}F}$ = 83 Hz, CF_2), −116.4 (dm, 4F, $^2J_{P\text{-}F}$ = 98 Hz, CF_2), −121.9 (s, 1F, *μ-F*); ^{31}P NMR (202.4 MHz, CD_2Cl_2, 298°K): δ = −147.2 (tdm, $^1J_{P\text{-}F}$ = 900 Hz, $^1J_{P\text{-}F}$ = 891 Hz); IR ([cm^{-1}]): 3130 (w), 1450 (w), 1436 (w), 1371 (vw), 1314 (w), 1298 (w), 1211 (s), 1175 (vs), 1126 (s), 1099 (s), 1073 (w), 1028 (w), 1013 (m), 971 (m), 875 (w), 834 (m), 817 (vs), 804 (vs), 763 (m), 743 (w), 724 (s), 637 (m), 616 (vs), 582 (vs), 533 (m), 480 (s), 426 (s); HRMS (ESI) *m/z* [*M*]$^+$ calcd. for $C_{20}H_{20}F_3Ti_2$: 413.0476, found: 413.0474; *m/z* ***FAP***$^-$ calcd. for $C_6F_{18}P$: 444.9450, found: 444.9449; elemental analysis calcd. (%) for $C_{26}H_{20}F_{21}PTi$: C 36.39, H 2.35; found C 36.79, H 2.32.

2) NMR Spectra of the Compounds

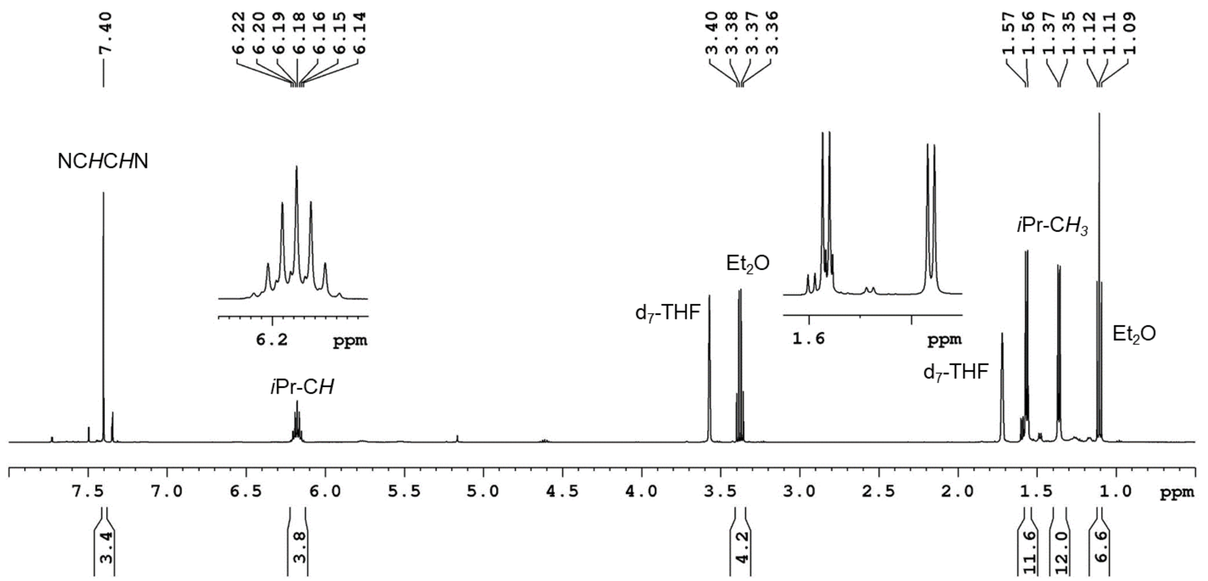

Figure S1: ^{1}H NMR spectrum (500.1 MHz) upon dissolution of *trans*-[Ni(*i*Pr$_2$Im)$_2$(OEt$_2$)(C$_6$F$_5$)]**FAP** (**2a[OEt$_2$]**) in *d*$_8$-THF.

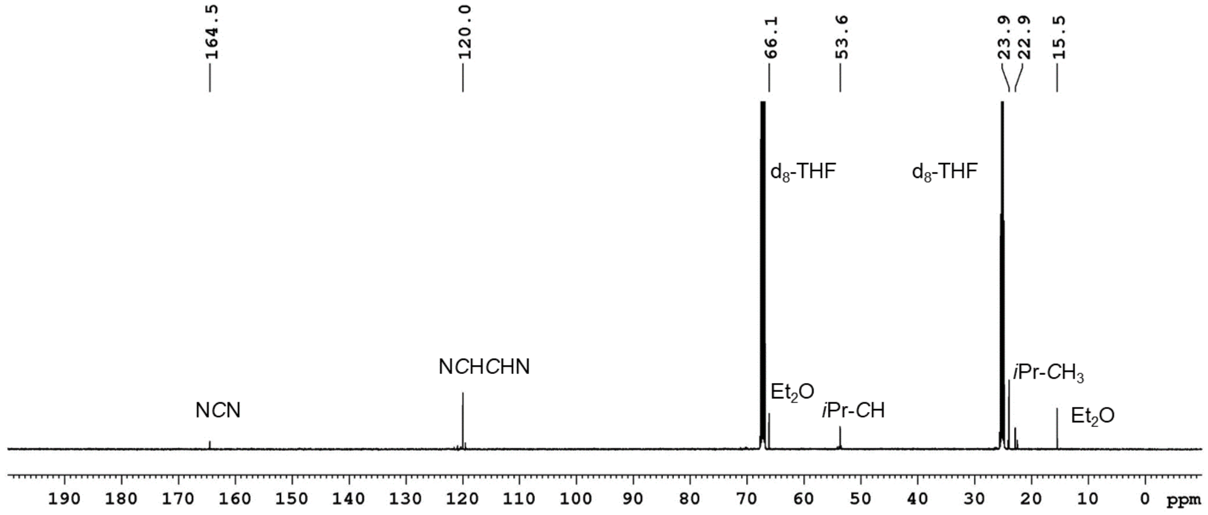

Figure S2: $^{13}C\{^{1}H\}$ NMR spectrum (125.8 MHz) upon dissolution of *trans*-[Ni(*i*Pr$_2$Im)$_2$(OEt$_2$)(C$_6$F$_5$)]**FAP** (**2a[OEt$_2$]**) in *d*$_8$-THF.

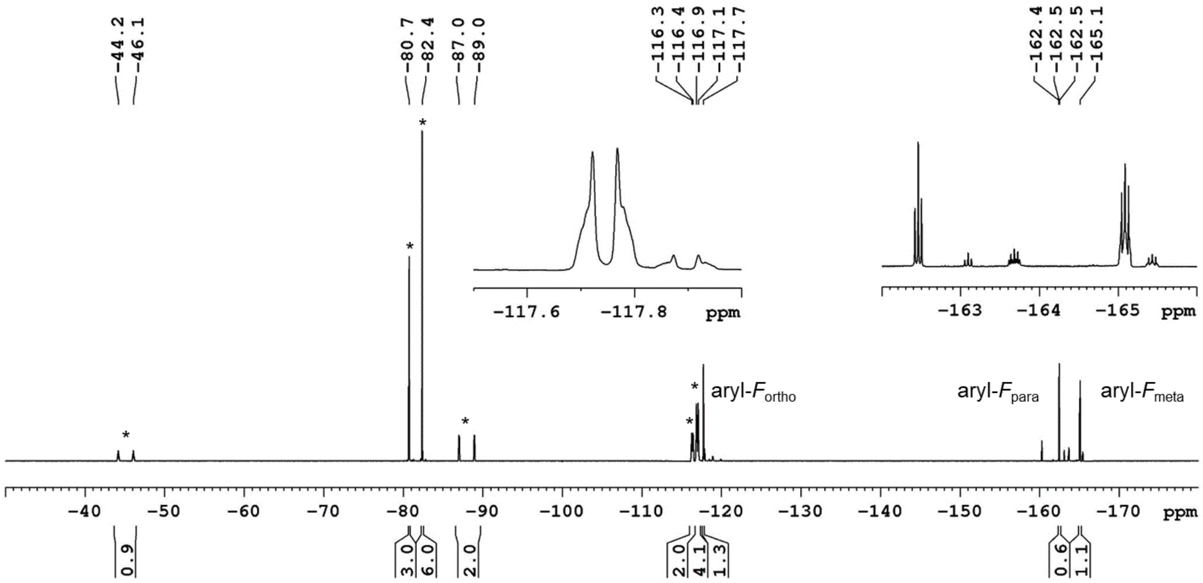

Figure S3: ^{19}F NMR spectrum (470.6 MHz) upon dissolution of *trans*-[Ni(iPr$_2$Im)$_2$(OEt$_2$)(C$_6$F$_5$)]**FAP** (**2a[OEt$_2$]**) in d_8-THF; the asterisk (*) indicates the anion.

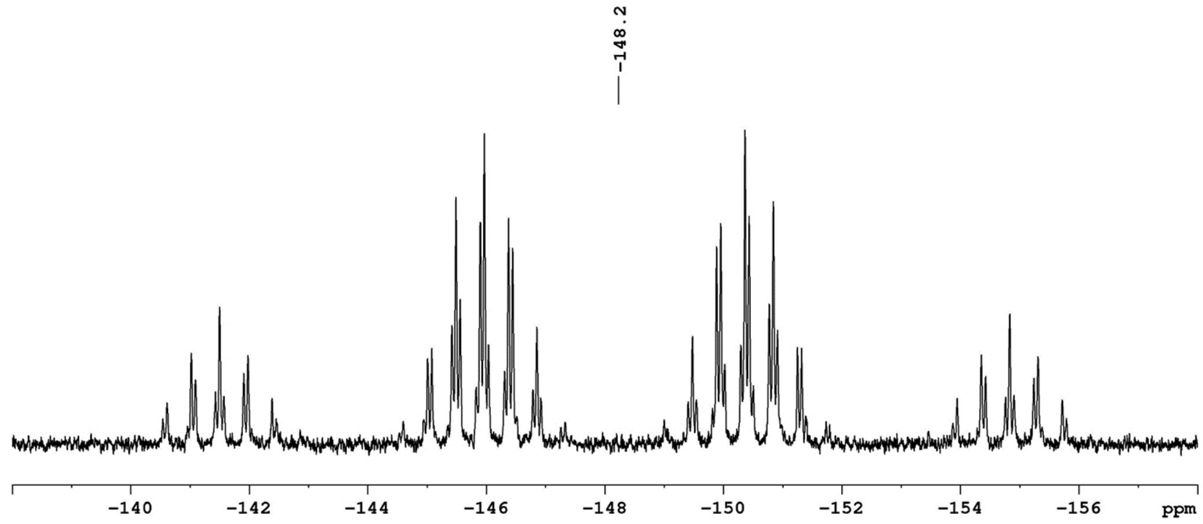

Figure S4: ^{31}P NMR spectrum (202.4 MHz) upon dissolution of *trans*-[Ni(iPr$_2$Im)$_2$(OEt$_2$)(C$_6$F$_5$)]**FAP** (**2a[OEt$_2$]**) in d_8-THF.

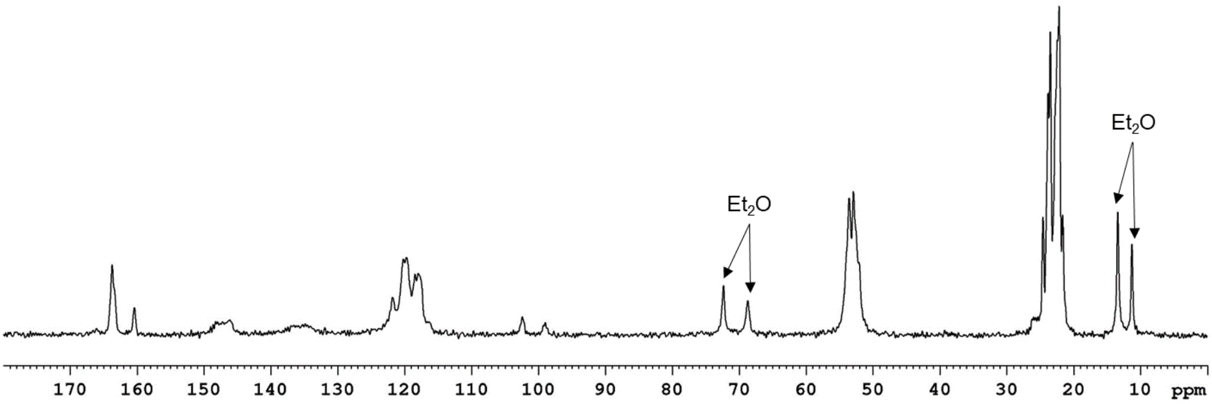

Figure S5: $^{13}C\{^1H\}$ CP/MAS NMR spectrum (100.6 MHz) of *trans*-[Ni(*i*Pr$_2$Im)$_2$(OEt$_2$)(C$_6$F$_5$)]**FAP** (**2a[OEt$_2$]**) (mass spinning rate 11.000 Hz).

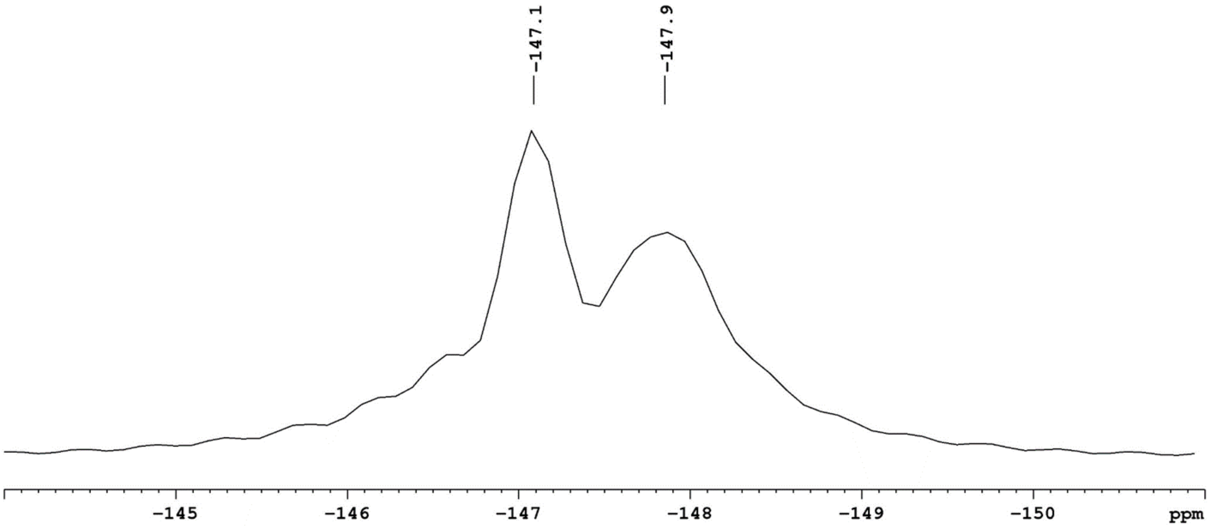

Figure S6: $^{31}P\{^{19}F\}$ MAS NMR spectrum (162.0 MHz) of *trans*-[Ni(*i*Pr$_2$Im)$_2$(OEt$_2$)(C$_6$F$_5$)]**FAP** (**2a[OEt$_2$]**) (mass spinning rate 14.500 Hz)..

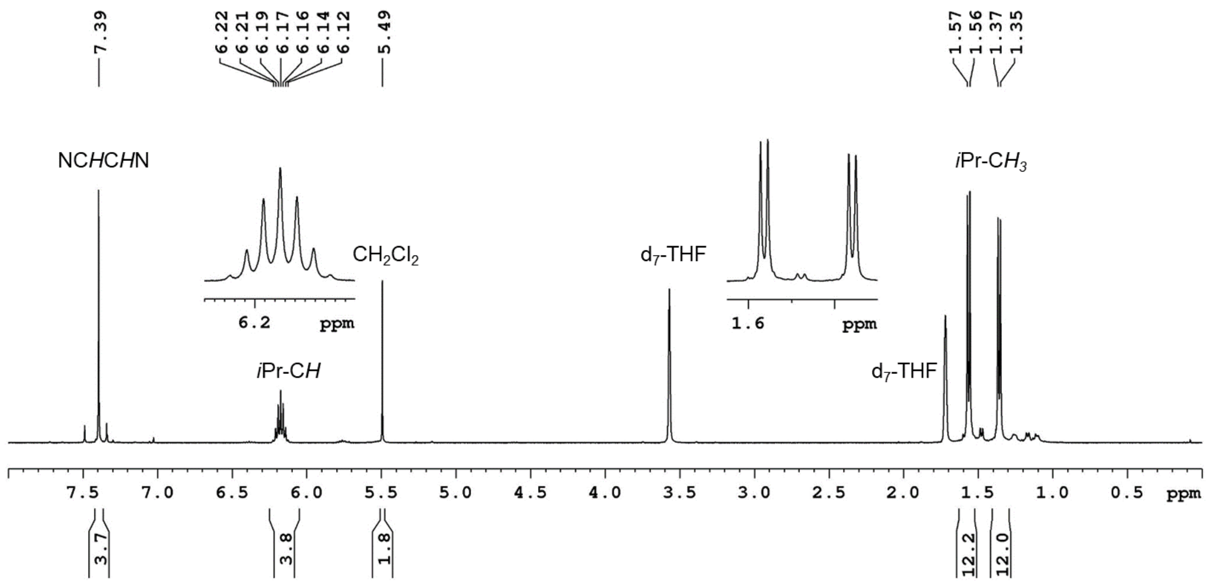

Figure S7: ^{1}H NMR spectrum (500.1 MHz) upon dissolution of *trans*-[Ni(*i*$Pr_2Im)_2(ClCH_2Cl)(C_6F_5)$]**FAP** (**2a[ClCH$_2$Cl]**) in *d*$_8$-THF.

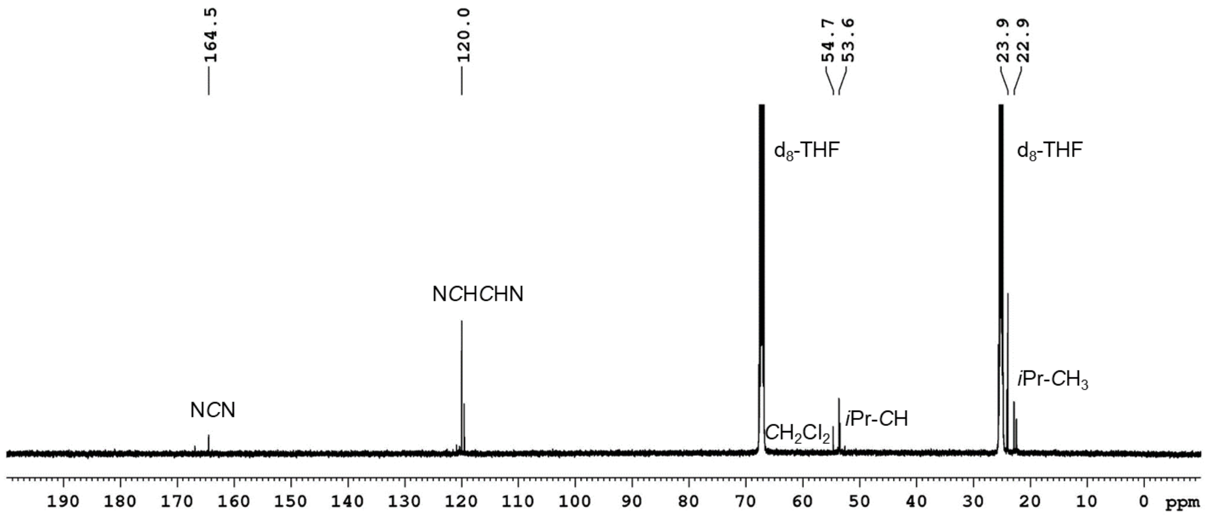

Figure S8: $^{13}C\{^{1}H\}$ NMR spectrum (125.8 MHz) upon dissolution of *trans*-[Ni(*i*$Pr_2Im)_2(ClCH_2Cl)(C_6F_5)$]**FAP** (**2a[ClCH$_2$Cl]**) in *d*$_8$-THF.

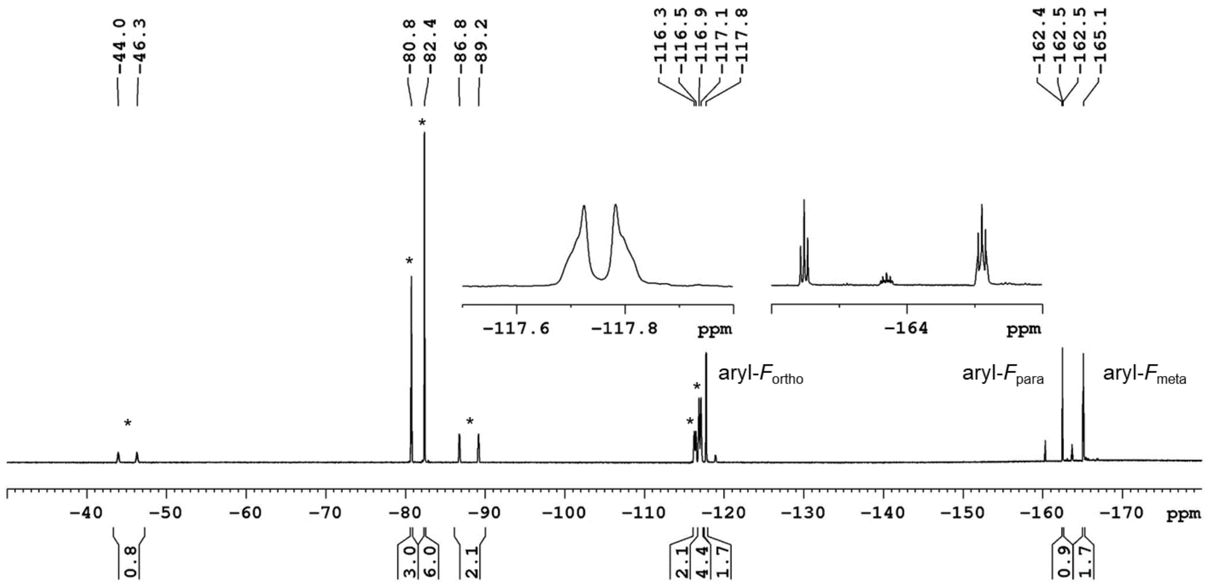

Figure S9: ^{19}F NMR spectrum (470.6 MHz) upon dissolution of *trans*-[Ni(*i*$Pr_2Im)_2(ClCH_2Cl)(C_6F_5)$]**FAP** (**2a[ClCH$_2$Cl]**) in d_8-THF; the asterisks (*) indicate resonances of the anion.

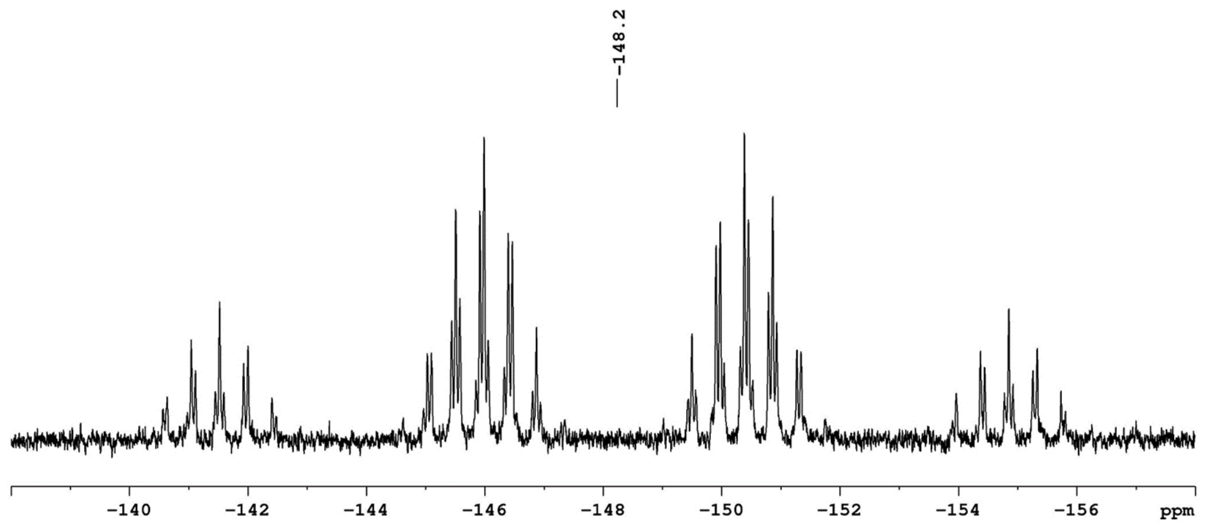

Figure S10: ^{31}P NMR spectrum (202.4 MHz) upon dissolution of *trans*-[Ni(*i*$Pr_2Im)_2(ClCH_2Cl)(C_6F_5)$]**FAP** (**2a[ClCH$_2$Cl]**) in d_8-THF.

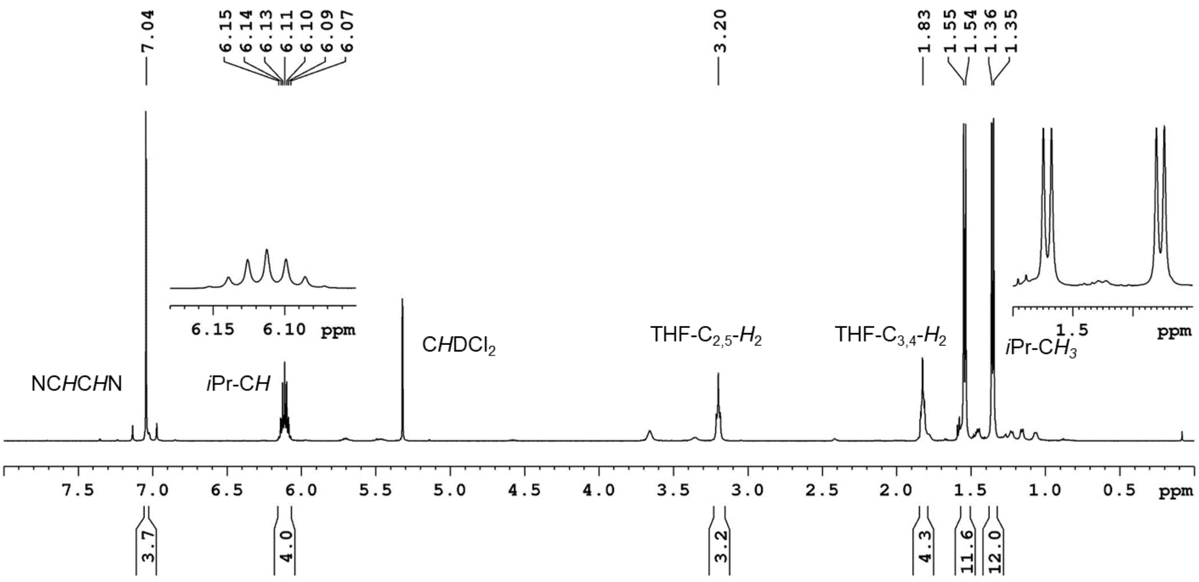

Figure S11: ^{1}H NMR spectrum (500.1 MHz) of *trans*-[Ni(*i*Pr$_2$Im)$_2$(thf)(C$_6$F$_5$)]**FAP** (**2a[thf]**) recorded in CD$_2$Cl$_2$.

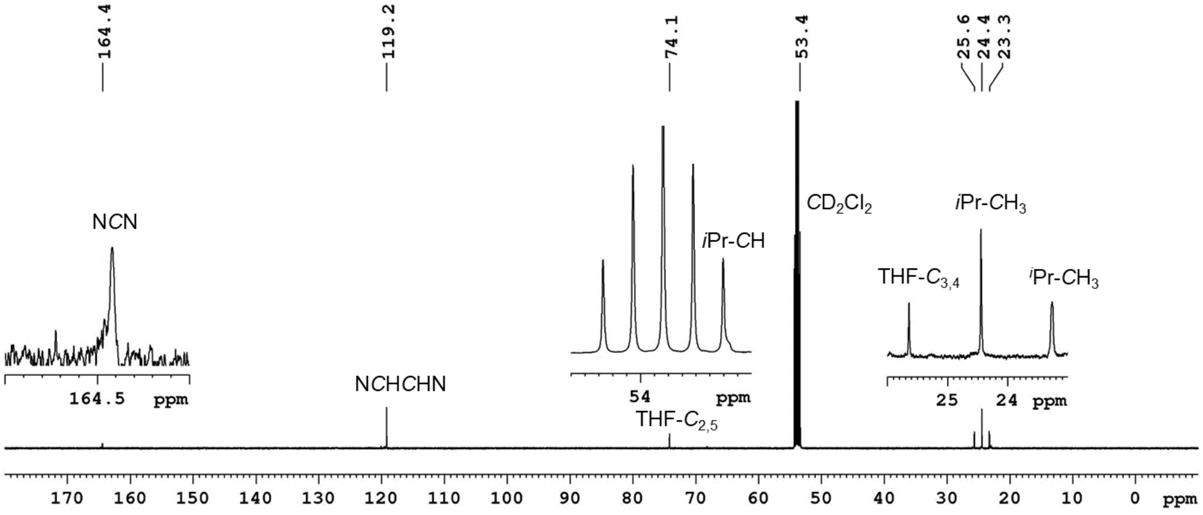

Figure S12: ^{13}C{^{1}H} NMR spectrum (125.8 MHz) of *trans*-[Ni(*i*Pr$_2$Im)$_2$(thf)(C$_6$F$_5$)]**FAP** (**2a[thf]**) recorded in CD$_2$Cl$_2$.

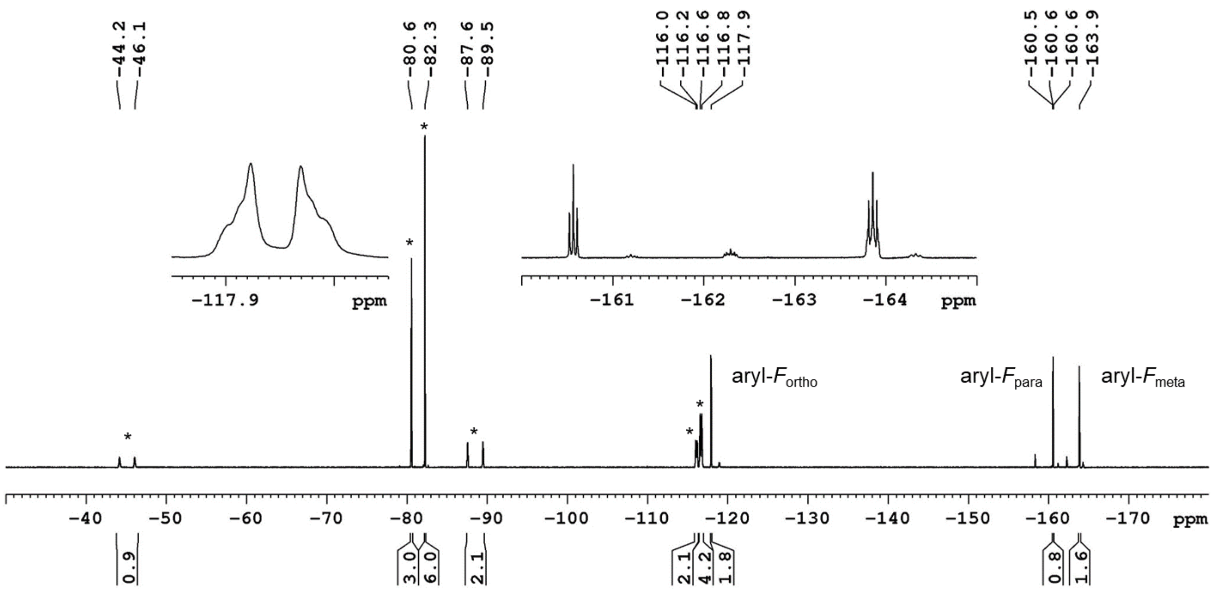

Figure S13: ^{19}F NMR spectrum (470.6 MHz) of *trans*-[Ni(*i*Pr_2Im)$_2$(thf)(C_6F_5)]**FAP** (**2a[thf]**) recorded in CD_2Cl_2; the asterisks (*) indicate resonances of the anion.

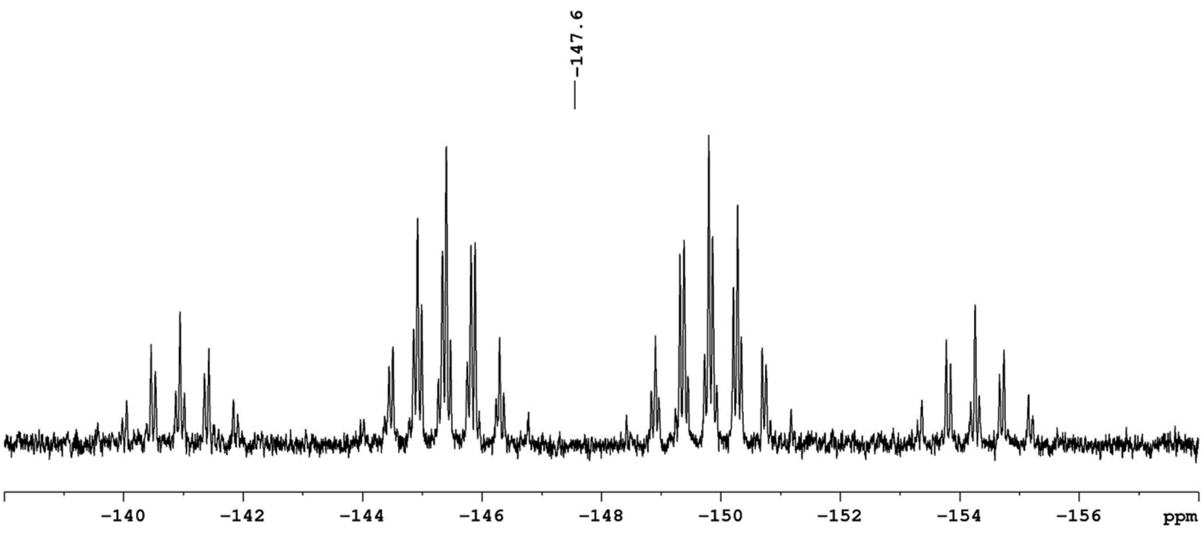

Figure S14: ^{31}P NMR spectrum (202.4 MHz) of *trans*-[Ni(*i*Pr_2Im)$_2$(thf)(C_6F_5)]**FAP** (**2a[thf]**) recorded in CD_2Cl_2.

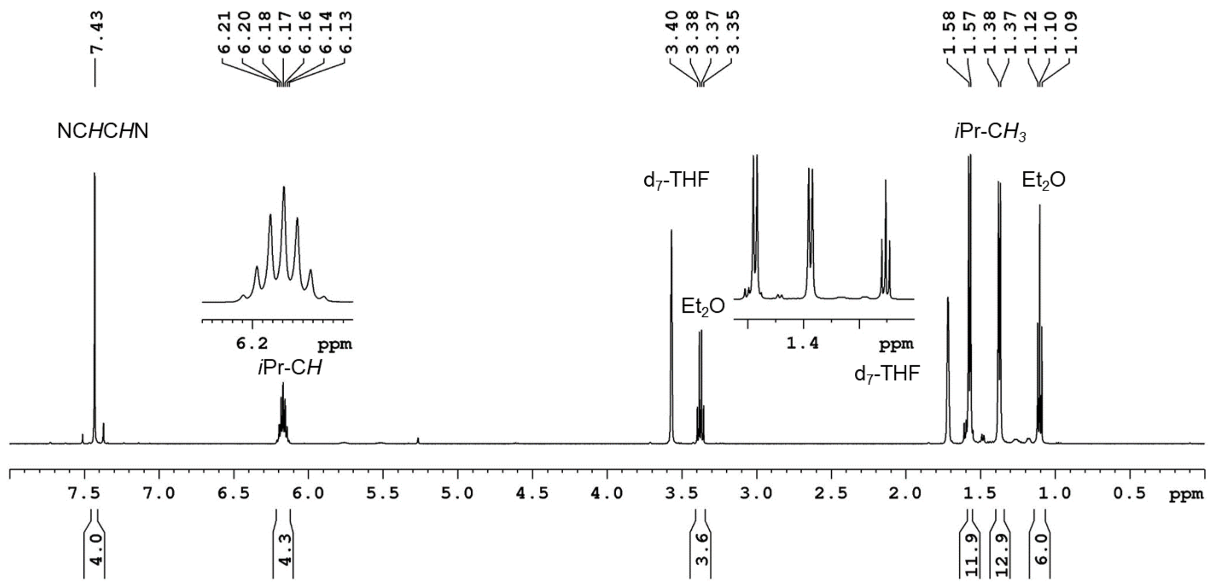

Figure S15: ^{1}H NMR spectrum (500.1 MHz) upon dissolution of *trans*-[Ni(*i*Pr$_2$Im)$_2$(OEt$_2$)(4-(CF$_3$)(C$_6$F$_4$))]**FAP** (**2b[OEt$_2$]**) in *d*$_8$-THF.

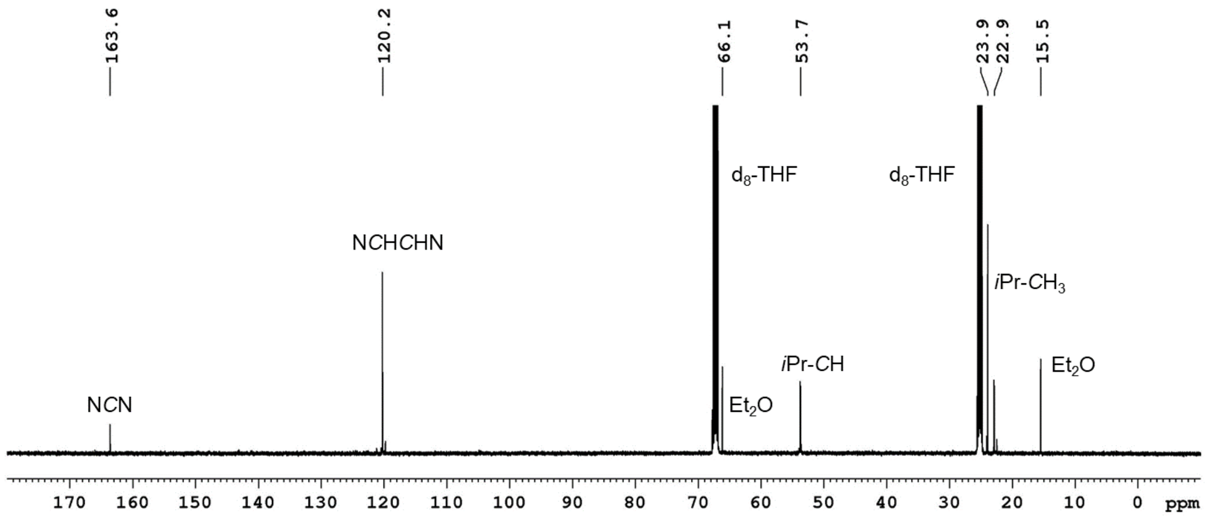

Figure S16: ^{13}C{^{1}H} NMR spectrum (125.8 MHz) upon dissolution of *trans*-[Ni(*i*Pr$_2$Im)$_2$(OEt$_2$)(4-(CF$_3$)(C$_6$F$_4$))]**FAP** (**2b[OEt$_2$]**) in *d*$_8$-THF.

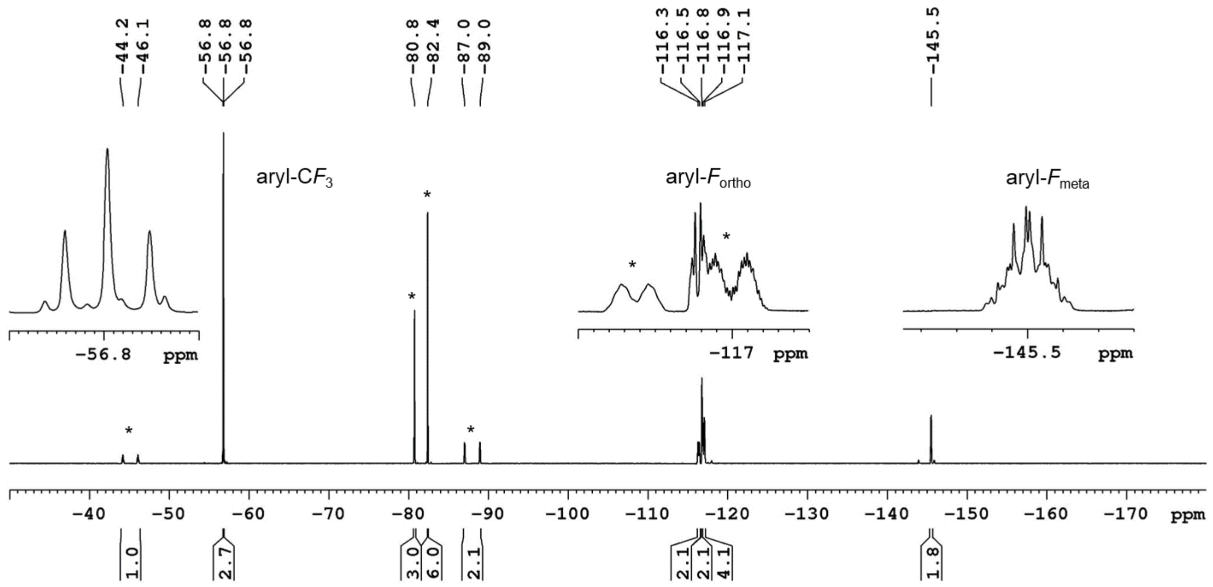

Figure S17: ^{19}F NMR spectrum (470.6 MHz) upon dissolution of *trans*-[Ni(*i*Pr_2Im)$_2$(OEt_2)(4-(CF_3)(C_6F_4))]**FAP** (**2b[OEt$_2$]**) in d_8-THF; the asterisks (*) indicate resonances of the anion.

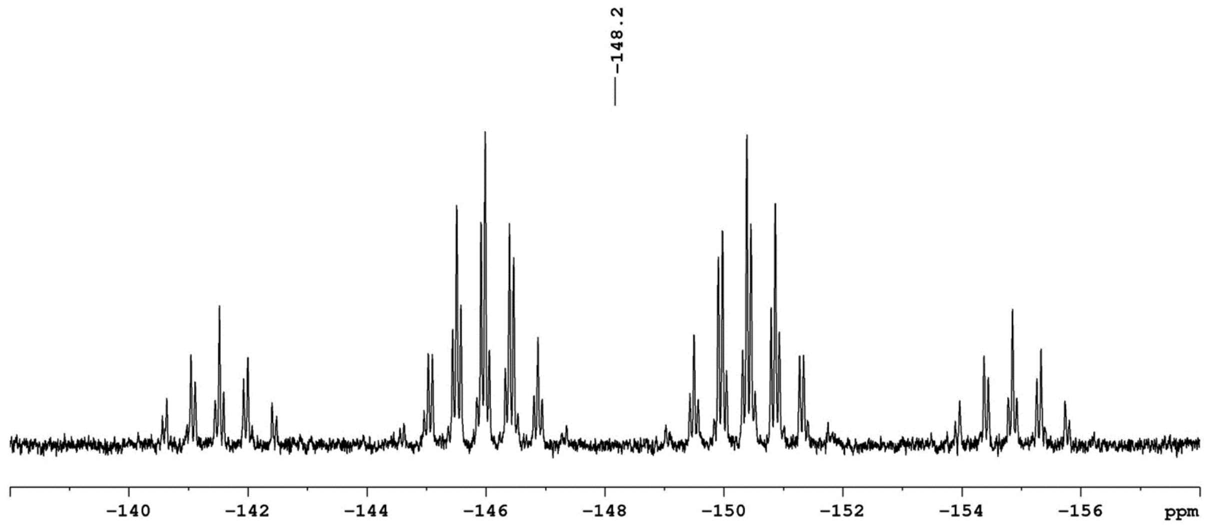

Figure S18: ^{31}P NMR spectrum (202.4 MHz) upon dissolution of *trans*-[Ni(*i*Pr_2Im)$_2$(OEt_2)(4-(CF_3)(C_6F_4))]**FAP** (**2b[OEt$_2$]**) in d_8-THF.

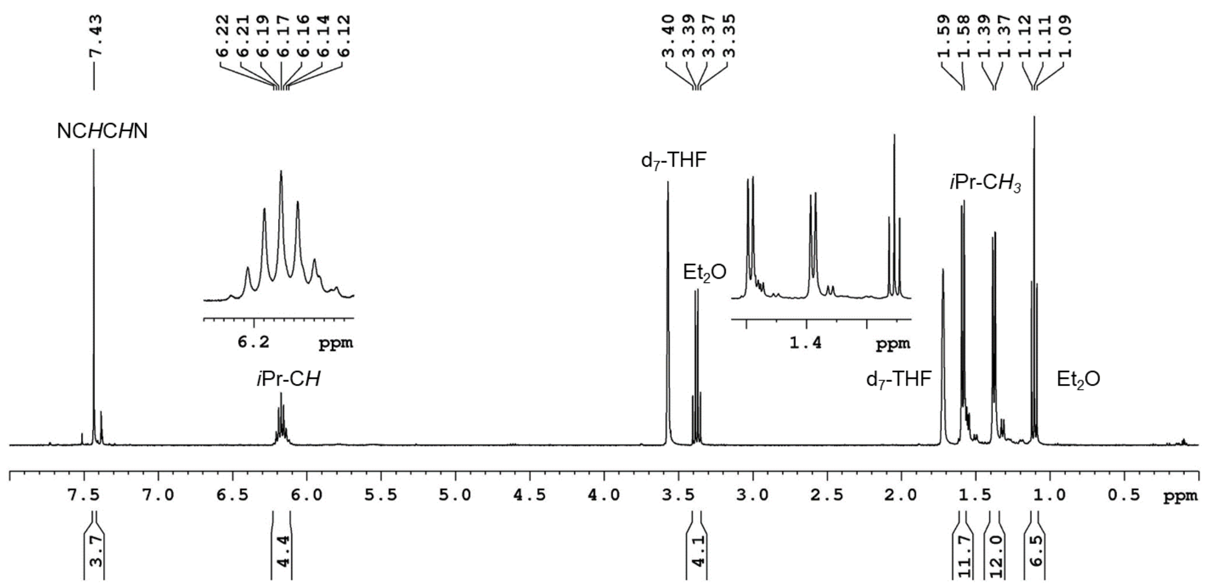

Figure S19: ^{1}H NMR spectrum (500.1 MHz) upon dissolution of *trans*-[Ni(*i*Pr$_2$Im)$_2$(OEt$_2$)(4-(C$_6$F$_5$)(C$_6$F$_4$))]**FAP** (**2c[OEt$_2$]**) in *d*$_8$-THF.

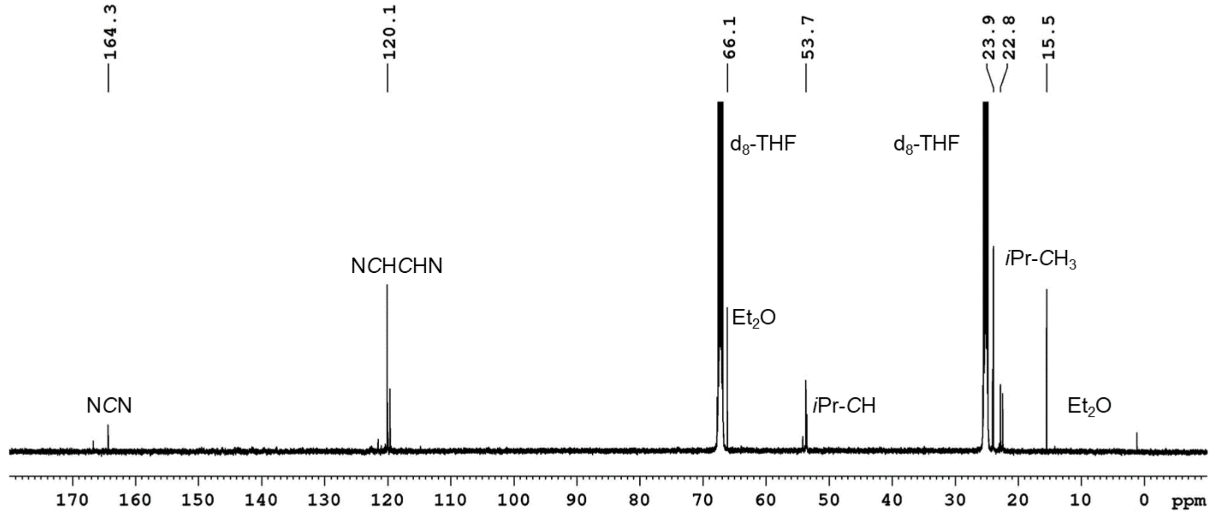

Figure S20: ^{13}C{^{1}H} NMR spectrum (125.8 MHz) upon dissolution of *trans*-[Ni(*i*Pr$_2$Im)$_2$(OEt$_2$)(4-(C$_6$F$_5$)(C$_6$F$_4$))]**FAP** (**2c[OEt$_2$]**) in *d*$_8$-THF.

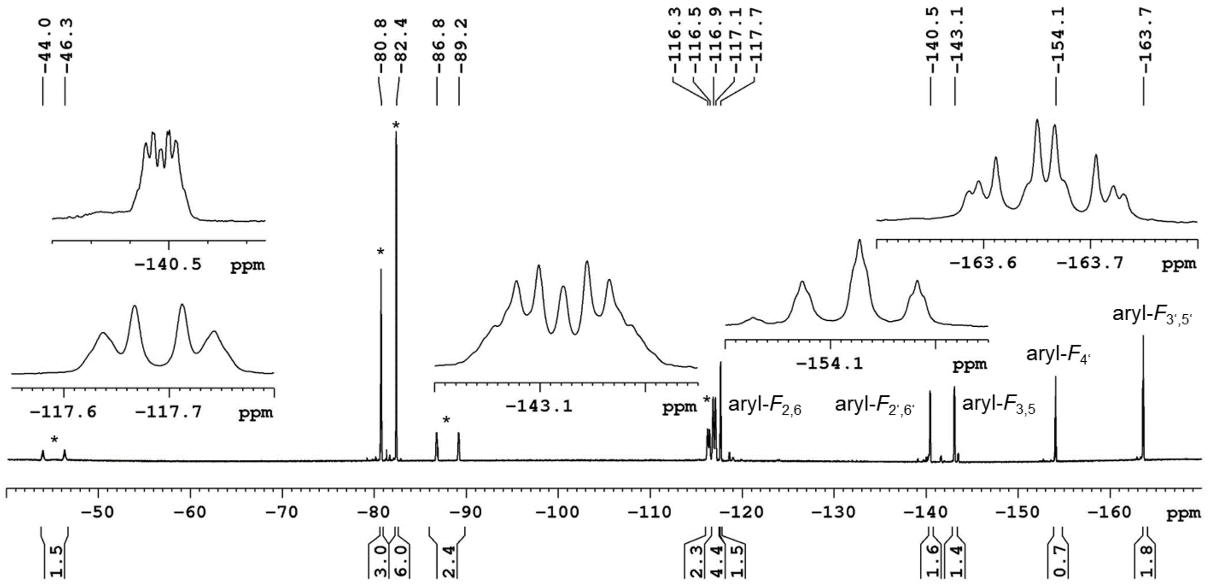

Figure S21: ^{19}F NMR spectrum (470.6 MHz) upon dissolution of *trans*-[Ni(*i*Pr_2Im)$_2$(OEt_2)(4-(C_6F_5)(C_6F_4))]**FAP** (**2c[OEt$_2$]**) in d_8-THF; the asteric (*) indicates the anion.

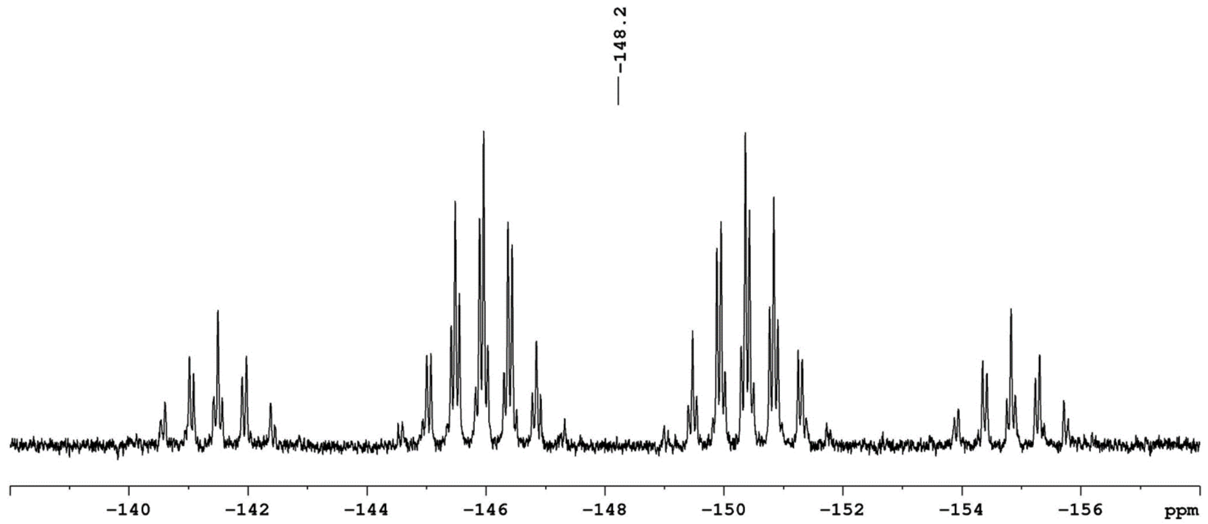

Figure S22: ^{31}P NMR spectrum (202.4 MHz) upon dissolution of *trans*-[Ni(*i*Pr_2Im)$_2$(OEt_2)(4-(C_6F_5)(C_6F_4))]**FAP** (**2c[OEt$_2$]**) in d_8-THF.

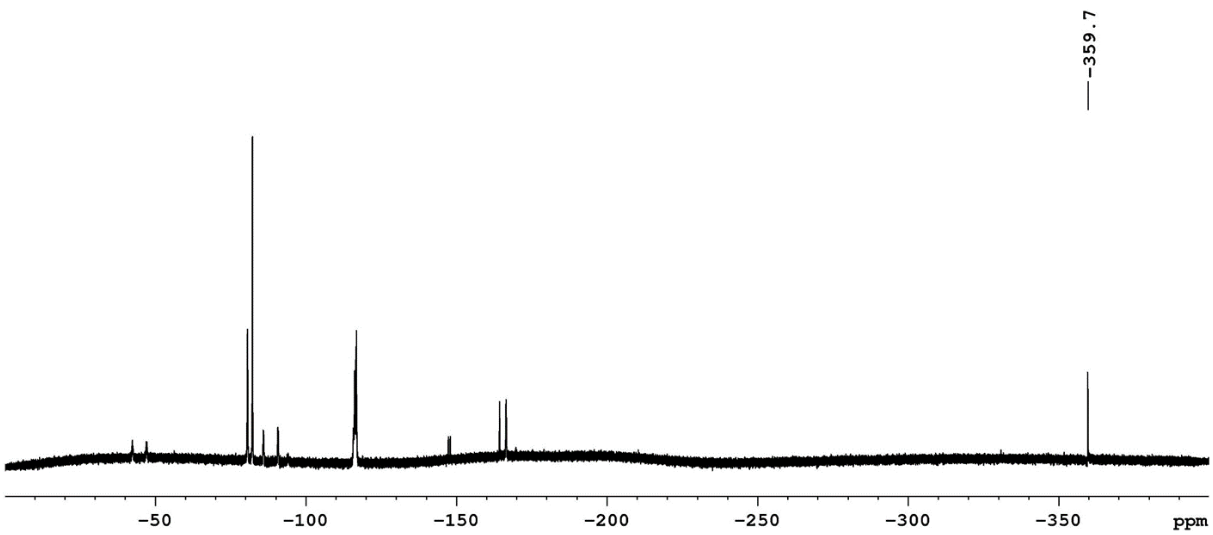

Figure S23: ^{19}F NMR spectrum (188.1 MHz) of the crude reaction mixture of **2a[ClCH$_2$Cl]** and [Me$_4$N]F in CH_2Cl_2. The resonance at –359.7 ppm indicates reformation of the Ni–F bond.

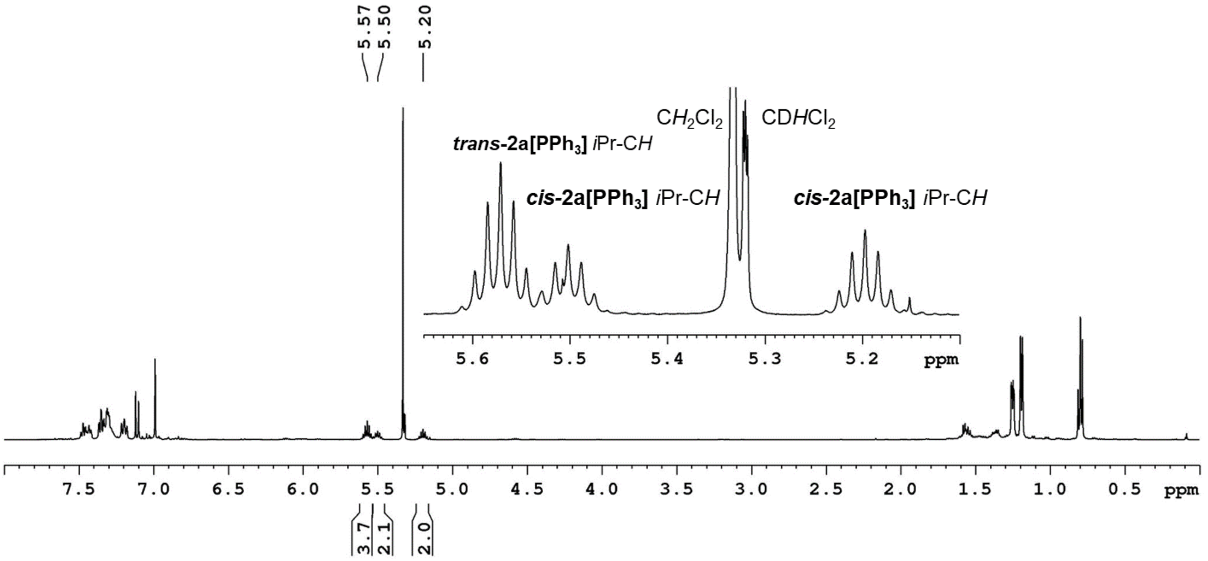

Figure S24: ^{1}H NMR spectrum (500.1 MHz) of the crude reaction mixture of **2a[ClCH$_2$Cl]** and PPh_3 recorded in CD_2Cl_2.

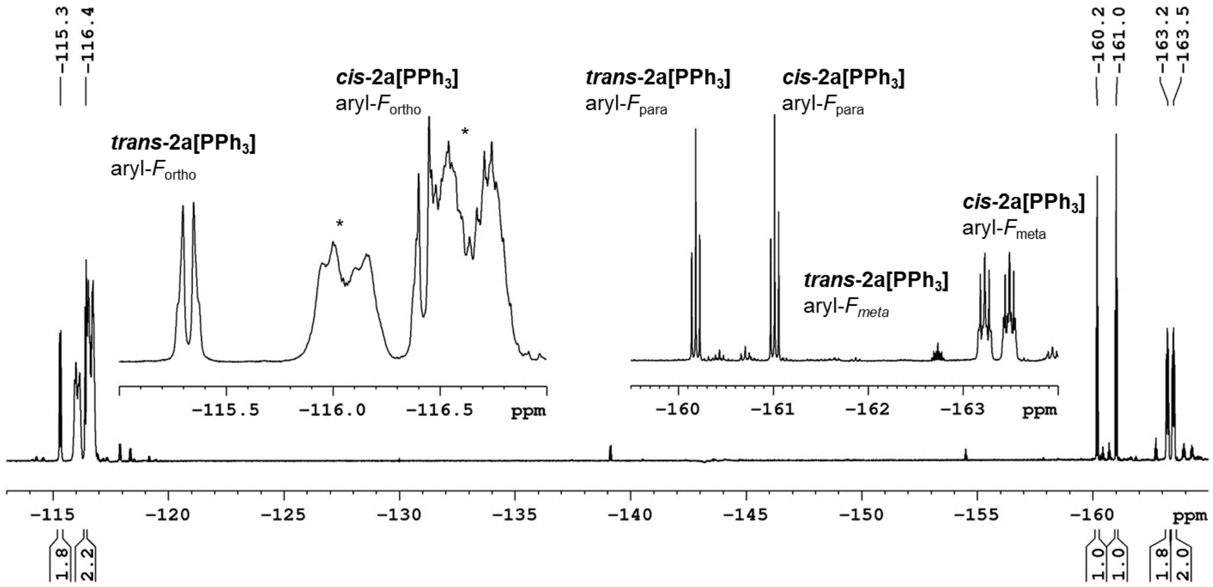

Figure S25: ^{19}F NMR spectrum (470.6 MHz) of the crude reaction mixture of **2a[ClCH$_2$Cl]** and PPh_3 recorded in CD_2Cl_2; the asteric (*) indicates the anion.

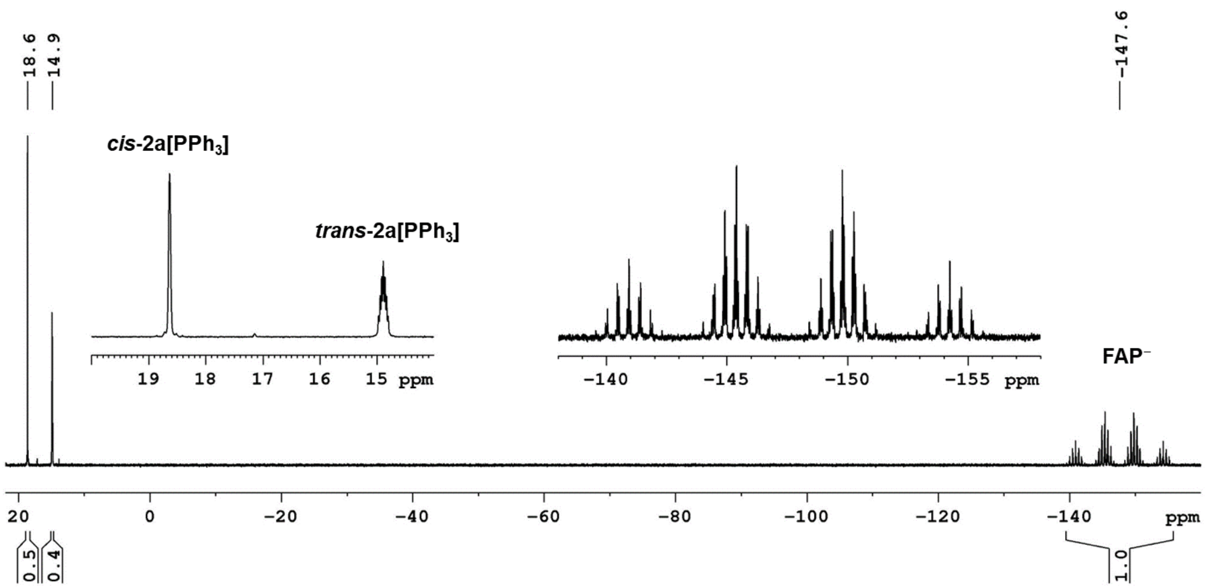

Figure S26: ^{31}P NMR spectrum (202.4 MHz) of the crude reaction mixture of **2a[ClCH$_2$Cl]** and PPh_3 recorded in CD_2Cl_2.

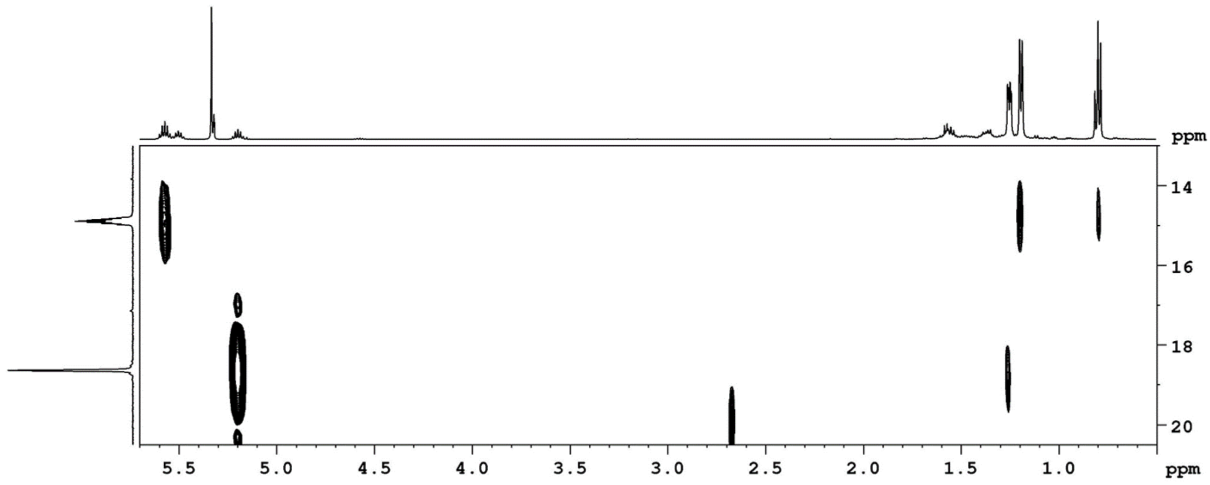

Figure S27: $^{31}P,^{1}H$ HMQC spectrum of the crude reaction mixture of **2a[ClCH$_2$Cl]** and PPh_3 recorded in CD_2Cl_2.

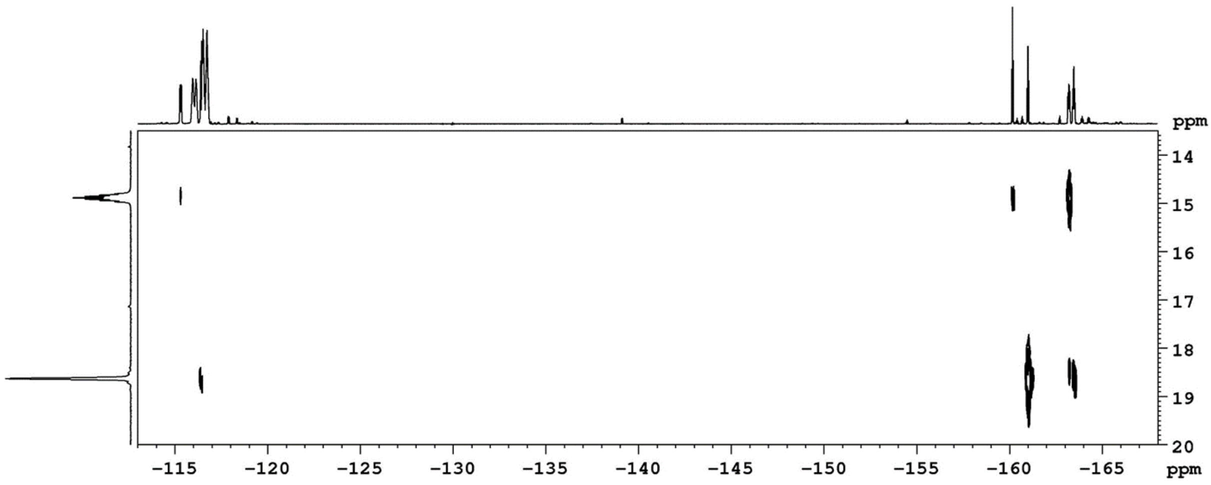

Figure S28: $^{31}P,^{19}F$ HMQC spectrum of the crude reaction mixture of **2a[ClCH$_2$Cl]** and PPh_3 recorded in CD_2Cl_2.

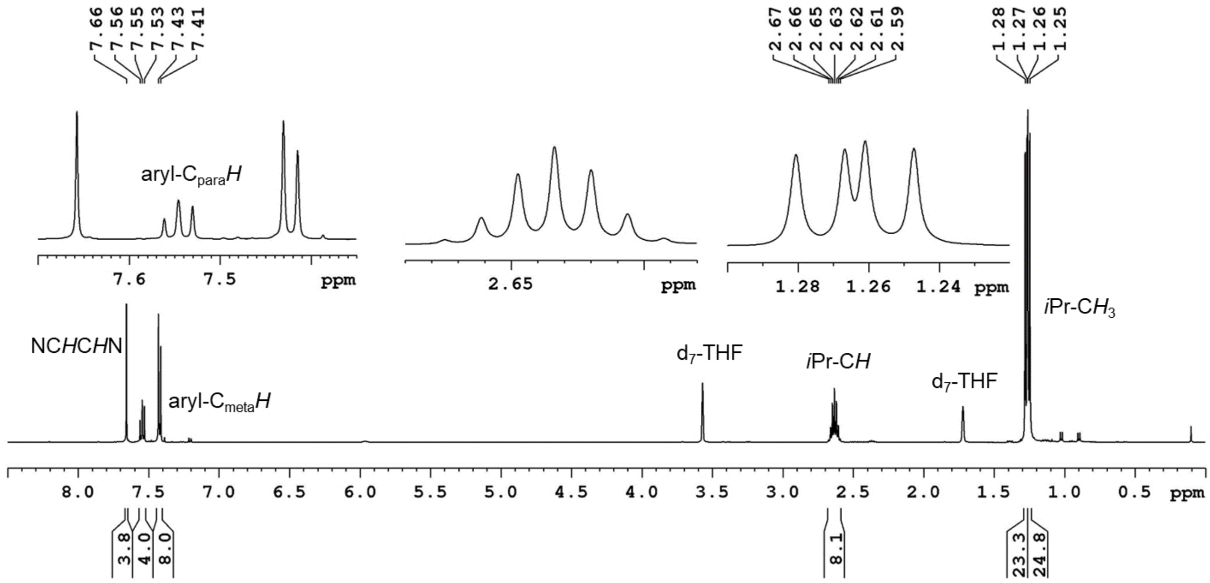

Figure S29: ^1H NMR spectrum (500.1 MHz) upon dissolution of [{(Dipp$_2$Im)Cu}$_2$]**FAP**$_2$ (**4**) in *d*$_8$-THF.

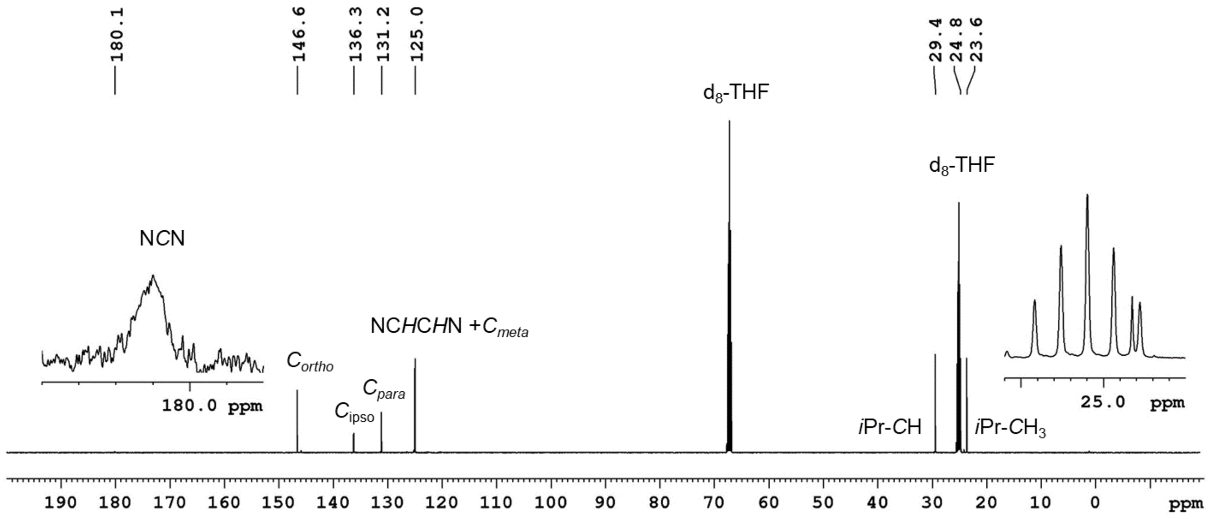

Figure S30: $^{13}C\{^1H\}$ NMR spectrum (125.8 MHz) upon dissolution of $[\{(Dipp_2Im)Cu\}_2]$**FAP**$_2$ (**4**) in *d*$_8$-THF.

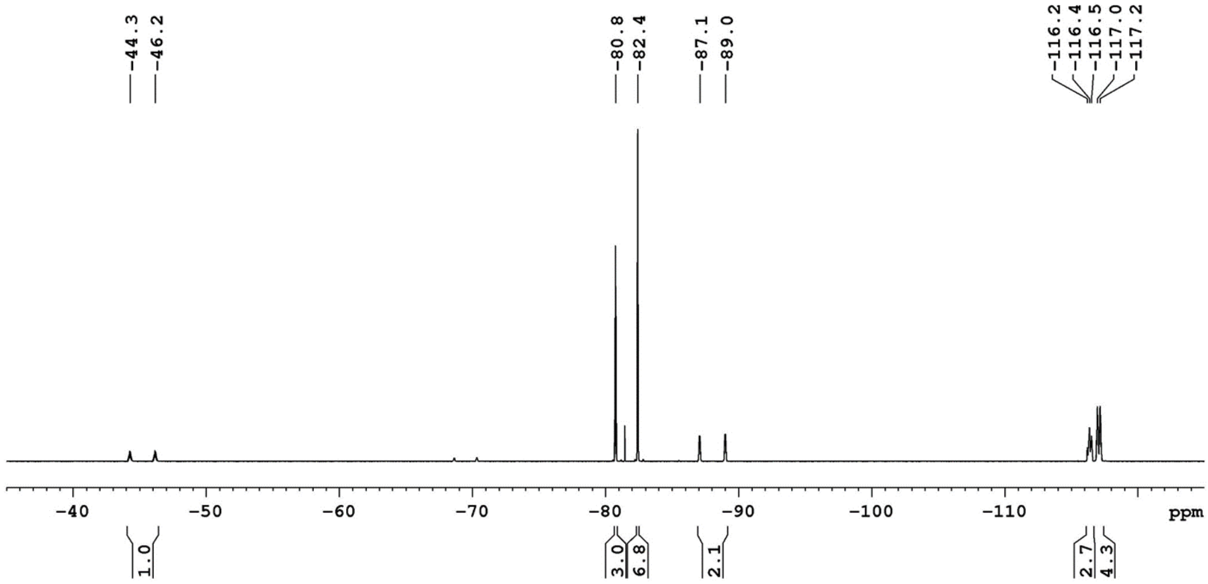

Figure S31: ^{19}F NMR spectrum (470.6 MHz) upon dissolution of $[\{(Dipp_2Im)Cu\}_2]$**FAP**$_2$ (**4**) in *d*$_8$-THF.

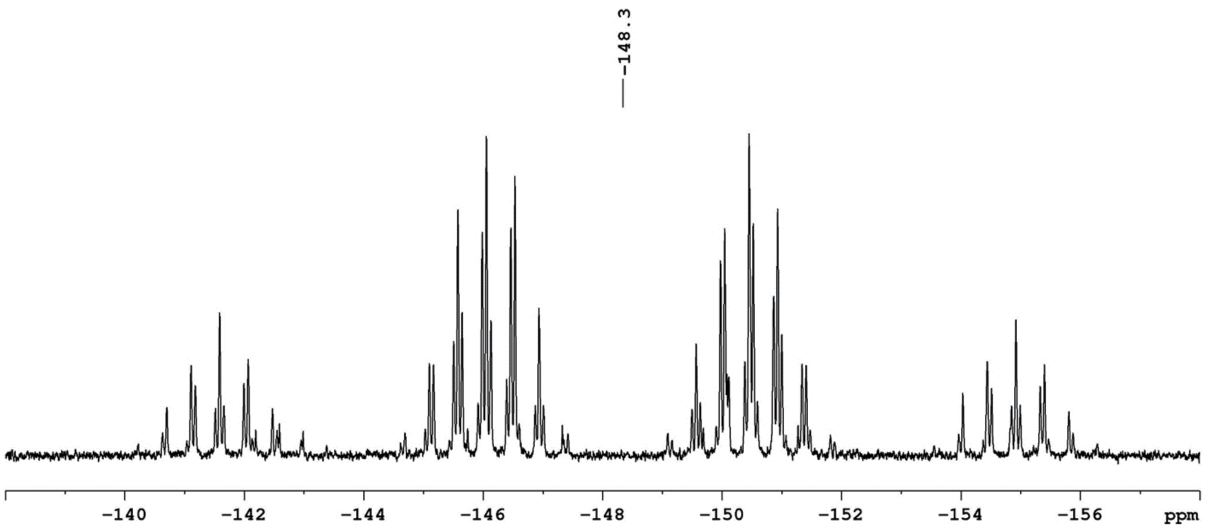

Figure S32: ^{31}P NMR spectrum (202.4 MHz) upon dissolution of [{(Dipp$_2$Im)Cu}$_2$]**FAP**$_2$ (**4**) in d_8-THF.

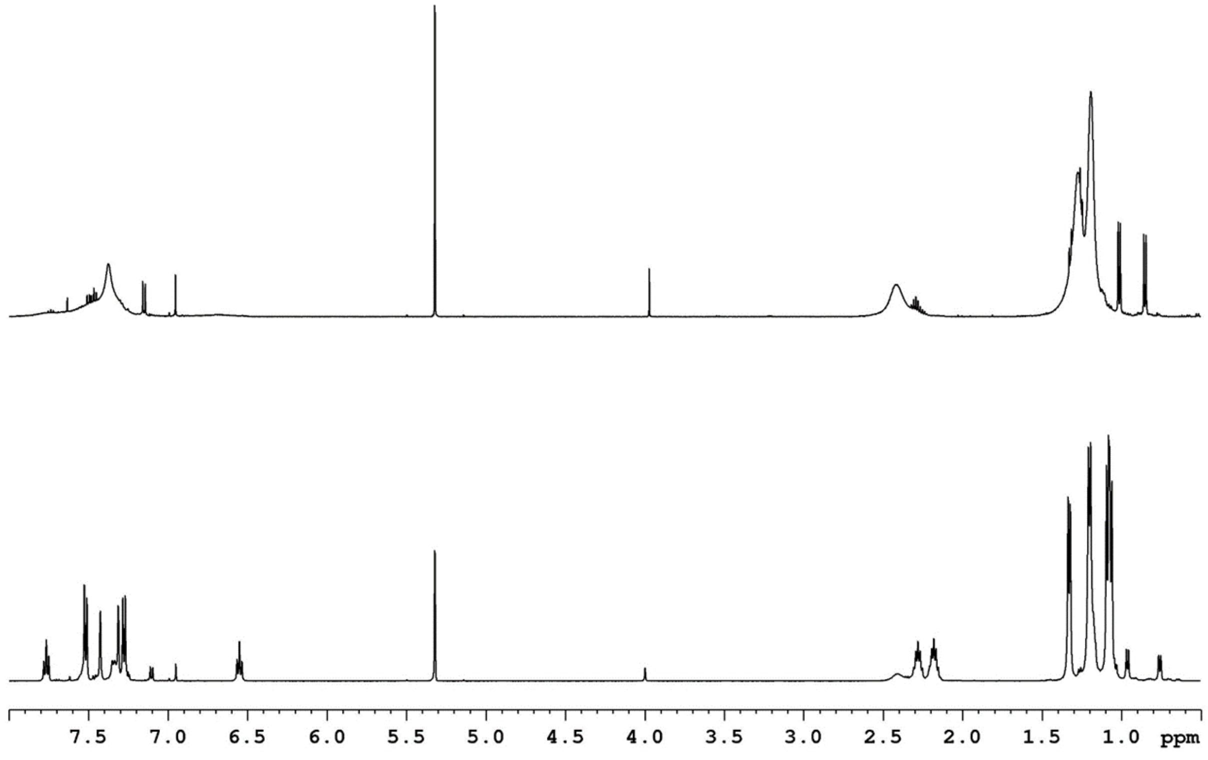

Figure S33: ^{1}H NMR spectrum (500.1 MHz) of [{(Dipp$_2$Im)Cu}$_2$]**FAP**$_2$ (**4**) in CD_2Cl_2 recorded at rt (top) and −40 °C (bottom).

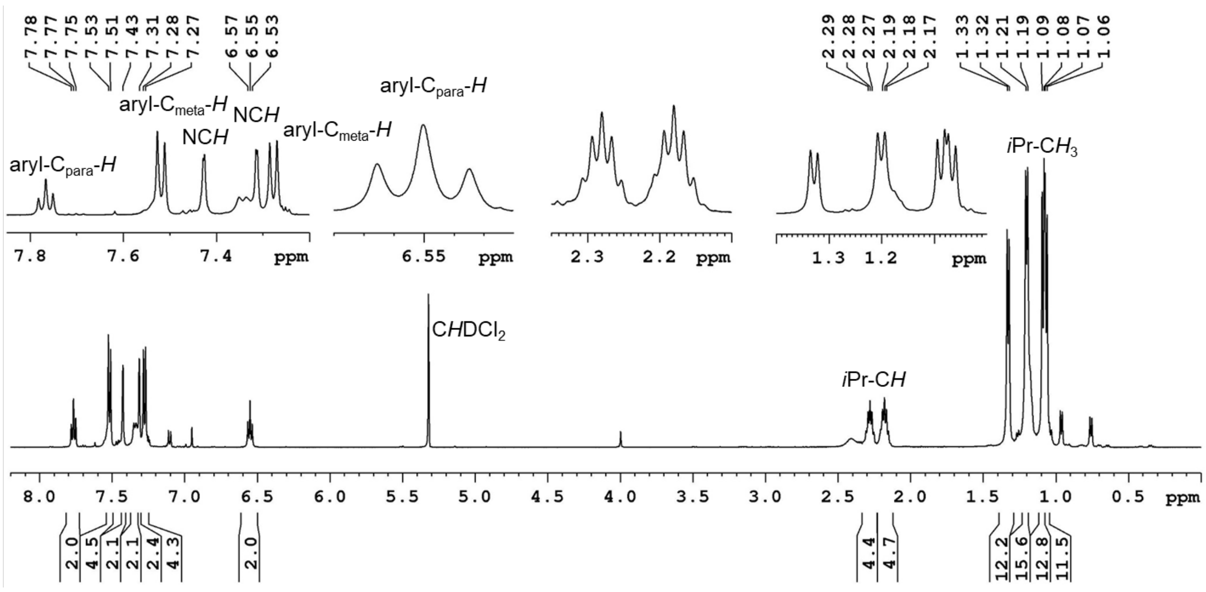

Figure S34: ^{1}H NMR spectrum (500.1 MHz) of [{(Dipp$_2$Im)Cu}$_2$]**FAP**$_2$ (**4**) in CD_2Cl_2 recorded at −40 °C.

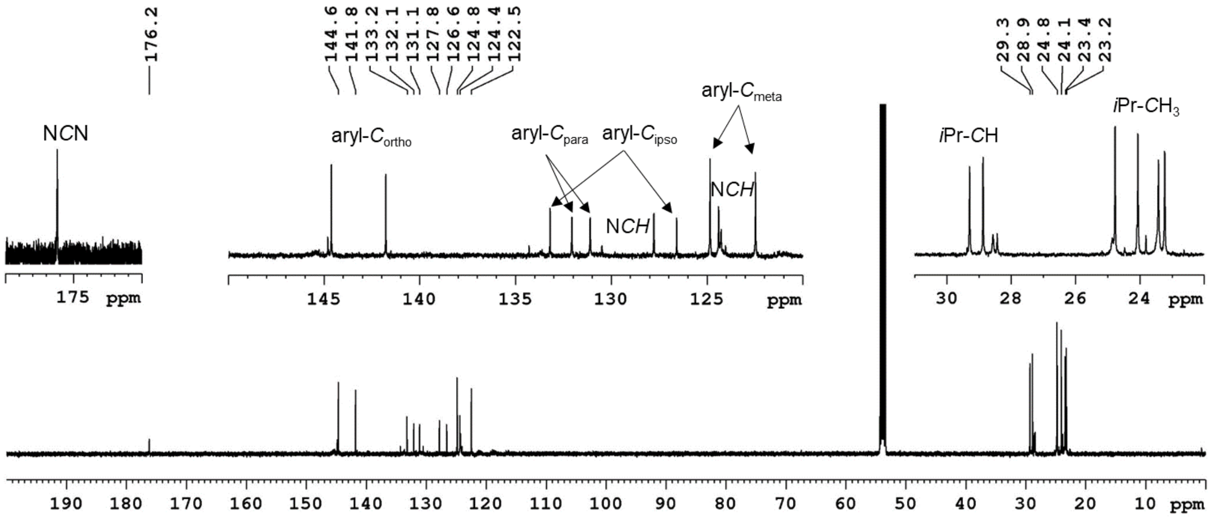

Figure S35: ^{13}C{^{1}H} NMR spectrum (125.8 MHz) of [{(Dipp$_2$Im)Cu}$_2$]**FAP**$_2$ (**4**) in CD_2Cl_2 recorded at −40°C.

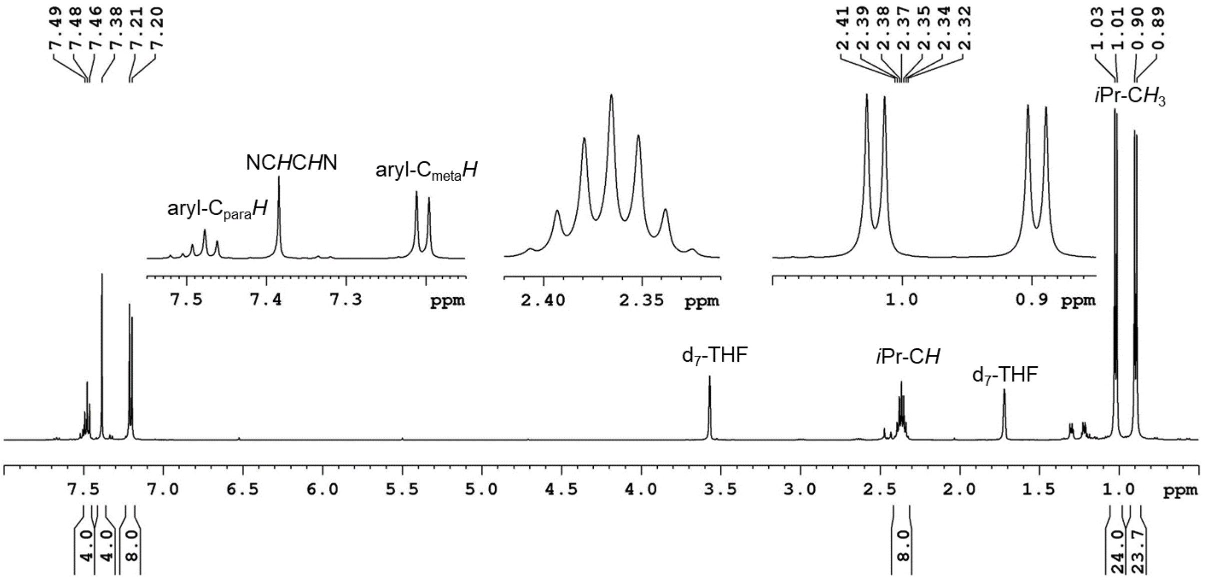

Figure S36: ^{1}H NMR spectrum (500.1 MHz) of [(Dipp$_2$Im)$_2$Cu]**FAP** (**5a**) recorded in d$_8$-THF.

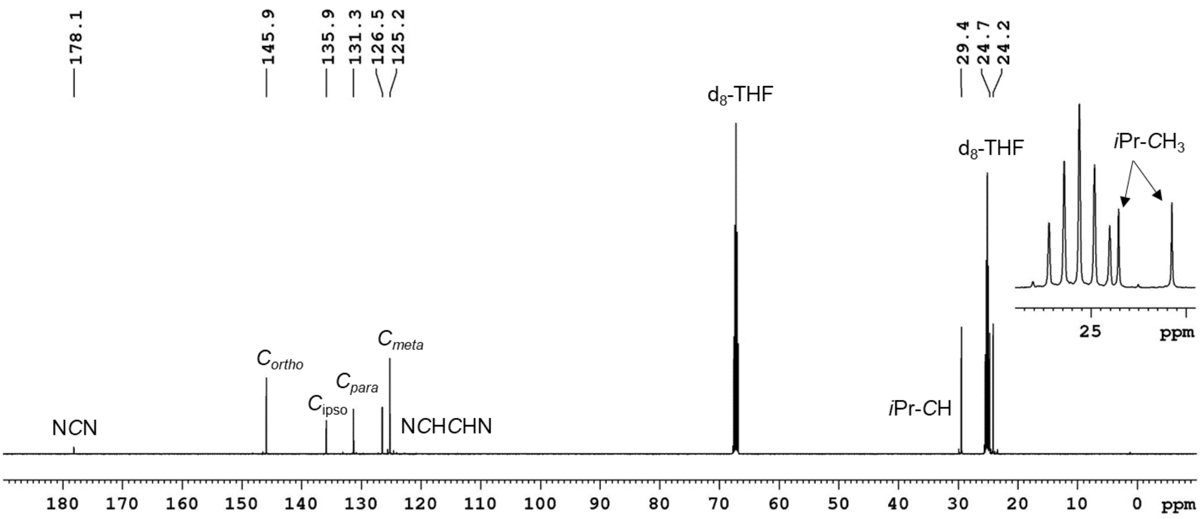

Figure S37: ^{13}C{^{1}H} NMR spectrum (125.8 MHz) of [(Dipp$_2$Im)$_2$Cu]**FAP** (**5a**) recorded in d_8-THF.

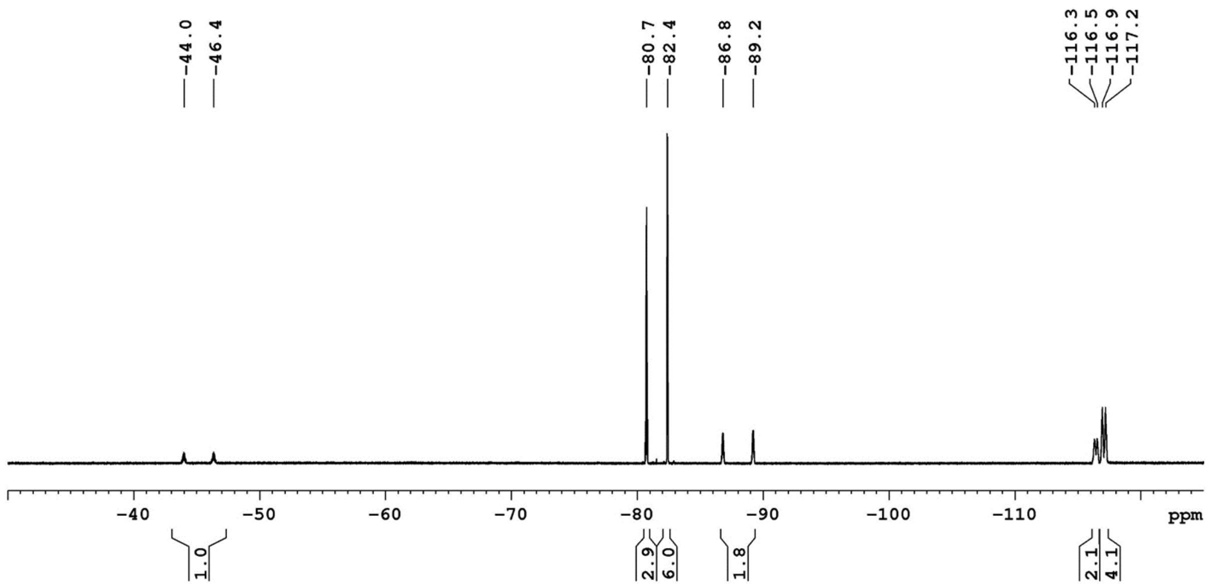

Figure S38: ^{19}F NMR spectrum (470.6 MHz) of [(Dipp$_2$Im)$_2$Cu]**FAP** (**5a**) recorded in d_8-THF.

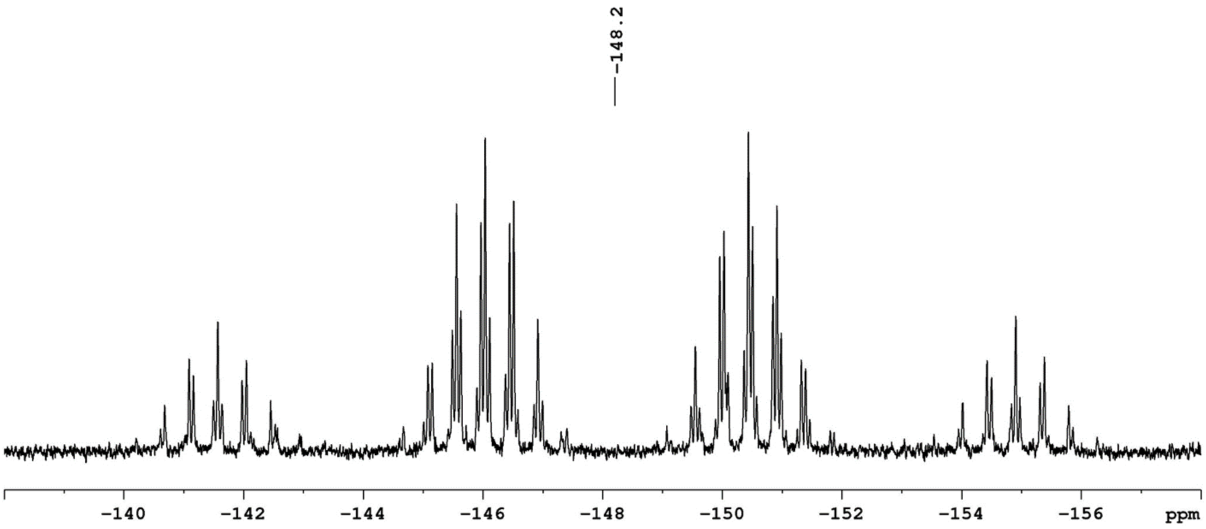

Figure S39: ^{31}P NMR spectrum (202.4 MHz) of [(Dipp$_2$Im)$_2$Cu]**FAP** (**5a**) recorded in d_8-THF.

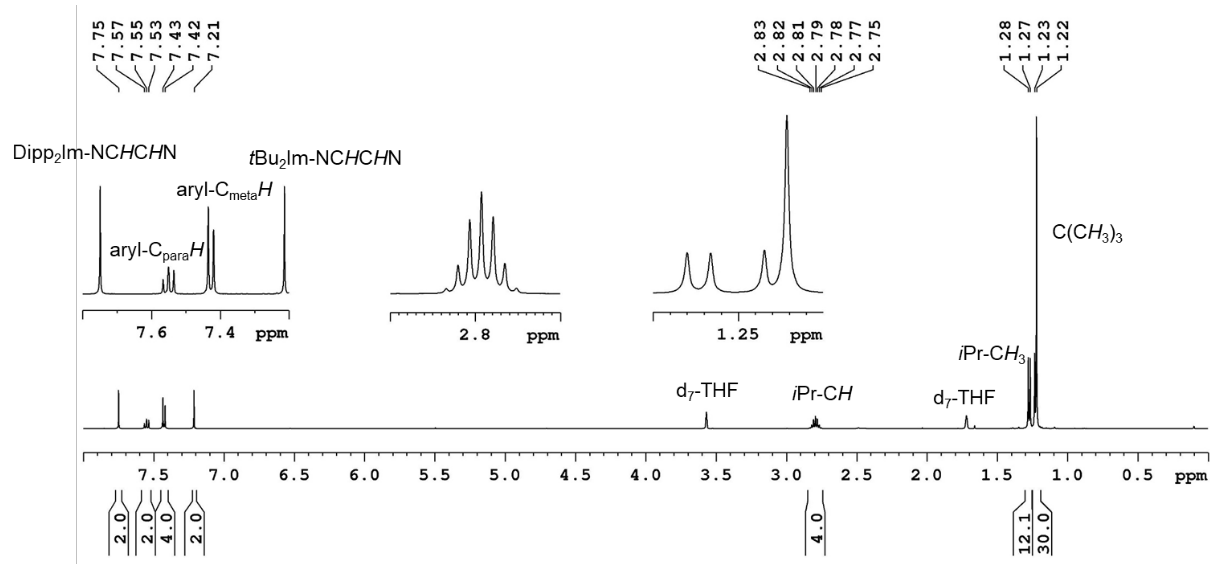

Figure S40: ^{1}H NMR spectrum (500.1 MHz) of [(Dipp$_2$Im)Cu(*t*Bu$_2$Im)]**FAP** (**5b**) recorded in *d*$_8$-THF.

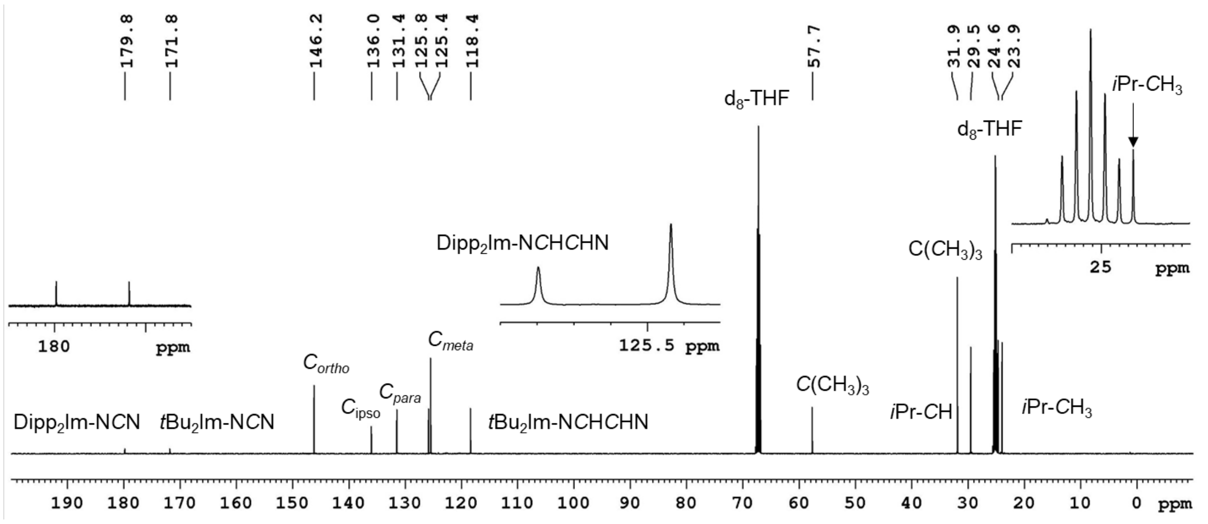

Figure S41: ^{13}C{^{1}H} NMR spectrum (125.8 MHz) of [(Dipp$_2$Im)Cu(*t*Bu$_2$Im)]**FAP** (**5b**) recorded in *d*$_8$-THF.

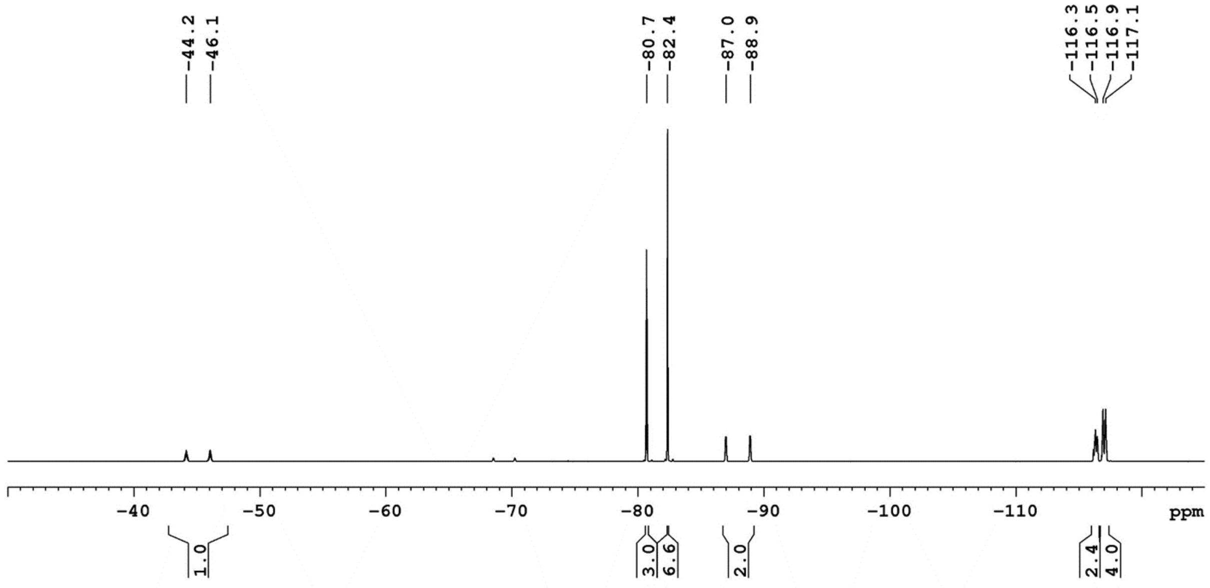

Figure S42: ^{19}F NMR spectrum (470.6 MHz) of [(Dipp$_2$Im)Cu(*t*Bu$_2$Im)]**FAP** (**5b**) recorded in *d*$_8$-THF.

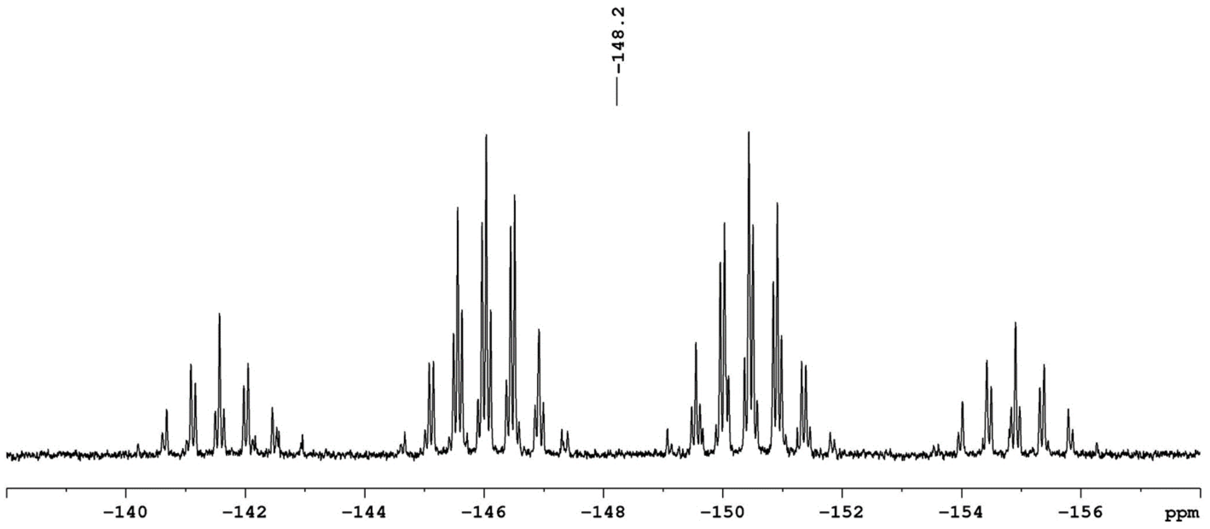

Figure S43: ^{31}P NMR spectrum (202.4 MHz) of [(Dipp$_2$Im)Cu(*t*Bu$_2$Im)]**FAP** (**5b**) recorded in *d*$_8$-THF.

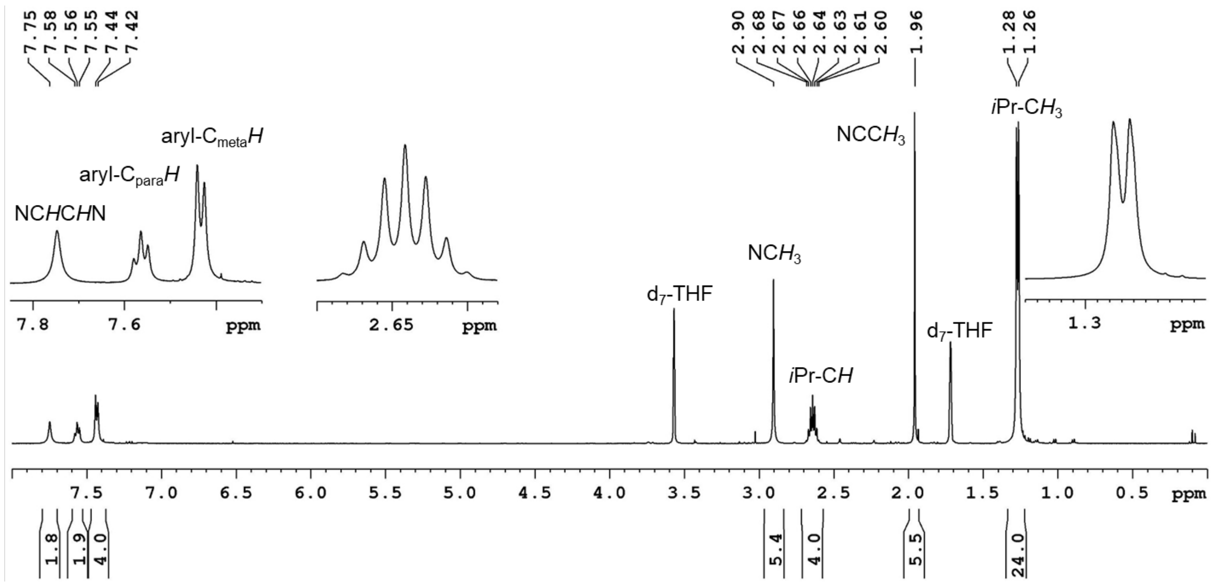

Figure S44: ^{1}H NMR spectrum (500.1 MHz) of [(Dipp$_2$Im)Cu(Me$_2$Im^{Me})]**FAP** (**5c**) recorded in *d*$_8$-THF.

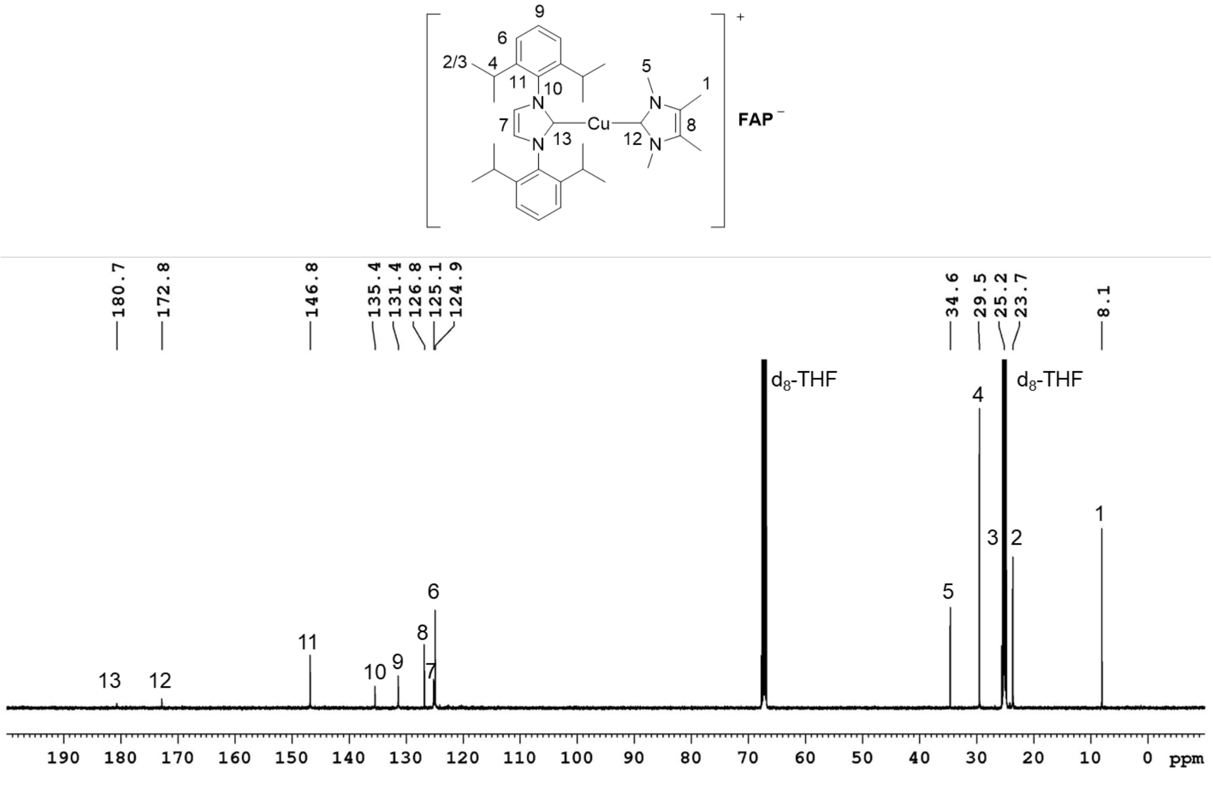

Figure S45: ^{13}C{^{1}H} NMR spectrum (125.8 MHz) of [(Dipp$_2$Im)Cu(Me$_2$Im^{Me})]**FAP** (**5c**) recorded in *d*$_8$-THF.

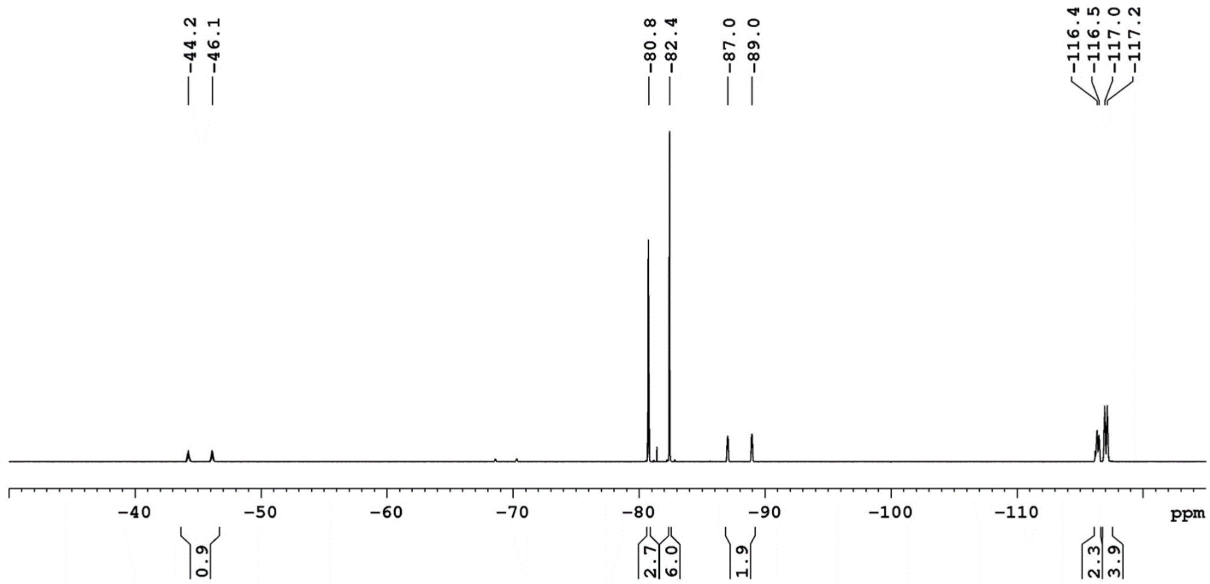

Figure S46: ^{19}F NMR spectrum (470.6 MHz) of [(Dipp$_2$Im)Cu(Me$_2$Im^{Me})]**FAP** (**5c**) recorded in d_8-THF.

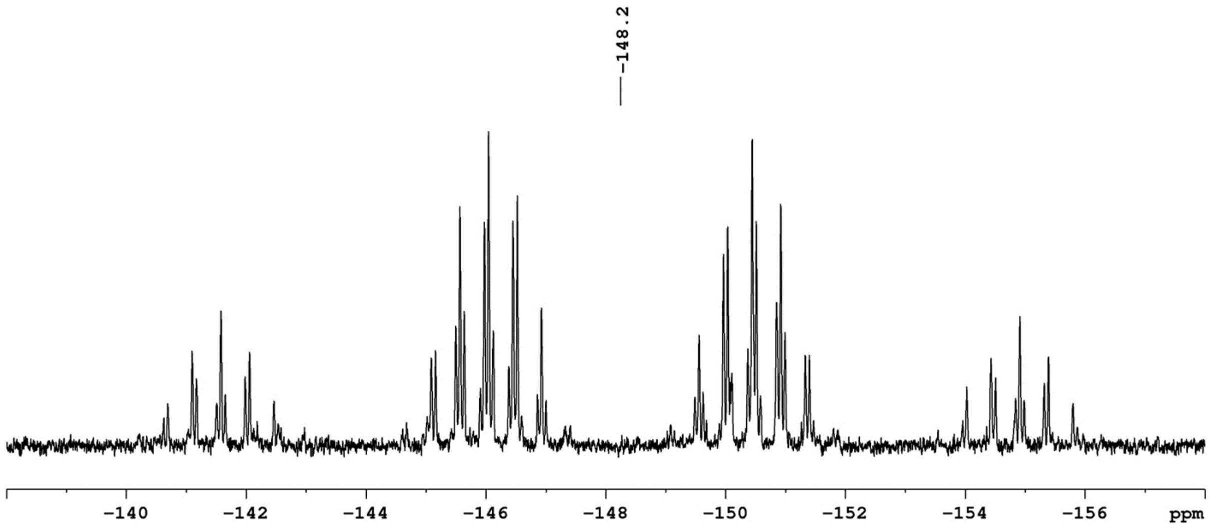

Figure S47: ^{31}P NMR spectrum (202.4 MHz) of [(Dipp$_2$Im)Cu(Me$_2$Im^{Me})]**FAP** (**5c**) recorded in d_8-THF.

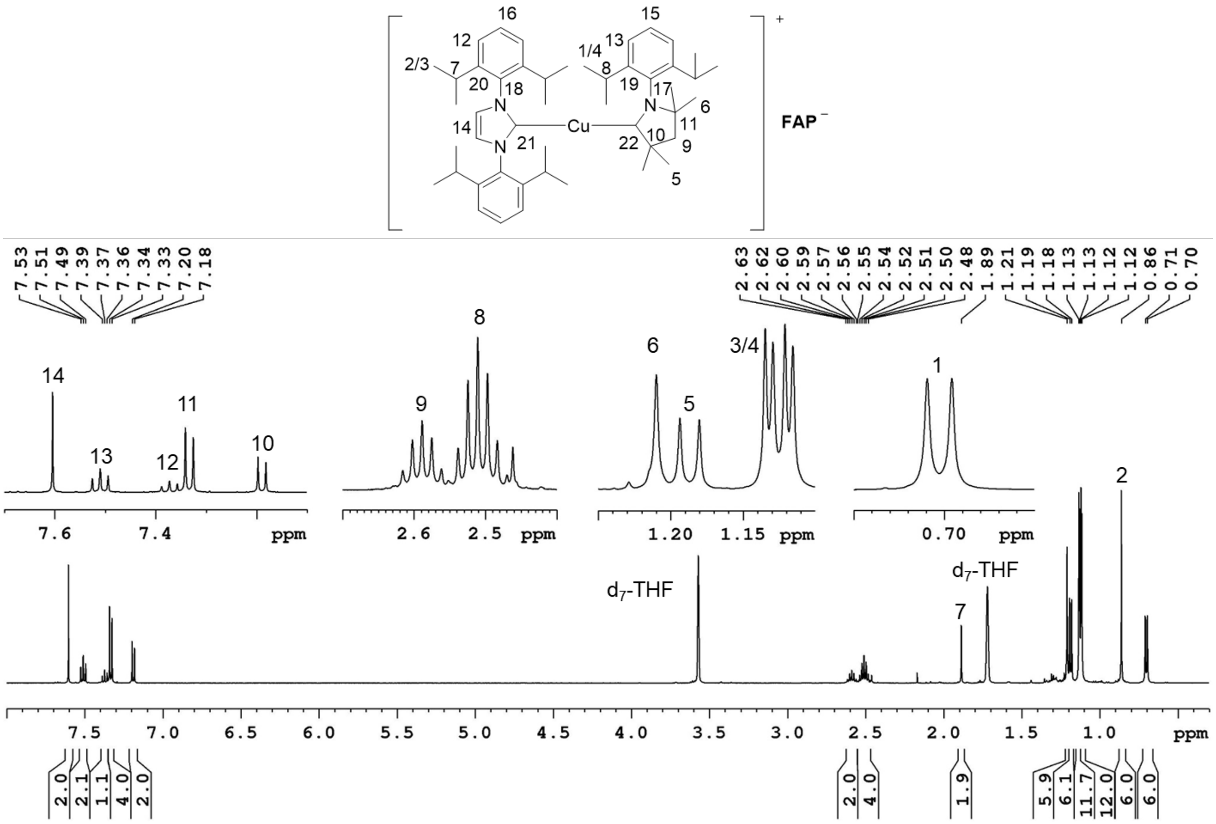

Figure S48: ^{1}H NMR spectrum (500.1 MHz) of [(Dipp$_2$Im)Cu(cAAC^{Me})]**FAP** (**5d**) recorded in *d*$_8$-THF.

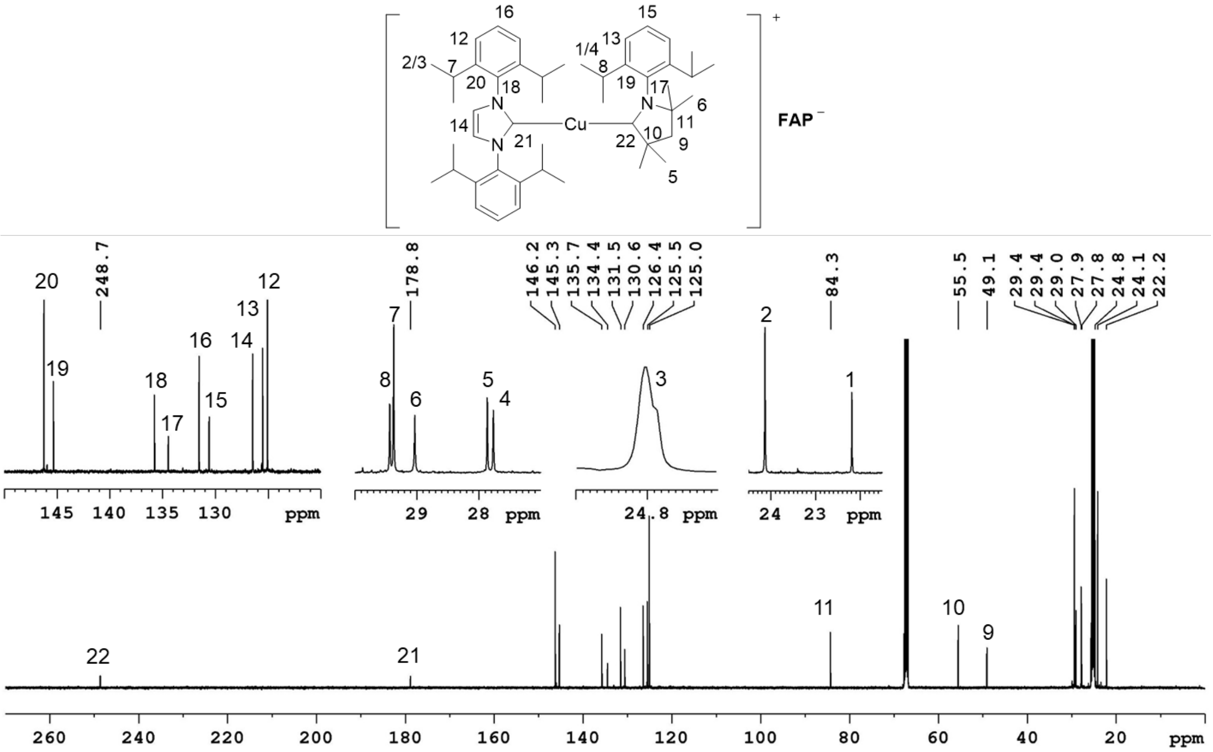

Figure S49: ^{13}C{^{1}H} NMR spectrum (125.8 MHz) of [(Dipp$_2$Im)Cu(cAAC^{Me})]**FAP** (**5d**) recorded in *d*$_8$-THF.

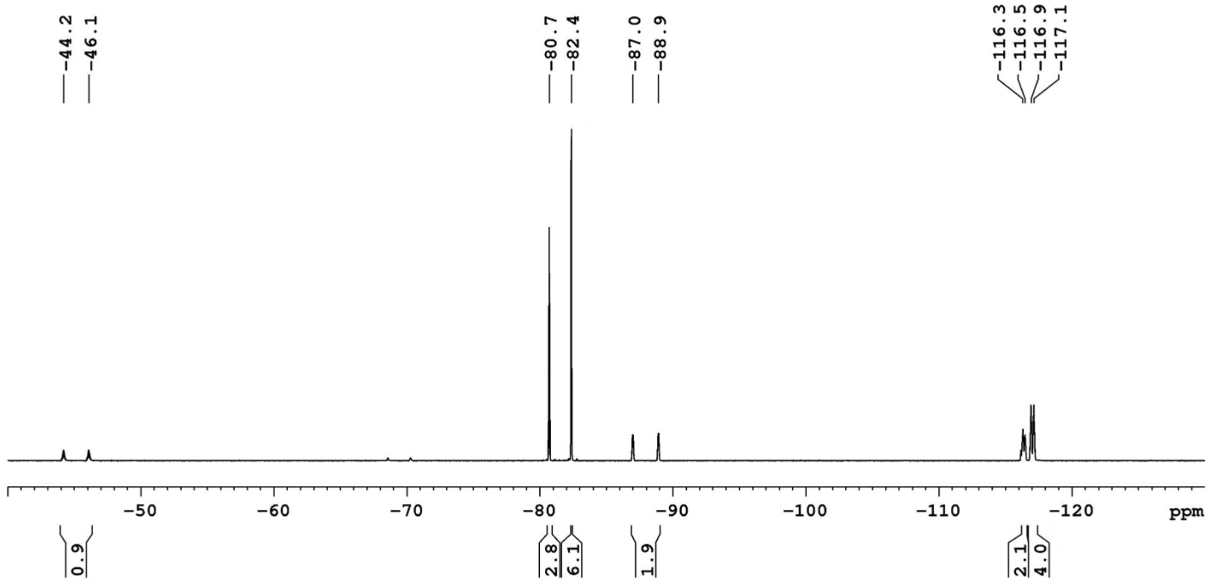

Figure S50: ^{19}F NMR spectrum (470.5 MHz) of [(Dipp$_2$Im)Cu(cAAC^{Me})]**FAP** (**5d**) recorded in d_8-THF.

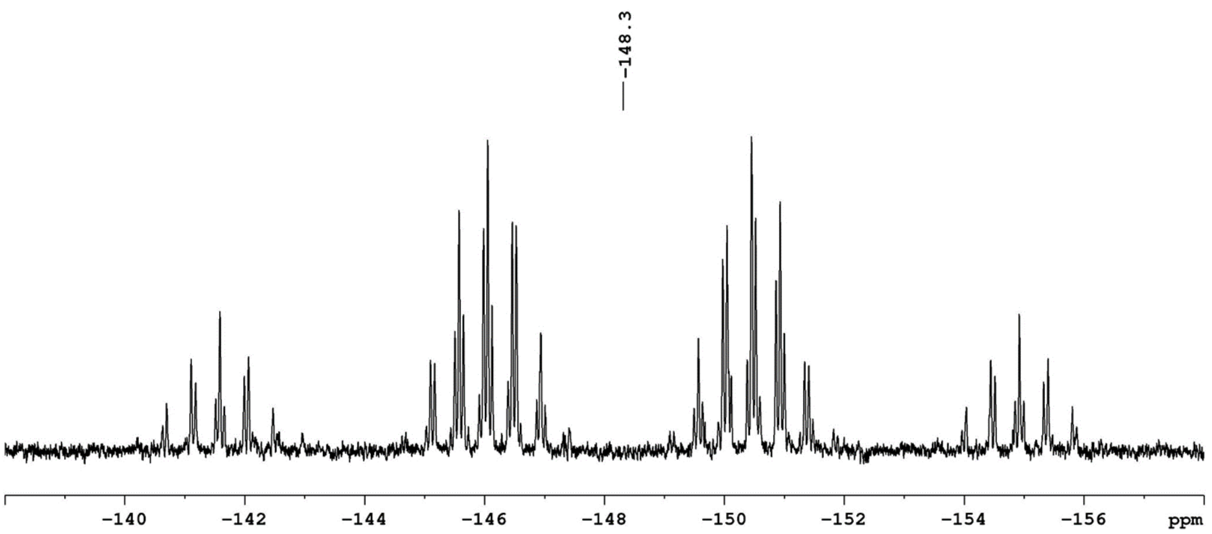

Figure S51: ^{31}P NMR spectrum (202.4 MHz) of [(Dipp$_2$Im)Cu(cAAC^{Me})]**FAP** (**5d**) recorded in d_8-THF.

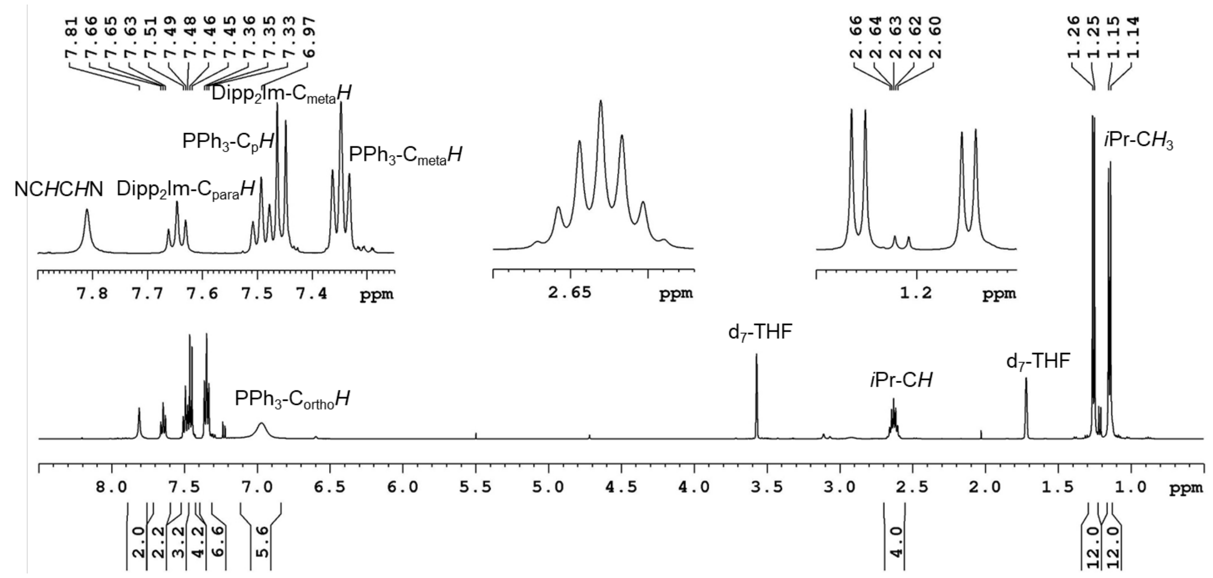

Figure S52: 1H NMR spectrum (500.1 MHz) of [(Dipp$_2$Im)Cu(PPh$_3$)]**FAP** (**5e**) recorded in d_8-THF.

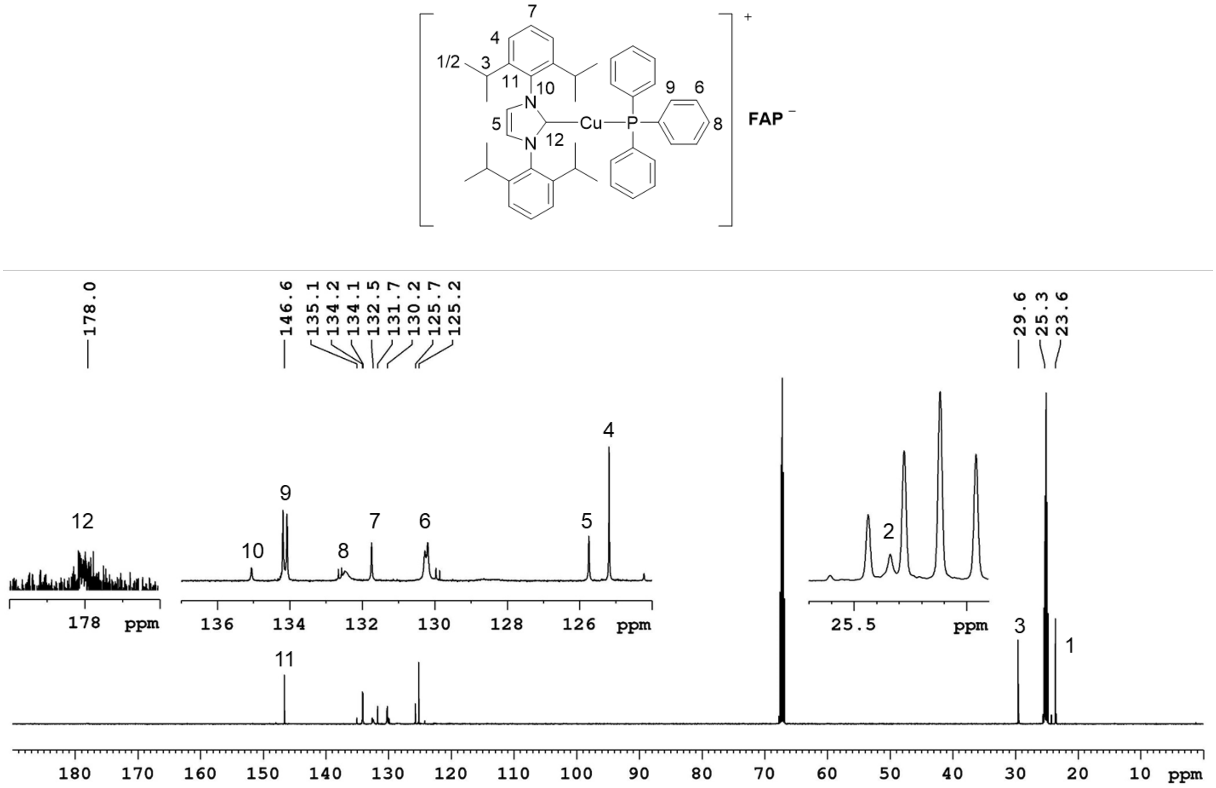

Figure S53: $^{13}C\{^{1}H\}$ NMR spectrum (125.8 MHz) of [(Dipp$_2$Im)Cu(PPh$_3$)]**FAP** (**5e**) recorded in d_8-THF.

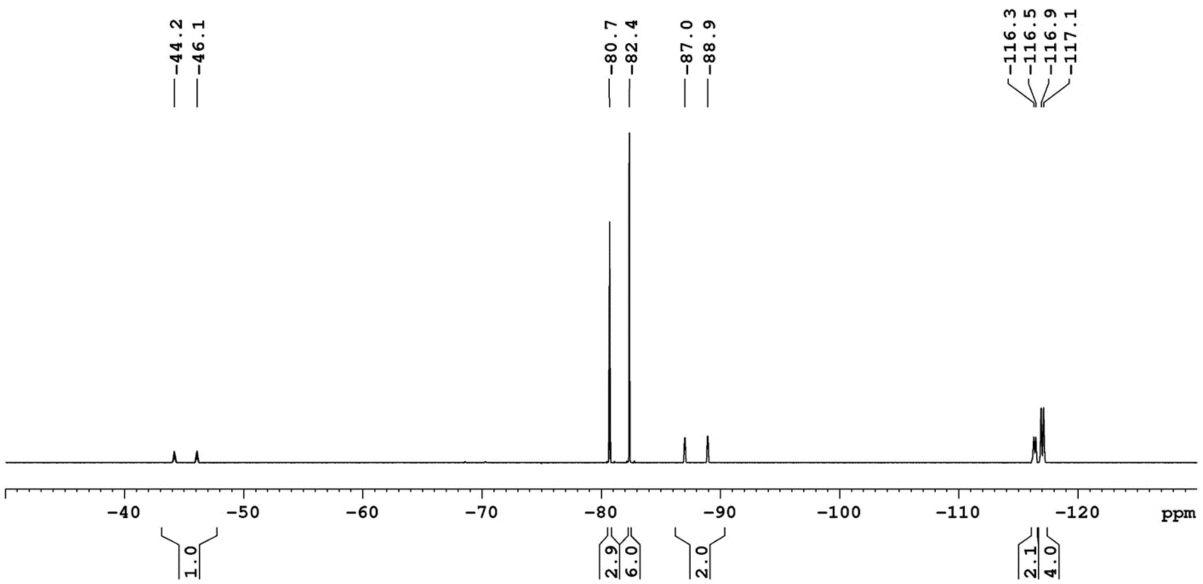

Figure S54: ^{19}F NMR spectrum (470.6 MHz) of [(Dipp$_2$Im)Cu(PPh$_3$)]**FAP** (**5e**) recorded in d_8-THF.

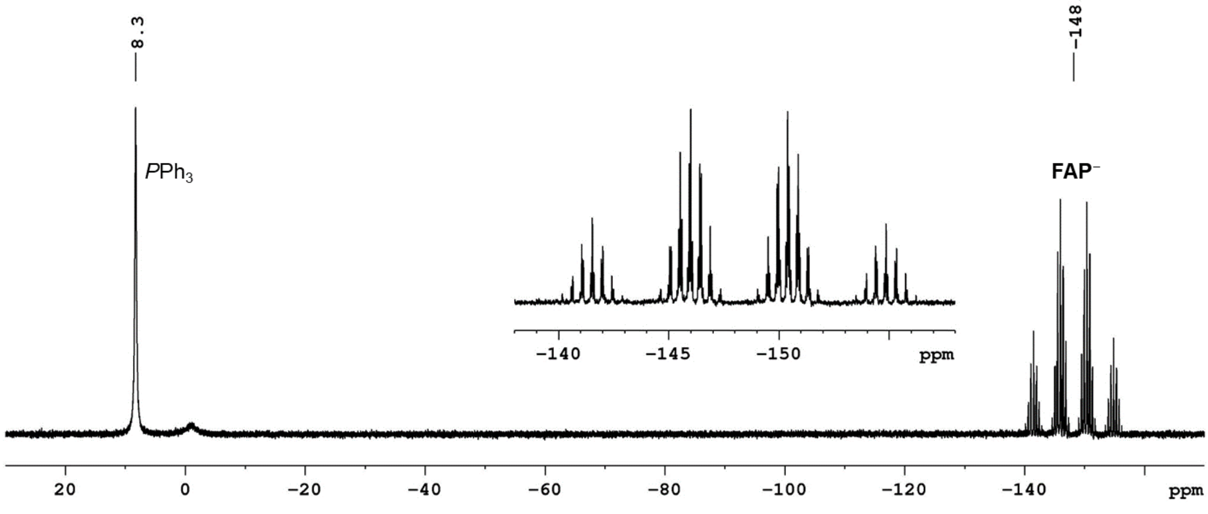

Figure S55: ^{31}P NMR spectrum (202.4 MHz) of [(Dipp$_2$Im)Cu(PPh$_3$)]**FAP** (**5e**) recorded in d_8-THF.

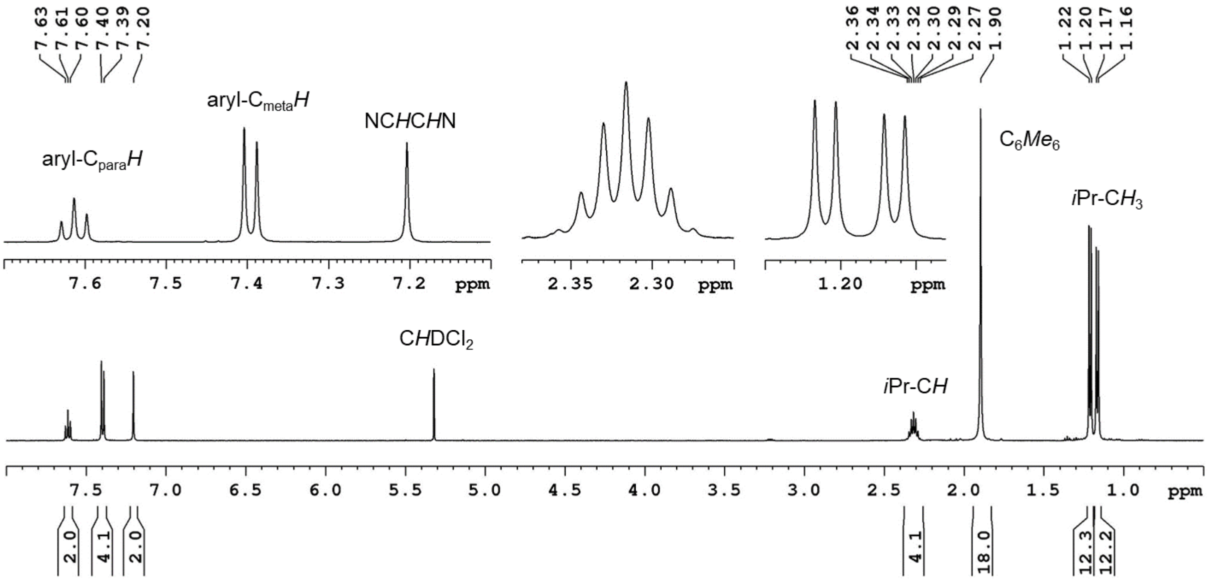

Figure S56: ^{1}H NMR spectrum (500.1 MHz) of [(Dipp$_2$Im)Cu(C$_6$Me$_6$)]**FAP** (**5f**) recorded in CD$_2$Cl$_2$.

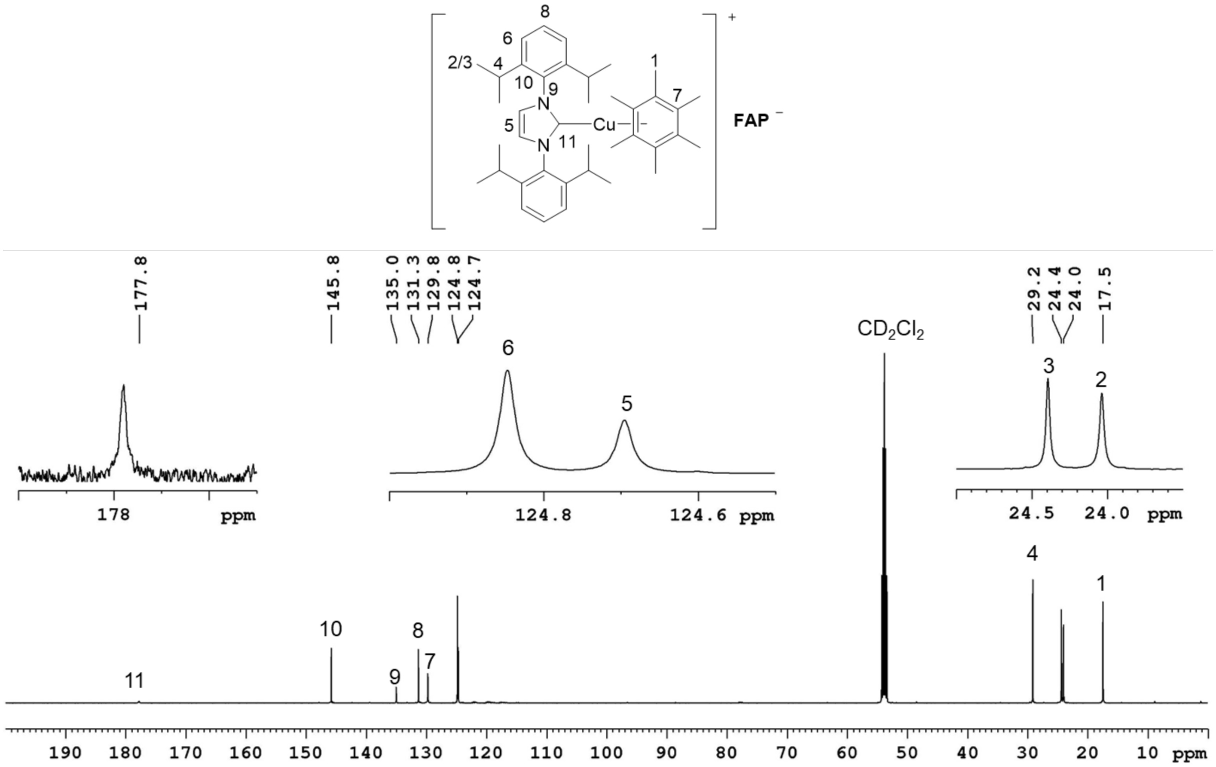

Figure S57: ^{13}C{^{1}H} NMR spectrum (125.8 MHz) of [(Dipp$_2$Im)Cu(C$_6$Me$_6$)]**FAP** (**5f**) recorded in CD$_2$Cl$_2$.

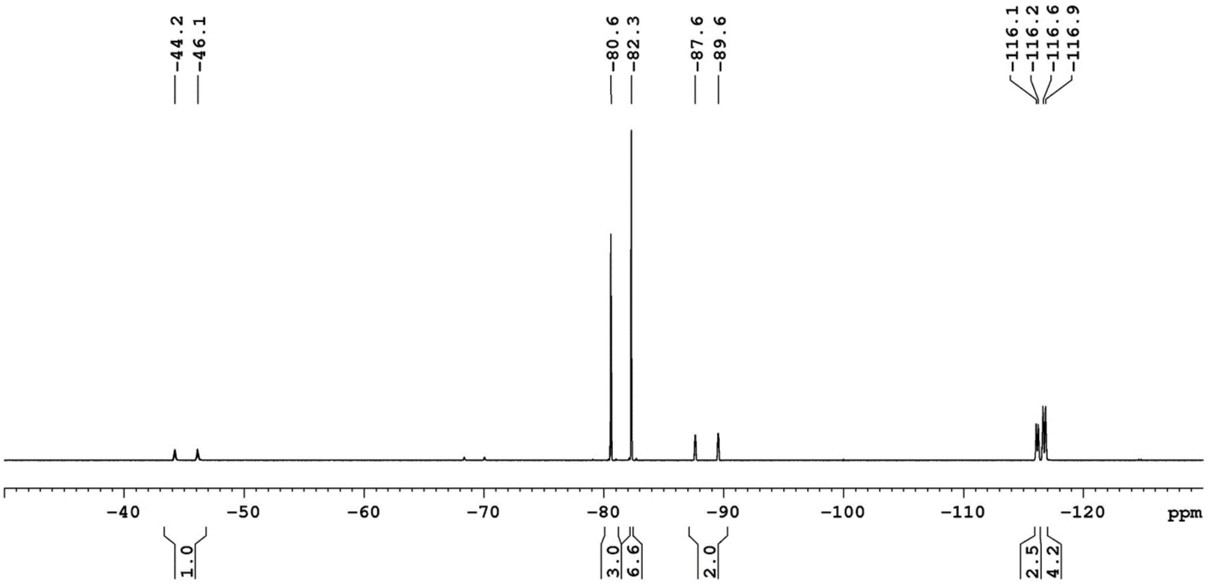

Figure S58: ^{19}F NMR spectrum (470.6 MHz) of [(Dipp$_2$Im)Cu(C$_6$Me$_6$)]**FAP** (**5f**) recorded in CD_2Cl_2.

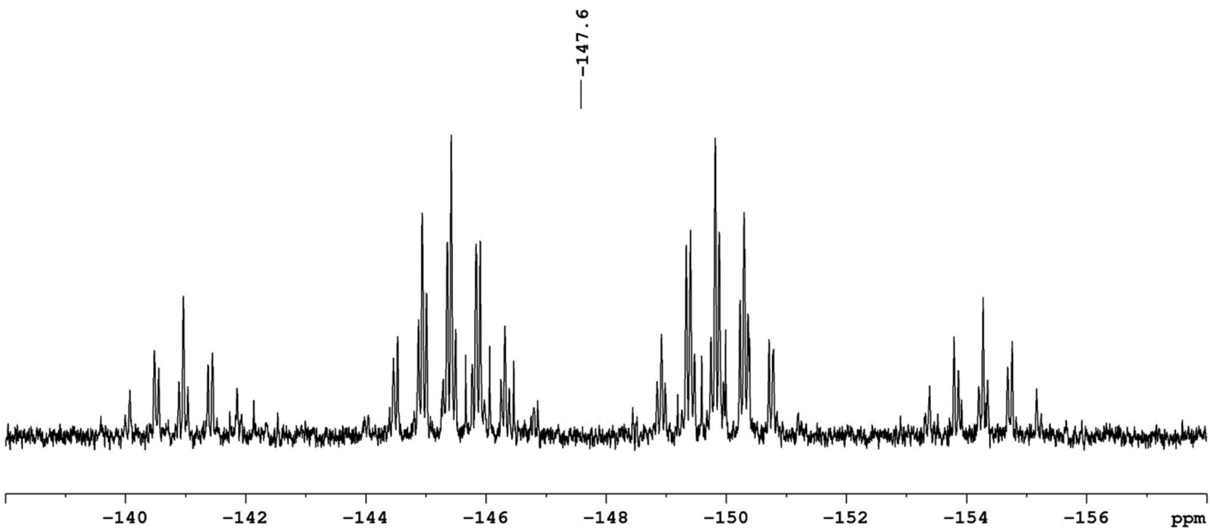

Figure S59: ^{31}P NMR spectrum (202.4 MHz) of [(Dipp$_2$Im)Cu(C$_6$Me$_6$)]**FAP** (**5f**) recorded in CD_2Cl_2.

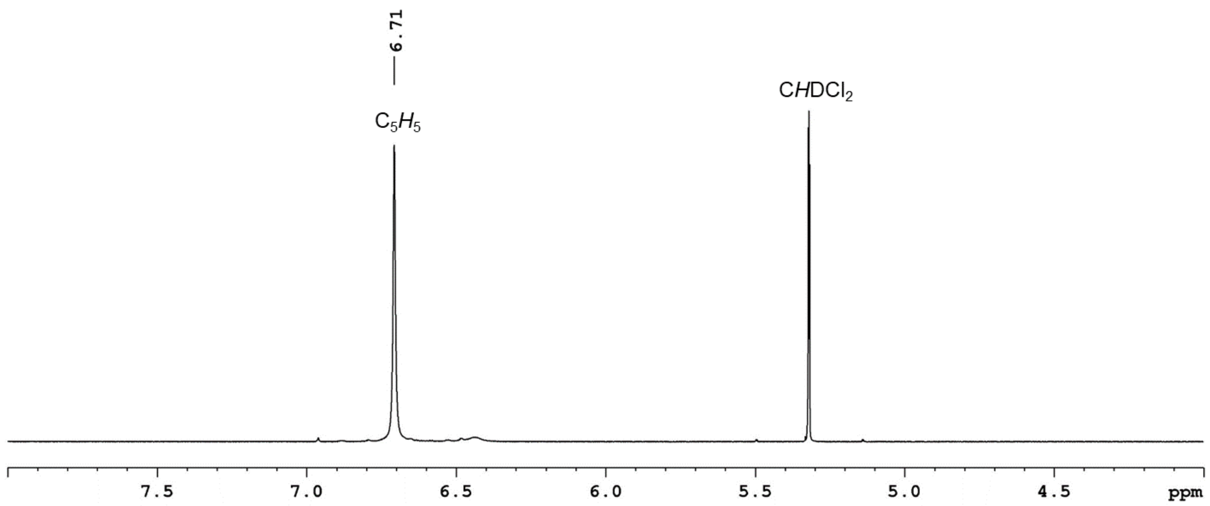

Figure S60: ^{1}H NMR spectrum (500.1 MHz) of [(F)(Cp)$_2$Ti(μ-F)Ti(Cp)$_2$(F)]**FAP** (**7**) recorded in CD_2Cl_2.

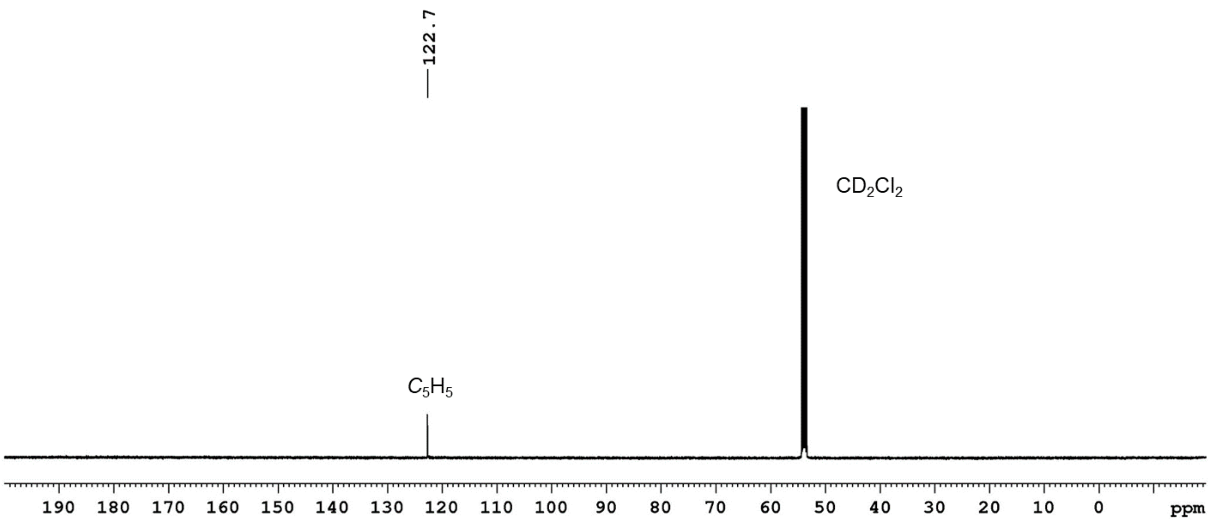

Figure S61: $^{13}C\{^{1}H\}$ NMR spectrum (125.8 MHz) of [(F)(Cp)$_2$Ti(μ-F)Ti(Cp)$_2$(F)]**FAP** (**7**) recorded in CD_2Cl_2.

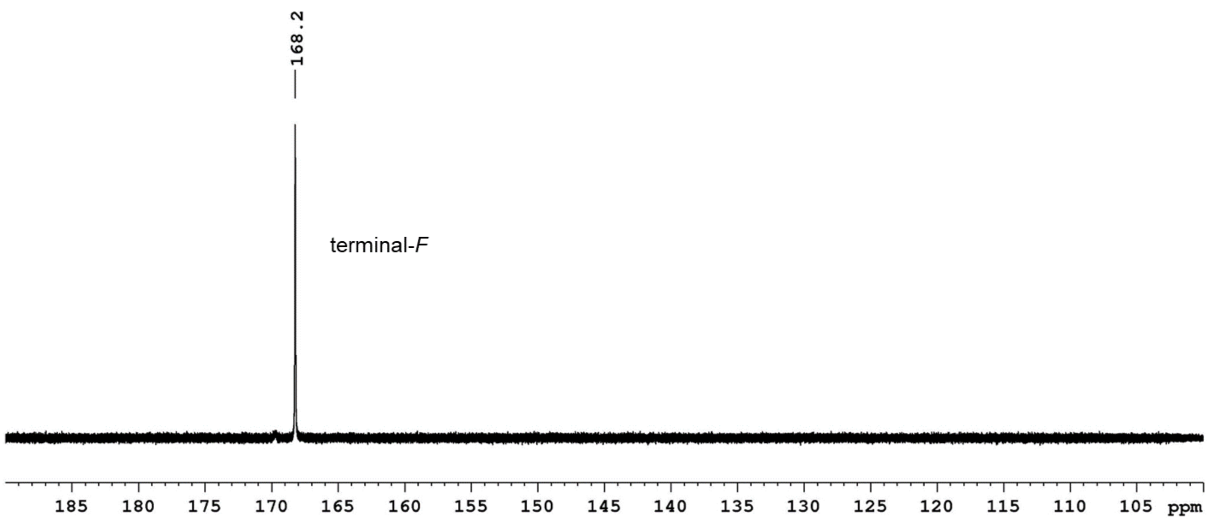

Figure S62: ^{19}F NMR spectrum (470.6 MHz) of [(F)(Cp)$_2$Ti(µ-F)Ti(Cp)$_2$(F)]**FAP** (**7**) in the range between 190 and 100 ppm recorded in CD_2Cl_2.

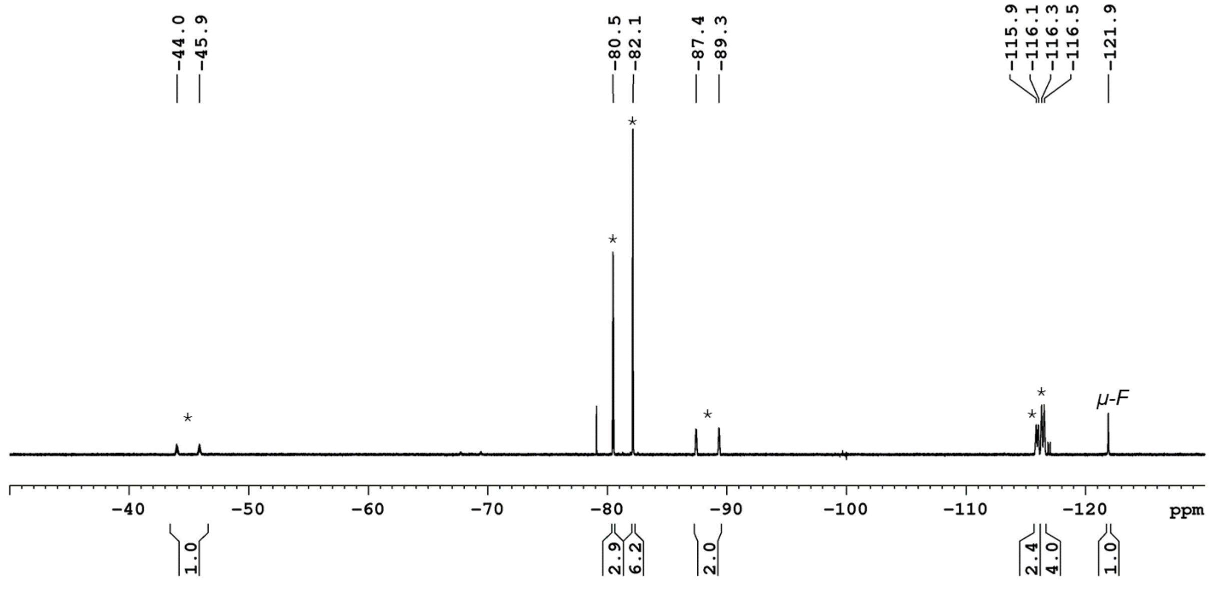

Figure S63: ^{19}F NMR spectrum (470.6 MHz) of [(F)(Cp)$_2$Ti(µ-F)Ti(Cp)$_2$(F)]**FAP** (**7**) in the range between −40 and −130 ppm recorded in CD_2Cl_2; the asterisks (*) indicate resonances of the anion.

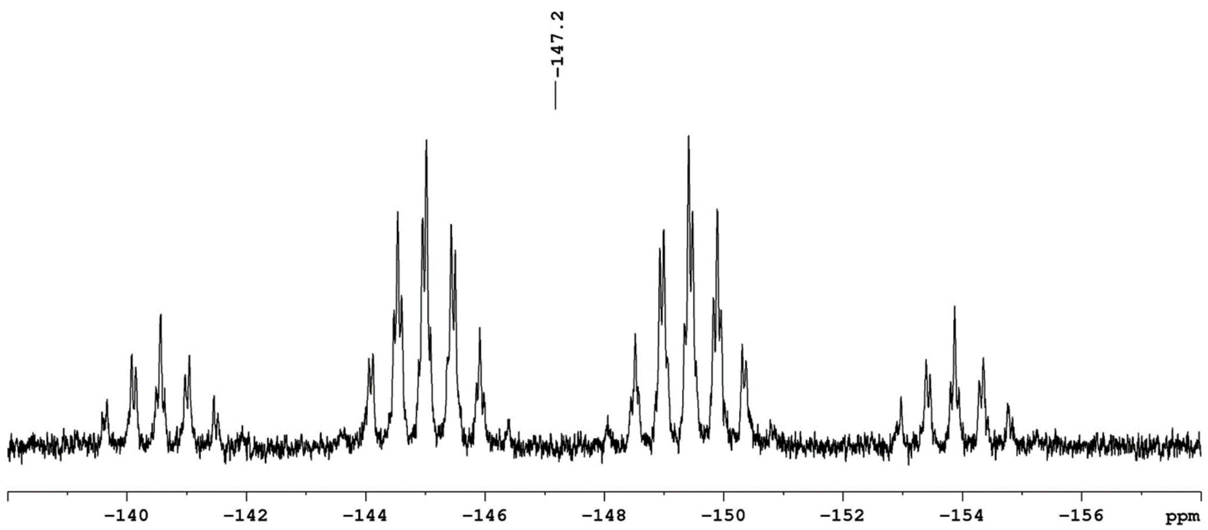

Figure S64: ^{31}P NMR spectrum (202.4 MHz) of [(F)(Cp)$_2$Ti(μ-F)Ti(Cp)$_2$(F)]**FAP** (**7**) recorded in CD_2Cl_2.

3) Crystallographic Details

Crystal data were collected on a Bruker X8 Apex-2 diffractometer with a CCD area detector and graphite-monochromated Mo-Kα radiation or a Rigaku XtaLAB Synergy-DW diffractometer with an Hy-Pix-6000HE detector and monochromated Cu-*Kα* radiation equipped with an Oxford Cryo 800 cooling unit. Crystals were immersed in a film of perfluoropolyether oil on a MicroMount™ and data were collected at 100 K. Images were processed with Bruker or CrySalis software packages and the structures were solved using the ShelXTL software package.[S4] All non-hydrogen atoms were refined anisotropically and all hydrogen atoms were assigned to idealized geometric positions.

Crystal Data for *trans*-[Ni(*i*Pr$_2$Im)$_2$(OH$_2$)$_2$(4-(C$_6$F$_5$)(C$_6$F$_4$)]FAP (*trans*-2c[OH$_2$]·H$_2$O): $C_{36}H_{36}F_{27}N_4NiO_2P$, M_r = 1159.37, T = 100(2) K, λ = 0.71073 Å, yellow block, 0.200×0.381×0.609 mm^3, triclinic space group $P\bar{1}$, a = 11.9629(8) Å, b = 12.7813(8) Å, c = 15.5432(7) Å, α = 98.539(2) °, β = 97.647(2) °, γ = 99.636(2) °, V = 2286.5(2) Å^3, Z = 2, ρ_{calcd} = 1.684 Mg/m^3, μ = 0.608 mm^{-1}, F(000) = 1164, 10031 reflections, −6 ≤ h ≤ 15, −17 ≤ k ≤ 16, −17 ≤ l ≤ 17, 1.342 ° < θ < 28.332 °, completeness 83.1 %, 8914 independent reflections, 7461 reflections observed with [I > 2σ(I)], 664 parameters, 0 restraints, R indices (all data) R_1 = 0.0426, wR_2 = 0.0809, final R indices [I > 2σ(I)] R_1 = 0.0327, wR_2 = 0.076, largest difference peak and hole 0.350 and −0.317 e Å^{-3}, Goof = 1.020. Due to technical problems during data acquisition (which led to decomposition of the crystal) the measurement was cancelled at a late stage of data acquisition. Afterwards, we were not able to obtain other crystals of ***trans*-2c[OH$_2$]·H$_2$O)** suitable for X-ray diffraction. However, as the measured fraction is 0.831, ranging from 2θ = 2.684° to 2θ = 55.664°, which led to the collection of 8914 independent reflections, 7461 reflections with I > 2σ(I). As 664 parameters were refined with this data set, the parameter: data ratio should be sufficient (> 1:10) for a reliable and meaningful X-ray crystal structure determination. The hydrogen atoms of the water molecules were freely refined.

Crystal Data for *trans*-[Ni(*i*Pr$_2$Im)$_2$(PPh$_3$)(C$_6$F$_5$)]FAP (*trans*-2a[PPh$_3$]): $C_{48}H_{47}F_{23}N_4NiP_2$, M_r = 1237.52, T = 100(2) K, λ = 0.71073 Å, yellow block, 0.030×0.186×0.257 mm^3, triclinic space group $P\bar{1}$, a = 12.0589(18) Å, b = 12.5582(18) Å, c = 18.791(3) Å, α = 74.694(5) °, β = 74.732(5) °, γ = 85.768(5) °, V = 2647.8(7) Å^3, Z = 2, ρ_{calcd} = 1.552 Mg/m^3, μ = 0.546 mm^{-1}, F(000) = 1256, 26977

reflections, $-15 \leq h \leq 15$, $-15 \leq k \leq 15$, $-23 \leq l \leq 23$, 1.784 ° < θ < 26.372 °, completeness 99.5 %, 10773 independent reflections, 6579 reflections observed with [I > 2σ(I)], 711 parameters, 0 restraints, R indices (all data) R_1 = 0.1166, wR_2 = 0.1573, final R indices [I > 2σ(I)] R_1 = 0.0610, wR_2 = 0.1339, largest difference peak and hole 1.085 and −0.438 e $Å^{-3}$, Goof = 1.017.

Crystal Data for *cis*-[Ni(*i*Pr$_2$Im)$_2$(Dipp$_2$Im)(C$_6$F$_5$)]FAP (*cis*-2a[Dipp$_2$Im]): $C_{57}H_{68}F_{23}N_6NiP$, M_r = 1363.85, T = 100(2) K, λ = 1.54184 Å, yellow block, 0.138×0.204×0.259 mm^3, triclinic space group $P\bar{1}$, a = 11.6240(2) Å, b = 13.7406(2) Å, c = 19.3628(3) Å, α = 85.8520(10) °, β = 84.1290(10) °, γ = 88.6860(10) °, V = 3067.98(8) $Å^3$, Z = 2, ρ_{calcd} = 1.476 Mg/m^3, μ = 1.715 mm^{-1}, F(000) = 1404, 64199 reflections, $-14 \leq h \leq 14$, $-17 \leq k \leq 16$, $-24 \leq l \leq 19$, 2.300 ° < θ < 74.497 °, completeness 99.8 %, 12523 independent reflections, 11207 reflections observed with [I > 2σ(I)], 1035 parameters, 1233 restraints, R indices (all data) R_1 = 0.0476, wR_2 = 0.1121, final R indices [I > 2σ(I)] R_1 = 0.0432, wR_2 = 0.1093, largest difference peak and hole 0.814 and −0.815 e $Å^{-3}$, Goof = 1.035.

Crystal Data for [{(Dipp$_2$Im)Cu)}$_2$]FAP$_2$ (4): $C_{54}H_{72}Cu_2N_4$, 2($C_6F_{18}P$), 1.5(CH_2Cl_2), M_r = 963.24, T = 100(2) K, λ = 0.71073 Å, colorless block, 0.263×0.263×0.379 mm^3, monoclinic space group $P2_1/n$, a = 13.4456(9) Å, b = 20.6118(15) Å, c = 15.1542(11) Å, β = 103.702(3) °, V = 4080.3(5) $Å^3$, Z = 4, ρ_{calcd} = 1.568 Mg/m^3, μ = 0.785 mm^{-1}, F(000) = 1945, 74378 reflections, $-16 \leq h \leq 16$, $-25 \leq k \leq 25$, $-18 \leq l \leq 18$, 1.823 ° < θ < 26.022 °, completeness 100 %, 8045 independent reflections, 6686 reflections observed with [I > 2σ(I)], 808 parameters, 1207 restraints, R indices (all data) R_1 = 0.1073, wR_2 = 0.1840; final R indices [I > 2σ(I)] R_1 = 0.0933, wR_2 = 0.1780, largest difference peak and hole 2.100 and −1.470 e $Å^{-3}$, Goof = 1.523.

Crystal Data for [(Dipp$_2$Im)$_2$Cu]FAP (5a): $C_{60}H_{72}CuF_{18}N_4P$, M_r = 1285.72, T = 100(2) K, λ = 1.54184 Å, colorless block, 0.161×0.193×0.483 mm^3, triclinic space group $P\bar{1}$, a = 12.56320(10) Å, b = 14.67720(10) Å, c = 17.51470(10) Å, α = 95.8630(10) °, β = 90.8740(10) °, γ = 102.9830(10) ° V = 3128.13(4) $Å^3$, Z = 2, ρ_{calcd} = 1.365 Mg/m^3, μ = 1.563 mm^{-1}, F(000) = 1332, 65545 reflections, $-15 \leq h \leq 15$, $-18 \leq k \leq 16$, $-21 \leq l \leq 21$, 2.538 ° < θ < 74.502 °, completeness 99.8 %, 12766 independent reflections, 11575 reflections observed with [I > 2σ(I)], 1145 parameters, 1662 restraints, R indices (all data) R_1 = 0.0454, wR_2 = 0.1109, final R indices [I > 2σ(I)] R_1

= 0.0418, wR_2 = 0.1083, largest difference peak and hole 0.625 and −0.399 e Å^{-3}, Goof = 1.048.

Crystal Data for [(Dipp$_2$Im)Cu(Me$_2$Im^{Me})]FAP (5c): $C_{40}H_{48}CuF_{18}N_4P$, M_r = 1021.33, T = 100(2) K, λ = 1.54184 Å, colorless block, 0.082×0.141×0.235 mm^3, monoclinic space group $P2_1/n$, a = 15.42160(10) Å, b = 17.00390(10) Å, c = 17.15540(10) Å, β = 90.8200(10) °, V = 4498.15(5) Å^3, Z = 4, ρ_{calcd} = 1.508 Mg/m^3, μ = 2.013 mm^{-1}, F(000) = 2088, 48398 reflections, −19 ≤ h ≤ 19, −21 ≤ k ≤ 20, −21 ≤ l ≤ 18, 3.660 ° < θ < 74.495 °, completeness 99.7 %, 9174 independent reflections, 8380 reflections observed with [I > 2σ(I)], 589 parameters, 0 restraints, R indices (all data) R_1 = 0.0461, wR_2 = 0.1146, final R indices [I > 2σ(I)] R_1 = 0.0428, wR_2 = 0.1120, largest difference peak and hole 1.319 and −0.658 e Å^{-3}, Goof = 1.034.

Crystal Data for [(Dipp$_2$Im)Cu(cAAC^{Me})]FAP (5d): $C_{53}H_{67}CuF_{18}N_3P$, M_r = 1182.60, T = 99(2) K, λ = 1.54184 Å, colorless block, 0.102×0.126×0.199 mm^3, monoclinic space group $P2_1/n$, a = 12.6055(2) Å, b = 22.9271(3) Å, c = 19.4891(2) Å, β = 93.7970(10) °, V = 5620.13(13) Å^3, Z = 4, ρ_{calcd} = 1.398 Mg/m^3, μ = 1.683 mm^{-1}, F(000) = 2448, 58992 reflections, −15 ≤ h ≤ 14, −27 ≤ k ≤ 28, −24 ≤ l ≤ 21, 2.980 ° < θ < 74.495 °, completeness 99.6 %, 11451 independent reflections, 10054 reflections observed with [I > 2σ(I)], 701 parameters, 0 restraints, R indices (all data) R_1 = 0.0399, wR_2 = 0.0818, final R indices [I > 2σ(I)] R_1 = 0.0332, wR_2 = 0.0790, largest difference peak and hole 0.559 and −0.393 e Å^{-3}, Goof = 1.053.

Additional Crystallographic Data

Crystal data for [(F)(Cp)$_2$Ti(μ-F)Ti(Cp)$_2$(F)]FAP (7): $C_{26}H_{20}F_{21}PTi_2$, M_r = 858.19, T = 100(2) K, λ = 1.54184 Å, orange plate, 0.130×0.147×0.252 mm^3, triclinic space group $P\bar{1}$, a = 10.8441(10) Å, b = 11.2239(8) Å, c = 13.2711(5) Å, α = 71.424(7) °, β = 73.752(7) °, γ = 86.620(7) °, V = 1469.2(2) Å^3, Z = 2, ρ_{calcd} = 1.840 Mg/m^3, μ = 6.601 mm^{-1}, F(000) = 848, 29067 reflections, −13 ≤ h ≤ 13, −10 ≤ k ≤ 14, −16 ≤ l ≤ 16, 3.655 ° < θ < 80.939 °, completeness 95.1 %, 6154 independent reflections, 5646 reflections observed with [I > 2σ(I)], 451 parameters, 0 restraints, R indices (all data) R_1 = 0.3076, wR_2 = 0.6617, final R indices [I > 2σ(I)] R_1 = 0.3034, wR_2 = 0.6524, largest difference peak and hole 10.508 and −2.518 e Å^{-3}, Goof = 3.683.

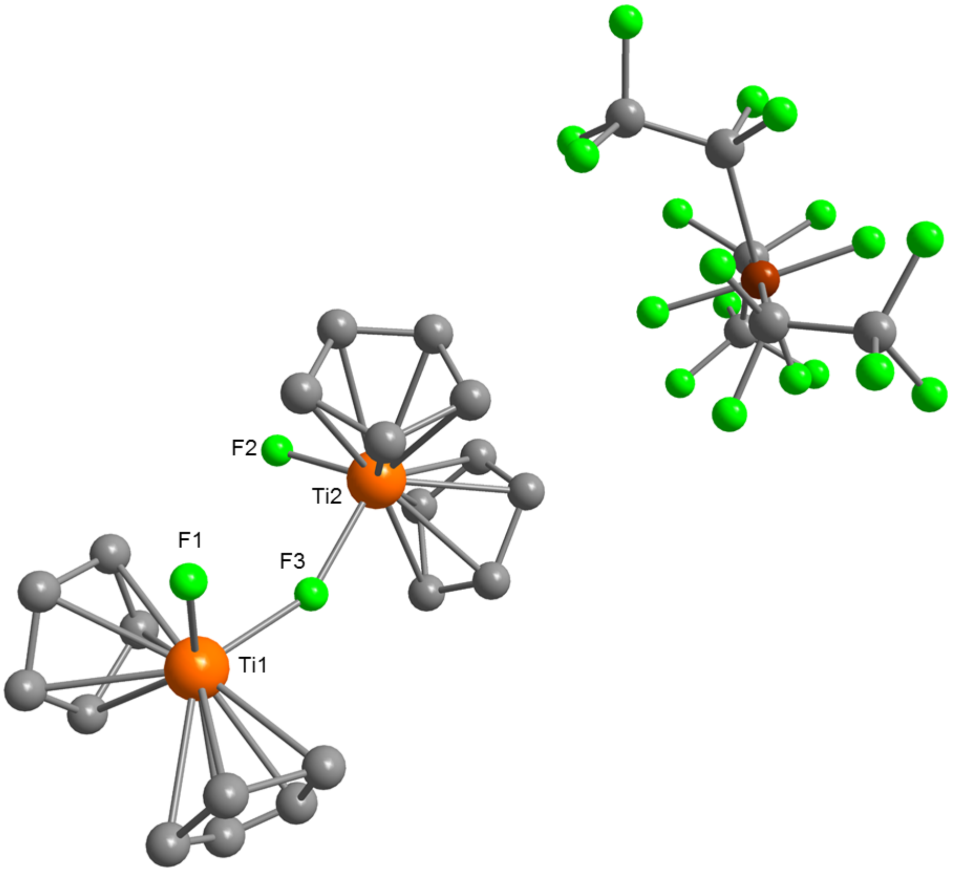

Figure S65: Molecular structure of [(F)(Cp)$_2$Ti(μ-F)Ti(Cp)$_2$(F)]**FAP** (**7**) in the solid state.

4) Additional Tables and Figures

Table S1: ^{19}F chemical shifts (ppm) of **2a-c[OEt$_2$]** in d$_8$-THF.

Compound	aryl-F_{ortho}/$F_{2,6}$	aryl-F_{meta}/$F_{3,5}$	aryl-F_{para}	aryl-CF_3/$F_{4'}$	aryl-$F_{2',6'}$	aryl-$F_{3',5'}$
**2a[OEt$_2$]**	−117.7	−165.1	−162.5	/	/	/
**2b[OEt$_2$]**	−116.8	−145.2	/	−56.8	/	/
**2c[OEt$_2$]**	−117.7	−143.1	/	−142.8	−163.6	−154.1

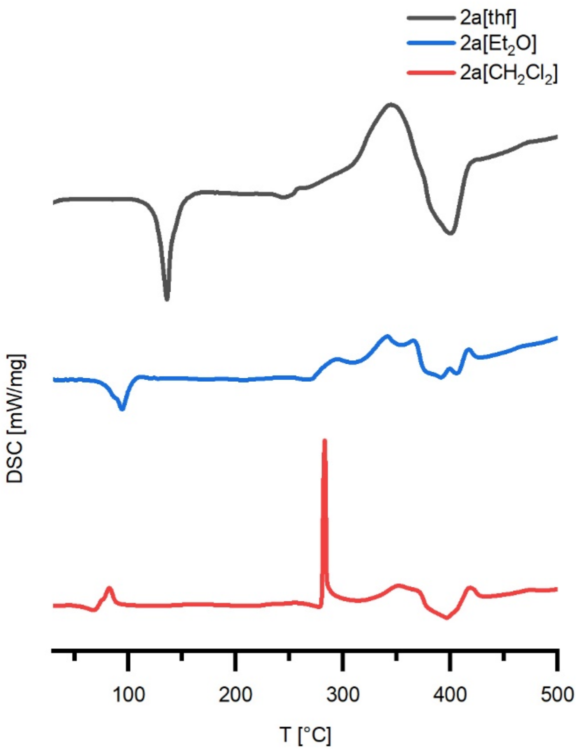

Figure S66: DSC curves (heat rate: 10 K/min) of **2a[thf]** (top), **2a[OEt$_2$]** (middle) and **2a[ClCH$_2$Cl]** (bottom).

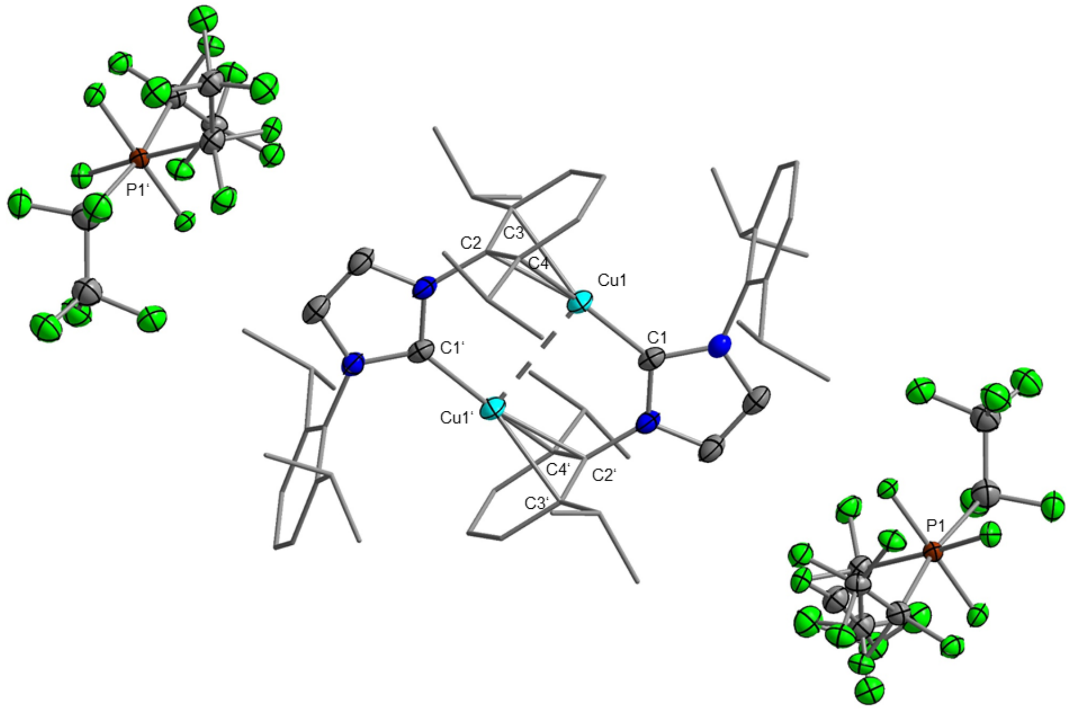

Figure S67: Molecular structure of [{(Dipp$_2$Im)Cu}$_2$]**FAP**$_2$ (**4**) in the solid state (ellipsoids set at the 50 % probability level for the cation and 20 % for the anions, respectively; only the major parts (81 %) of the disordered anions are shown; *i*Pr groups and phenyl groups are shown as wire-and-stick model). Hydrogen atoms and disordered CH_2Cl_2 solvent molecules were omitted for clarity.

4) Computational Details

Quantum chemical calculations were performed using the TURBOMOLE V7.3 program suite, a development of University of Karlsruhe and Forschungszentrum Karlsruhe GmbH, 1989-2007, TURBOMOLE GmbH, since 2007; available from http://www.turbomole.com.[S5] Geometries were optimized using (RI)-DFT calculations[S6] on an m4 grid employing the BP86[S7] or the PBE0[S8] functional and def2-SVP or def2-TZVP basis sets for all atoms.[S9] Frequency calculations with the AOFORCE[S10] module were performed at the same level to ensure that all structures represent true minima by the absence of imaginary frequencies. To calculate FIAs the approach of Christe[S11] and Krossing was used.[S12] The FIAs were calculated via isodesmic reactions involving the FIA of COF_2. For the metal complexes, single point calculations with the PBE0/def2-TZVP geometries were performed at the same level of theory including the COSMO solvation model[S13] with $\varepsilon_r = 9.08$ for CH_2Cl_2. Optimized cartesian coordinates are given in the Supporting Information.

The FIAs were calculated using the following equations.

$$OCF_2 + F^- \xrightarrow{\text{- 209 kJ/mol}} [OCF_3]^-$$

$$LA + OCF_3^- \xrightarrow{\text{calculated}} [LA\text{-}F]^- + OCF_2$$

$$LA + F^- \xrightarrow{\text{FIA}} [LA\text{-}F]^-$$

Cartesian Coordinates of Optimized Geometries

(coordinates in Å, energies in atomic units).

PF_5

NIMAG = 0

Energy = -840.2488902196

P	0.0000000	0.0000000	0.0000000
F	0.7956507	1.3781074	0.0000000
F	0.0000000	0.0000000	-1.6130762
F	0.7956507	-1.3781074	0.0000000

F	0.0000000	0.0000000	1.6130762
F	-1.5913014	0.0000000	0.0000000

$[PF_6]^-$

NIMAG = 0

Energy = -940.1537794896

P	-0.5808877	0.3198726	-1.8395133
F	-0.8195690	1.9555871	-1.8395133
F	-0.5808880	0.3198726	-3.4925677
F	1.0548268	0.5585538	-1.8395135
F	-0.5808875	0.3198726	-0.1864588
F	-0.3422065	-1.3158419	-1.8395133
F	-2.2166022	0.0811913	-1.8395130

AsF_5

NIMAG = 0

Energy = -2734.777412122

As	-0.0000000	0.0000000	0.0000000
F	-0.8601120	-1.4897576	0.0000000
F	0.0000000	0.0000000	-1.7370701
F	-0.8601120	1.4897576	0.0000000
F	0.0000000	0.0000000	1.7370701
F	1.7202239	0.0000000	0.0000000

$[AsF_6]^-$

NIMAG = 0

Energy = -2834.694701715

As	0.0000000	0.0000000	-0.0000005
F	-0.0000003	1.7742649	0.0000003
F	-1.2539579	-0.0000002	-1.2539576
F	1.2539571	0.0000002	1.2539574
F	0.0000003	-1.7742649	0.0000003

F 1.2539579 0.0000002 -1.2539576

F -1.2539571 -0.0000002 1.2539574

SbF_5

NIMAG = 0

Energy = -739.2665663251

Sb 0.0000000 0.0000000 0.0000000

F 0.0000000 0.0000000 1.9212983

F 0.9564274 -1.6565809 0.0000000

F 0.9564274 1.6565809 0.0000000

F 0.0000000 0.0000000 -1.9212983

F -1.9128548 0.0000000 0.0000000

$[SbF_6]^-$

NIMAG = 0

Energy = -839.2023264757

F -0.0000004 1.9517492 -0.0000000

F -1.3793483 -0.0000003 -1.3793486

F 1.3793483 0.0000003 1.3793486

F 0.0000004 -1.9517492 -0.0000000

F 1.3793483 0.0000003 -1.3793486

F -1.3793483 -0.0000003 1.3793486

Sb 0.0000000 0.0000000 -0.0000001

$(C_2F_5)_3PF_2$ (BP-86/def2-SV(P))

NIMAG = 0

Energy = -2265.881411945

P -0.0496309 0.0555378 -0.2888523

C -1.2802010 -1.4161177 -0.0200150

C -1.7922246 -1.7002155 1.4277751

C -1.0042483 1.6967337 -0.6721839

C	-0.2551814	2.9953221	-0.2204221
C	1.9031685	-0.0378333	-0.2872227
C	2.4342150	-1.5039192	-0.1934501
F	-0.0110163	-0.3792149	-1.8962983
F	-0.0405906	0.4598766	1.3301433
F	2.3933023	0.6497967	0.7624617
F	2.3626500	0.5138156	-1.4313015
F	3.7602116	-1.4920332	0.0016700
F	2.1618315	-2.1844677	-1.3125331
F	1.8439458	-2.1188230	0.8528303
F	-0.3015122	3.1586125	1.1034525
F	1.0364916	2.9120879	-0.6121195
F	-0.8150232	4.0618565	-0.8103238
F	-2.1842212	1.6278912	-0.0027757
F	-1.2509396	1.7983269	-1.9897613
F	-2.4613122	-0.6460748	1.9133621
F	-2.6290540	-2.7539026	1.3778551
F	-0.7702431	-2.0044058	2.2405848
F	-0.6904337	-2.5470871	-0.4766983
F	-2.3599843	-1.1457625	-0.7961772

$(C_2F_5)_3PF_2$ (PBE0/def2-TZVP)

NIMAG = 0

Energy = -2266.231150319

P	-0.0812809	0.0572317	-0.2683110
C	-1.2823339	-1.4204022	-0.0840468
C	-1.8140965	-1.7904293	1.3278383
C	-1.0075671	1.7103923	-0.5600563
C	-0.1497139	2.9866902	-0.3536571
C	1.8483711	-0.0129936	-0.2468792
C	2.4185549	-1.4415554	-0.0427008

F	-0.0376247	-0.2703784	-1.8477077
F	-0.0742961	0.3370184	1.3234117
F	2.3109836	0.7560118	0.7427050
F	2.3281478	0.4527804	-1.4032347
F	3.7327710	-1.3745056	0.0959978
F	2.1334216	-2.2070875	-1.0843885
F	1.8957841	-1.9850423	1.0515625
F	0.2639897	3.0895776	0.8980661
F	0.9091828	2.9396880	-1.1573585
F	-0.8730661	4.0526947	-0.6570513
F	-2.0324743	1.7531222	0.3042746
F	-1.4941670	1.7541477	-1.7997454
F	-2.4851477	-0.7831588	1.8622200
F	-2.6390231	-2.8236910	1.1953770
F	-0.8235049	-2.1444641	2.1310769
F	-0.6954678	-2.5140822	-0.5864154
F	-2.3514427	-1.1215645	-0.8409771

fac-**FAP** (BP-86/def2-SV(P))

NIMAG = 0

Energy = -2365.770269474

P	0.7110779	0.5570171	0.6833201
F	0.3769705	0.7476601	2.3077327
F	2.3302420	0.3312763	1.0124638
F	0.9795689	2.2101915	0.5698399
C	1.1858982	0.5940088	-1.3067492
C	0.6333535	-1.4493500	1.0030048
C	-1.2306531	0.8572523	0.3652971
C	-1.7835644	2.3133957	0.4577356
C	1.1246702	-0.5886557	-2.3306931
C	-0.6231027	-2.3755257	1.0665446

F	1.2389192	-1.6696081	2.2131498
F	1.4410214	-2.0376545	0.0549954
F	-0.2160498	-3.6766262	1.0699584
F	-1.3292871	-2.1914752	2.1977073
F	-1.4457568	-2.2274984	0.0116930
F	2.4719485	1.0555160	-1.4068551
F	0.3876432	1.5628454	-1.8700110
F	2.2215462	-1.3686676	-2.2716727
F	1.0772948	-0.0920262	-3.5985810
F	0.0340750	-1.3626052	-2.1732306
F	-1.9450845	0.1741994	1.3212111
F	-1.6493442	0.3695407	-0.8472086
F	-3.1451515	2.2712641	0.3467376
F	-1.3424216	3.1168611	-0.5216229
F	-1.5038137	2.8786643	1.6452331

fac-**FAP** (PBE0/def2-TZVP)

NIMAG = 0

Energy = -2366.159766382

P	0.7077874	0.5487317	0.6764297
F	0.3927892	0.7266067	2.2558948
F	2.2795939	0.3174003	0.9768554
F	0.9655543	2.1513491	0.5659668
C	1.1635012	0.5921231	-1.2996353
C	0.6195343	-1.4409846	0.9856918
C	-1.2185146	0.8479121	0.3696020
C	-1.7709856	2.2991132	0.4597162
C	1.1501880	-0.5758045	-2.3307881
C	-0.6331361	-2.3566331	1.0869779
F	1.2470967	-1.6691450	2.1678591
F	1.3871248	-2.0355846	0.0361510

F	-0.2312075	-3.6396330	1.1182582
F	-1.3184406	-2.1490345	2.2058618
F	-1.4526363	-2.2354516	0.0486716
F	2.4236001	1.0856755	-1.4004788
F	0.3573798	1.5297688	-1.8626358
F	2.2278221	-1.3483149	-2.2345819
F	1.1640282	-0.0672769	-3.5751503
F	0.0646684	-1.3370149	-2.2367257
F	-1.9238271	0.1875855	1.3245254
F	-1.6571828	0.3614763	-0.8171438
F	-3.1128350	2.2539248	0.3531110
F	-1.3392472	3.0955423	-0.5068919
F	-1.4926558	2.8576683	1.6324589

mer-**FAP** (BP-86/def2-SV(P))

NIMAG = 0

Energy = -2365.790259037

P	-0.0999575	0.3963528	-0.0385091
F	0.4509806	1.9675494	-0.1529818
F	-1.6918234	0.9384766	-0.1517053
F	-0.6102258	-1.1993882	0.0798018
C	-0.2937879	0.5713890	1.9261245
C	1.8300994	-0.1379201	0.0877598
C	2.2844231	-1.6251813	0.2350597
C	-0.1310527	0.2301645	-2.0132596
C	-1.5249039	0.1207169	-2.7127845
F	2.3987310	0.5110461	1.1581643
F	2.4893355	0.3217239	-1.0277579
F	1.9208444	-2.3909459	-0.8073658
F	1.8277227	-2.2057660	1.3573795
F	3.6488175	-1.6608298	0.2968303
F	0.5357800	-0.9036749	-2.4077623

F	0.4769568	1.2918450	-2.6293932
F	-2.3013896	-0.8403257	-2.1838281
F	-1.3326120	-0.2031991	-4.0260152
F	-2.1979972	1.2857028	-2.7023202
F	0.3341035	-0.4712162	2.5623545
F	0.2655313	1.7290585	2.3987026
C	-1.7406781	0.5684071	2.5183975
F	-2.4058298	1.7080151	2.2551514
F	-1.6577541	0.4739412	3.8787305
F	-2.4753135	-0.4759417	2.0992267

mer-**FAP** (PBE0/def2-TZVP)

NIMAG = 0

Energy = -2366.180414692

P	-0.1096969	0.3815497	-0.0374420
F	0.4053964	1.9137880	-0.1491915
F	-1.6568159	0.8803749	-0.1446611
F	-0.5842470	-1.1743809	0.0780615
C	-0.2913141	0.5537952	1.9122656
C	1.8032850	-0.1339328	0.0863062
C	2.2790997	-1.6097502	0.2336202
C	-0.1297960	0.2159586	-1.9961492
C	-1.5137310	0.1326695	-2.7016633
F	2.3673596	0.5089519	1.1415871
F	2.4563022	0.3221902	-1.0139924
F	1.9277270	-2.3727207	-0.7929510
F	1.8427167	-2.1869961	1.3453412
F	3.6243712	-1.6208499	0.2881663
F	0.5087408	-0.9139215	-2.3934029
F	0.4874361	1.2531873	-2.6137719
F	-2.2886298	-0.8230637	-2.2020797

F	-1.3218509	-0.1580258	-4.0020373
F	-2.1733336	1.2862330	-2.6662324
F	0.3070727	-0.4854430	2.5478942
F	0.2778105	1.6871195	2.3917751
C	-1.7290044	0.5789832	2.5058396
F	-2.3770707	1.7042732	2.2215209
F	-1.6464895	0.5139693	3.8479043
F	-2.4653379	-0.4539588	2.1132927

CH_2Cl_2

NIMAG = 0

Energy = -959.3960816847

C	-0.0835981	0.0591092	-0.1023966
H	0.2957137	0.5677219	-0.9833303
H	-1.1690738	0.0498596	-0.0863228
Cl	0.4784934	0.9341141	1.3206814
Cl	0.4784647	-1.6108048	-0.1486318

***trans*-[Ni(*i*Pr$_2$Im)$_2$(F)(C$_6$F$_5$)]**

NIMAG = 0

Energy = -3258.765451028

Ni	0.1370274	-0.2590244	0.1584146
N	-2.5413034	-0.6297356	1.3856888
N	-2.0184881	-2.2210676	0.0610851
C	-1.5798010	-1.0376726	0.5323647
C	-3.5638544	-1.5499340	1.4535262
H	-4.4313549	-1.4193554	2.0768393
C	-3.2317480	-2.5548098	0.6168154
H	-3.7581626	-3.4617041	0.3754219
C	-0.6527539	1.2160395	-0.7688465
C	-1.3100859	1.0791940	-1.9793522

C	-1.8915047	2.1281720	-2.6735619
C	-1.8297124	3.4054206	-2.1429801
F	-1.4017792	-0.1358561	-2.5605082
F	-2.5041231	1.9289501	-3.8395304
F	-2.3811035	4.4302316	-2.7859455
C	-0.6185477	2.5131681	-0.2867709
C	-1.1854190	3.6000765	-0.9331578
F	-0.0143118	2.7763981	0.8912794
F	-1.1232113	4.8237575	-0.4104309
N	2.6310685	0.4721831	-1.2821370
N	2.7696027	0.8029418	0.8229955
C	1.9154298	0.4067405	-0.1406941
C	3.9178188	0.8962190	-1.0344317
H	4.6558901	1.0168318	-1.8083004
C	4.0038532	1.1039883	0.2958066
H	4.8316464	1.4408444	0.8951386
C	-1.2527225	-3.0700933	-0.8519217
H	-0.4371879	-2.4290794	-1.1916146
C	-2.0971474	-3.4840681	-2.0440509
C	-0.6557146	-4.2475957	-0.1009065
H	-0.0179619	-3.8770038	0.7023402
H	-1.4377616	-4.8905132	0.3137532
H	-0.0512561	-4.8520464	-0.7809403
H	-2.9156860	-4.1466172	-1.7497029
H	-1.4771000	-4.0311982	-2.7566444
H	-2.5168018	-2.6135363	-2.5503278
C	-2.4666429	0.5928554	2.1804547
H	-1.5380045	1.0666062	1.8599406
C	-2.3613828	0.2630895	3.6605887
C	-3.6282759	1.5199244	1.8673404
H	-3.6734907	1.7397686	0.7994735

H	-4.5834672	1.0839419	2.1723419
H	-3.5057714	2.4613022	2.4060925
H	-3.2693099	-0.2229639	4.0276814
H	-2.2189024	1.1801700	4.2355145
H	-1.5155491	-0.4004135	3.8489326
C	2.1210657	0.0822285	-2.5936214
H	1.0819884	-0.1923738	-2.4083953
C	2.8632223	-1.1371828	-3.1158252
C	2.1586436	1.2509175	-3.5630959
H	1.6140752	2.1068901	-3.1612405
H	3.1848159	1.5620896	-3.7767562
H	1.6950345	0.9627701	-4.5083578
H	3.9153881	-0.9124923	-3.3103046
H	2.4151334	-1.4697829	-4.0541445
H	2.8134617	-1.9568564	-2.3970220
C	2.4397993	0.8238749	2.2483506
H	1.3592453	0.6721390	2.2745925
C	2.7744317	2.1719957	2.8619901
C	3.1039056	-0.3415902	2.9606836
H	2.7747249	-1.2766304	2.5062510
H	4.1942485	-0.2682801	2.9093112
H	2.8179406	-0.3422447	4.0148942
H	3.8524767	2.3547084	2.8742734
H	2.4292833	2.1963904	3.8972853
H	2.2887339	2.9834638	2.3179231
F	0.8974462	-1.6845606	1.0561349

***trans*-[Ni(*i*Pr$_2$Im)$_2$(C$_6$F$_5$)]$^+$**

NIMAG = 0

Energy = -3158.738002401

Ni	-0.0000954	-0.3535575	-0.0002543

N	-2.6944130	-0.2181878	1.2270996
N	-2.7526927	-0.9319082	-0.7921029
C	-1.9078269	-0.4873343	0.1641142
C	-4.0063366	-0.4917678	0.9413837
H	-4.8035517	-0.3481185	1.6501575
C	-4.0433700	-0.9454031	-0.3323258
H	-4.8775989	-1.2699428	-0.9297723
C	-0.0000420	1.5126473	-0.0006047
C	-0.5049774	2.2434718	-1.0603612
C	-0.5121319	3.6293807	-1.0825409
C	0.0003484	4.3291377	-0.0012937
F	-1.0066556	1.6129199	-2.1345785
F	-0.9999021	4.2853838	-2.1232104
F	0.0005481	5.6489977	-0.0016239
C	0.5050711	2.2438526	1.0588045
C	0.5126180	3.6297695	1.0803041
F	1.0066198	1.6136884	2.1333109
F	1.0006039	4.2861447	2.1206376
N	2.6948161	-0.2179451	-1.2266808
N	2.7520883	-0.9318981	0.7924653
C	1.9076794	-0.4873655	-0.1641802
C	4.0066336	-0.4912937	-0.9402404
H	4.8042247	-0.3474124	-1.6485442
C	4.0430314	-0.9450807	0.3334329
H	4.8769786	-1.2695444	0.9313139
C	-2.3338401	-1.3905065	-2.1220811
H	-1.2905809	-1.0694359	-2.2021813
C	-3.1382520	-0.7075377	-3.2129105
C	-2.4037201	-2.9063059	-2.2093501
H	-1.8088195	-3.3802470	-1.4260701
H	-3.4334936	-3.2588054	-2.1158030

H	-2.0258973	-3.2398625	-3.1771145
H	-4.1844896	-1.0211974	-3.1954692
H	-2.7343959	-0.9836828	-4.1879936
H	-3.0948626	0.3774940	-3.1176898
C	-2.2190969	0.2980977	2.5163420
H	-1.1381590	0.3856032	2.3939673
C	-2.5134811	-0.6918722	3.6305057
C	-2.7982720	1.6750696	2.7894621
H	-2.5814056	2.3638497	1.9716045
H	-3.8810853	1.6329369	2.9288712
H	-2.3634423	2.0806597	3.7041465
H	-3.5877701	-0.8072863	3.7913827
H	-2.0792342	-0.3311219	4.5640896
H	-2.0922887	-1.6750083	3.4120114
C	2.2201384	0.2982710	-2.5161894
H	1.1390954	0.3854238	-2.3944921
C	2.5155322	-0.6915651	-3.6302071
C	2.7990767	1.6754260	-2.7888839
H	2.5814124	2.3641338	-1.9711781
H	3.8820029	1.6336292	-2.9275156
H	2.3647792	2.0808846	-3.7038796
H	3.5899590	-0.8066415	-3.7904036
H	2.0817692	-0.3309135	-4.5640538
H	2.0944962	-1.6748352	-3.4120190
C	2.3325314	-1.3908272	2.1221001
H	1.2891003	-1.0701212	2.2015596
C	3.1359900	-0.7077809	3.2135822
C	2.4028498	-2.9066189	2.2091787
H	1.8087864	-3.3806348	1.4253100
H	3.4328269	-3.2587395	2.1164513
H	2.0243247	-3.2404720	3.1765666

H	4.1823539	-1.0210523	3.1967363
H	2.7316214	-0.9842936	4.1883496
H	3.0922730	0.3772520	3.1185580

***trans*-[Ni(*i*Pr$_2$Im)$_2$(ClCH$_2$Cl)(C$_6$F$_5$)]$^+$**

NIMAG = 0

Energy = -4118.151489823

Ni	0.0857158	-0.3003415	-0.0200007
N	-2.6411127	-0.2244737	1.1495793
N	-2.7081971	-0.6413163	-0.9500547
C	-1.8513404	-0.3848925	0.0659116
C	-3.9662344	-0.3722799	0.8176161
H	-4.7633198	-0.2756636	1.5341993
C	-4.0091628	-0.6359524	-0.5042613
H	-4.8508870	-0.8103822	-1.1515723
C	0.0061551	1.5950325	0.0596296
C	-0.3867600	2.3810052	-1.0085768
C	-0.4404807	3.7654274	-0.9670794
C	-0.0901094	4.4240036	0.1998531
F	-0.7408428	1.8075689	-2.1737707
F	-0.8222142	4.4587957	-2.0293242
F	-0.1350634	5.7421194	0.2649827
C	0.3452968	2.2968007	1.2025768
C	0.3061288	3.6787147	1.2980798
F	0.7290301	1.6339793	2.3088879
F	0.6386767	4.2878434	2.4263592
N	2.7345808	0.1121153	-1.2610174
N	2.9491469	-0.4037647	0.8057136
C	2.0235132	-0.1897776	-0.1545580
C	4.0820701	0.0939695	-0.9968140
H	4.8280007	0.3112728	-1.7414076

C	4.2171216	-0.2307453	0.3052304
H	5.1029948	-0.3474929	0.9046912
C	-2.3335426	-0.8913692	-2.3486354
H	-1.2648218	-0.6717326	-2.3911871
C	-3.0724506	0.0517384	-3.2831630
C	-2.5689662	-2.3453068	-2.7240006
H	-1.9921799	-3.0256977	-2.0962480
H	-3.6256274	-2.6096266	-2.6405495
H	-2.2680935	-2.5108589	-3.7596538
H	-4.1399158	-0.1775162	-3.3188587
H	-2.6827090	-0.0630026	-4.2957097
H	-2.9453285	1.0911589	-2.9844956
C	-2.1747040	0.1138622	2.5004059
H	-1.0868216	0.1250200	2.4215025
C	-2.5929001	-0.9491210	3.5015724
C	-2.6588282	1.4979326	2.8984120
H	-2.3792102	2.2448906	2.1546532
H	-3.7446090	1.5192133	3.0189074
H	-2.2140181	1.7814728	3.8532944
H	-3.6791268	-0.9938404	3.6094891
H	-2.1782829	-0.7071844	4.4813586
H	-2.2317529	-1.9382440	3.2155384
C	2.1641068	0.3840973	-2.5837432
H	1.0849519	0.3705491	-2.4249378
C	2.5373463	-0.7174845	-3.5621418
C	2.5699326	1.7617234	-3.0764681
H	2.3161977	2.5330903	-2.3480916
H	3.6423848	1.8155634	-3.2777417
H	2.0484762	1.9859929	-4.0079910
H	3.6147536	-0.7443438	-3.7406314
H	2.0492938	-0.5386813	-4.5216295

H	2.2275958	-1.6982155	-3.1955450
C	2.6773143	-0.8274464	2.1856895
H	1.5916729	-0.7654390	2.2858262
C	3.3249140	0.1186710	3.1823329
C	3.1277628	-2.2638778	2.3997236
H	2.6614797	-2.9446276	1.6870212
H	4.2122674	-2.3536392	2.3022788
H	2.8594540	-2.5861231	3.4071535
H	4.4144716	0.0468200	3.1478803
H	3.0146964	-0.1520460	4.1927107
H	3.0350694	1.1527131	3.0015281
Cl	0.3710129	-2.6027947	-0.1958651
C	-0.9339237	-3.6258266	0.4928440
H	-1.8451549	-3.0361165	0.4721258
H	-0.9943443	-4.5020482	-0.1462973
Cl	-0.5805498	-4.1238647	2.1264630

[(Dipp$_2$Im)CuF]

NIMAG = 0

Energy = -2899.214281886

C	0.1618386	-0.1194798	0.0335573
C	-1.6320372	1.2198511	0.3366916
C	-1.4771619	1.0481992	-0.9934290
N	-0.3808879	0.2307432	-1.1576425
N	-0.6256904	0.5018130	0.9444344
C	-0.4379401	0.4238470	2.3627429
C	0.3801255	1.3730521	2.9838505
C	-1.0856881	-0.5944329	3.0694839
C	0.5319892	1.2838940	4.3634025
C	-0.8992973	-0.6373577	4.4469826
C	-0.1019232	0.2921641	5.0895690

H	1.1651129	1.9995065	4.8753633
H	-1.3809456	-1.4184227	5.0240616
H	0.0332313	0.2382273	6.1638321
C	0.1237273	-0.1949816	-2.4294120
C	1.0825069	0.5972971	-3.0690740
C	-0.3648797	-1.3837673	-2.9808500
C	1.5452013	0.1683133	-4.3084214
C	0.1319623	-1.7661726	-4.2222167
C	1.0744596	-0.9987635	-4.8820929
H	2.2943794	0.7548452	-4.8277434
H	-0.2196174	-2.6864996	-4.6743669
H	1.4519483	-1.3175458	-5.8470131
C	1.6459065	1.8571264	-2.4495817
H	1.1042606	2.0491640	-1.5204685
C	-1.3667435	-2.2641807	-2.2671005
H	-1.6707793	-1.7558856	-1.3491569
C	1.4486391	3.0726260	-3.3521434
H	0.3947223	3.2242348	-3.5969627
H	1.8160938	3.9747399	-2.8567566
H	1.9969043	2.9674891	-4.2915663
C	3.1188530	1.6692810	-2.0884976
H	3.5063107	2.5638637	-1.5939695
H	3.2589047	0.8184071	-1.4181514
H	3.7232363	1.4935366	-2.9821760
C	-2.6244889	-2.4874499	-3.1034440
H	-3.3505270	-3.0815023	-2.5429147
H	-3.0977376	-1.5415959	-3.3773925
H	-2.4008924	-3.0276342	-4.0266975
C	-0.7280310	-3.5922728	-1.8628895
H	0.1553081	-3.4358407	-1.2397674
H	-1.4409537	-4.2017429	-1.3015107

H	-0.4197207	-4.1640794	-2.7420771
C	1.1150225	2.4475403	2.2133392
H	0.8118902	2.3806051	1.1658907
C	2.6243844	2.2152826	2.2669538
H	3.1459605	2.9648828	1.6662202
H	2.9977357	2.2899697	3.2916265
H	2.8883868	1.2261824	1.8863233
C	0.7530890	3.8471348	2.7047320
H	-0.3230875	4.0267368	2.6480123
H	1.0618577	4.0000455	3.7418185
H	1.2567583	4.6030018	2.0971253
C	-1.9348587	-1.6466381	2.3912726
H	-2.0023233	-1.3952482	1.3303457
C	-1.2760783	-3.0216360	2.4931107
H	-0.2700713	-3.0130998	2.0680454
H	-1.1964548	-3.3434650	3.5347573
H	-1.8688313	-3.7678182	1.9576549
C	-3.3573457	-1.6726041	2.9455045
H	-3.8412339	-0.6972360	2.8553622
H	-3.9613597	-2.4035689	2.4023034
H	-3.3709246	-1.9546488	4.0011625
H	-2.0400164	1.4287955	-1.8283306
H	-2.3578101	1.7807485	0.9000242
Cu	1.6450061	-1.2047011	0.3308351
F	3.0606252	-2.2428756	0.5974253

[(Dipp$_2$Im)Cu]$^+$

NIMAG = 0

Energy = -2799.170761254

C	0.2403447	0.0178806	0.0158967
C	-1.9181015	0.5454341	0.3190481

C	-1.7178216	0.3822309	-1.0076821
N	-0.3899474	0.0585919	-1.1710117
N	-0.7076941	0.3123495	0.9328500
C	-0.4889165	0.3199580	2.3526748
C	0.3685246	1.2779075	2.9170080
C	-1.1413849	-0.6488301	3.1271145
C	0.5418064	1.2547951	4.2961683
C	-0.9419656	-0.6105993	4.5037697
C	-0.1138073	0.3287976	5.0867024
H	1.1941984	1.9833986	4.7630316
H	-1.4389531	-1.3428000	5.1290336
H	0.0271582	0.3367322	6.1609578
C	0.2380317	-0.2123816	-2.4348534
C	0.9536291	0.8135358	-3.0624088
C	0.0997172	-1.4919132	-2.9828217
C	1.5534479	0.5188031	-4.2817565
C	0.7150980	-1.7272715	-4.2082539
C	1.4348243	-0.7367711	-4.8508587
H	2.1172244	1.2864373	-4.7985547
H	0.6302100	-2.7062989	-4.6650663
H	1.9057893	-0.9436210	-5.8046850
C	1.1122688	2.1908450	-2.4553243
H	0.4362282	2.2671850	-1.5993958
C	-0.6652996	-2.6054990	-2.3008933
H	-1.0695653	-2.2209123	-1.3611635
C	0.7308510	3.3038911	-3.4284743
H	-0.2857133	3.1716512	-3.8030155
H	0.7861140	4.2752764	-2.9322681
H	1.4022415	3.3379494	-4.2888513
C	2.5374422	2.3897757	-1.9423534
H	2.6492119	3.3615456	-1.4557305

H	2.8140643	1.6099779	-1.2158363
H	3.2652565	2.3309588	-2.7546562
C	-1.8465295	-3.0718393	-3.1500593
H	-2.4132471	-3.8408599	-2.6206278
H	-2.5260883	-2.2491286	-3.3833125
H	-1.5111636	-3.5022552	-4.0963030
C	0.2554279	-3.7741450	-1.9530734
H	1.0859896	-3.4546545	-1.3194990
H	-0.3006290	-4.5488077	-1.4203764
H	0.6777368	-4.2296206	-2.8515978
C	1.1325260	2.3010520	2.1013654
H	0.7643805	2.2678287	1.0733732
C	2.6334334	1.9786408	2.0832599
H	3.1707956	2.5634010	1.3344546
H	3.0933233	2.1807163	3.0546498
H	2.8749858	0.8904472	1.9585295
C	0.9228393	3.7311806	2.5957088
H	-0.1363864	3.9921060	2.5893020
H	1.2966738	3.8695705	3.6119222
H	1.4503385	4.4382026	1.9514076
C	-2.0122027	-1.7427622	2.5440001
H	-2.0039448	-1.6533181	1.4555989
C	-1.4555096	-3.1259976	2.8799688
H	-0.4251759	-3.2378751	2.5355839
H	-1.4714859	-3.3158494	3.9553057
H	-2.0600697	-3.8994294	2.4012305
C	-3.4619251	-1.6109610	3.0078495
H	-3.8861123	-0.6370670	2.7523796
H	-4.0802825	-2.3823461	2.5440810
H	-3.5432006	-1.7291091	4.0908233
H	-2.3942620	0.4652324	-1.8411462

H	-2.8052515	0.7992603	0.8734493
Cu	2.1005036	0.0993768	0.4434117

$[(Dipp_2Im)Cu]_2^{2+}$

NIMAG = 0

Energy = -5598.363296735

Cu	-0.6498087	-1.5943235	-1.3614124
Cu	0.5686146	1.6371536	1.3088005
C	1.0250368	-1.4529110	-2.3051592
C	-2.1110991	-0.5929361	1.4231268
C	-1.6424466	-1.8535546	1.8422565
C	-2.6698378	-0.3686114	0.1630242
C	-1.0508521	1.4367774	2.3330348
C	2.0601301	0.6256840	-1.4790997
C	1.6145616	1.8416807	-2.0302396
C	2.5596259	0.5159873	-0.1766292
N	2.0343487	-0.5494195	-2.2974784
N	1.4047478	-2.3748875	-3.2117871
N	-2.0401979	0.5117289	2.3307624
C	-1.6945411	-2.8949271	0.9232650
C	-1.1584232	-2.1083834	3.2518930
C	-2.7051612	-1.4520217	-0.7360479
C	-3.3149152	0.9389515	-0.2317044
N	-1.4070880	2.3085440	3.2970600
C	1.5843331	2.9533101	-1.1944709
C	1.2665125	1.9810807	-3.4958850
C	2.5521579	1.6778106	0.6198118
C	3.2066418	-0.7471648	0.3424973
C	3.0242108	-0.9096613	-3.1870444
C	2.6226915	-2.0629809	-3.7641400
C	0.6205586	-3.5355597	-3.5417643

C	-3.0016502	0.8150141	3.2707520
H	-1.3497940	-3.8794201	1.2117409
C	-2.2217432	-2.7112383	-0.3555085
H	-0.8119071	-1.1557005	3.6628614
C	-2.3195099	-2.6054321	4.1203877
C	-0.0020002	-3.1006223	3.3251911
H	-3.2302056	-1.3414187	-1.6789062
H	-3.1555907	1.6529530	0.5782080
C	-4.8252168	0.7585430	-0.3984716
C	-2.6857009	1.5282399	-1.4887218
C	-2.5961892	1.9493989	3.8824583
C	-0.6328224	3.4697710	3.6492100
H	1.2339093	3.9020940	-1.5803138
C	2.0420416	2.8840840	0.1175864
H	1.0153988	0.9882669	-3.8787272
C	2.4970895	2.4863920	-4.2587595
C	0.0802170	2.8984721	-3.7706048
H	3.0581728	1.6682645	1.5800984
H	2.7679697	-1.5922873	-0.1939976
C	4.7087407	-0.7184433	0.0375503
C	2.9823353	-0.9718994	1.8327753
H	3.9155436	-0.3244838	-3.3318485
H	3.0929305	-2.6804985	-4.5107080
C	0.8891773	-4.7330768	-2.8680364
C	-0.3678149	-3.4136029	-4.5257283
H	-3.8832652	0.2142463	3.4130205
H	-2.3352363	-3.5617495	-1.0208498
H	-1.9790187	-2.7591069	5.1460216
H	-3.1533117	-1.9029625	4.1465422
H	-2.6980430	-3.5596918	3.7462703
H	-0.3249389	-4.1186845	3.0965189

H	0.8113385	-2.8405904	2.6461807
H	0.3986903	-3.1194745	4.3400509
H	-5.2876269	0.3555521	0.5046086
H	-5.2925174	1.7208936	-0.6149491
H	-5.0615843	0.0848092	-1.2253993
H	-3.1414697	2.4929435	-1.7204304
H	-1.6129361	1.6837282	-1.3521168
H	-2.8314067	0.8819696	-2.3582443
H	-3.0470666	2.5256610	4.6726872
C	-0.9163752	4.6789719	3.0028744
C	0.3572127	3.3376967	4.6300147
H	2.0912830	3.7846972	0.7211679
H	2.2788963	2.5371036	-5.3270230
H	3.3671207	1.8423478	-4.1232496
H	2.7716003	3.4897516	-3.9245005
H	0.2999711	3.9386719	-3.5207586
H	-0.8130906	2.5956305	-3.2242816
H	-0.1547724	2.8752480	-4.8360691
H	4.9120962	-0.6258695	-1.0299111
H	5.1786534	-1.6385855	0.3897179
H	5.1919185	0.1194774	0.5461060
H	3.3347207	-1.9659842	2.1130173
H	1.9256564	-0.8976107	2.1023240
H	3.5399063	-0.2586947	2.4439998
C	0.0943441	-5.8297903	-3.1856192
C	2.0077346	-4.8818461	-1.8586146
C	-1.1336583	-4.5432330	-4.7971298
C	-0.6071064	-2.1383383	-5.3058211
C	-0.1365001	5.7784476	3.3470118
C	-2.0360646	4.8377879	1.9963149
C	1.1048851	4.4717472	4.9315286

C	0.6133395	2.0478564	5.3797714
C	-0.9107182	-5.7359762	-4.1322720
H	0.2733209	-6.7775885	-2.6914711
H	2.4851805	-3.9068983	-1.7273007
C	3.0762717	-5.8486774	-2.3689269
C	1.4859382	-5.3193946	-0.4919550
H	-1.9075191	-4.4921914	-5.5540840
H	0.1183748	-1.3907817	-4.9736450
C	-0.3826659	-2.3556865	-6.8018046
C	-2.0002071	-1.5719297	-5.0405645
C	0.8664675	5.6767464	4.2949420
H	-0.3271519	6.7348766	2.8742938
H	-2.5063462	3.8618453	1.8478839
C	-3.1117863	5.7875887	2.5230170
C	-1.5165670	5.3031795	0.6378987
H	1.8781247	4.4133934	5.6885612
H	-0.0594825	1.2810285	4.9860016
C	0.3008226	2.2090105	6.8673647
C	2.0419901	1.5497133	5.1744963
H	-1.5138991	-6.6052684	-4.3667648
H	2.6721151	-6.8556385	-2.4933144
H	3.9048040	-5.9097175	-1.6600632
H	3.4777503	-5.5333201	-3.3342021
H	1.0067491	-6.2995898	-0.5404344
H	0.7562333	-4.6071587	-0.0981538
H	2.3091507	-5.3937076	0.2222079
H	-1.1042921	-3.0629655	-7.2157698
H	0.6168569	-2.7448409	-7.0051294
H	-0.4972750	-1.4140963	-7.3429752
H	-2.7811678	-2.2699517	-5.3500070
H	-2.1481970	-0.6430146	-5.5957468

H	-2.1451090	-1.3582788	-3.9769508
H	1.4565446	6.5486850	4.5518419
H	-2.7155281	6.7953413	2.6649260
H	-3.9406856	5.8547597	1.8150929
H	-3.5108633	5.4520519	3.4824140
H	-1.0428600	6.2849633	0.7050762
H	-0.7825482	4.6027435	0.2305151
H	-2.3399443	5.3863311	-0.0751563
H	0.9623652	2.9414896	7.3348337
H	-0.7266712	2.5410662	7.0290483
H	0.4372829	1.2596523	7.3895786
H	2.7743351	2.2608708	5.5622733
H	2.1945431	0.6032276	5.6977617
H	2.2597033	1.3903457	4.1147241

Cp_2TiF_2

NIMAG = 0

Energy = -1435.776083916

Ti	0.3504808	0.5095117	0.0597165
F	0.2807937	1.8621185	1.2753018
F	1.7584069	1.0985361	-0.9310368
C	0.9756083	-1.7894952	0.1249888
C	0.4652512	-0.7589033	2.0943901
C	-1.9961213	0.3010887	-0.2997540
C	1.7940425	-0.4227261	1.7505991
C	-0.8558288	1.7918907	-1.5873957
C	-0.6444425	0.5026146	-2.1256031
C	-1.7052265	1.6657152	-0.4791344
C	-0.0390716	-1.6081672	1.0991485
C	-1.3524242	-0.4175684	-1.3394522
C	2.1107578	-1.0762084	0.5515035

H	-1.0267318	-2.0414864	1.0748565
H	-0.0825961	-0.3747265	2.9413591
H	2.4245228	0.2749524	2.2796759
H	3.0274982	-0.9619596	-0.0050886
H	0.9002060	-2.3824100	-0.7739253
H	0.0026001	0.2652932	-2.9563141
H	-0.3840557	2.7012759	-1.9260252
H	-1.9948846	2.4634038	0.1865550
H	-2.6139833	-0.1183279	0.4797066
H	-1.3948019	-1.4844219	-1.4940721

$[Cp_2TiF]^+$

NIMAG = 0

Energy = -1335.687645836

Ti	0.1218607	0.4943831	0.2705063
F	0.5056530	2.0244000	1.0985170
C	0.8979969	-1.7209379	0.0978311
C	0.7742307	-0.7151071	2.1500429
C	-2.1041151	0.5327439	-0.4325371
C	1.9479888	-0.2689142	1.5247922
C	-0.5305813	1.6639479	-1.6334839
C	-0.4382283	0.2863642	-1.9718148
C	-1.5586145	1.8146561	-0.6909305
C	0.1098879	-1.6045759	1.2619631
C	-1.4342966	-0.4034355	-1.2490325
C	2.0150099	-0.8707599	0.2436667
H	-0.8136601	-2.1294686	1.4612628
H	0.4188521	-0.4014611	3.1221782
H	2.6386336	0.4598943	1.9222106
H	2.7965755	-0.7181077	-0.4889562
H	0.6816597	-2.3368005	-0.7614475

H	0.2463222	-0.1477986	-2.6866923
H	0.1069766	2.4553720	-2.0035087
H	-1.8342897	2.7346711	-0.1972351
H	-2.9063397	0.3123104	0.2594036
H	-1.6415224	-1.4613760	-1.2967358

[(F)(Cp)$_2$Ti(μ-F)Ti(Cp)$_2$(F)]$^+$

NIMAG = 0

Energy = -2771.524480248

Ti	-1.4350069	-1.0785044	0.8144996
F	-1.7854334	0.3561203	1.8686266
F	0.1588449	-0.1618670	0.0043613
C	-1.0916055	-3.3796935	1.3103040
C	-0.8434197	-1.8597099	3.0000028
C	-3.3960866	-2.0353498	-0.0457175
C	0.3240073	-1.8468477	2.2306969
C	-2.8597865	0.0801561	-0.7004171
C	-2.0954479	-0.8184127	-1.4682024
C	-3.6726817	-0.6715915	0.1688493
C	-1.7439043	-2.7734137	2.4132601
C	-2.4109009	-2.1263007	-1.0620684
C	0.1707943	-2.7893961	1.1798846
Ti	1.1324019	1.3846584	-0.8305502
F	-0.2453306	1.6984660	-1.9687925
C	2.0655100	3.3766594	-0.0166417
C	2.0115339	0.6845670	-2.9456424
C	2.1015743	2.4386041	1.0466901
C	0.7739675	2.1501535	1.4057649
C	1.9492173	-0.4451485	-2.1242886
C	0.7153341	3.6517920	-0.3061219
C	3.4511534	0.9993151	-1.1976982
C	2.8417184	-0.2505195	-1.0372551

C	2.9056097	1.6039701	-2.3579415
C	-0.0821841	2.8840550	0.5632194
H	-2.7341413	-3.0063101	2.7754548
H	-1.0603735	-1.2144223	3.8369150
H	1.1817833	-1.2117634	2.3905062
H	0.8971352	-3.0105160	0.4114661
H	-1.5020526	-4.1510982	0.6781533
H	-1.3622392	-0.5316000	-2.2057808
H	-2.7990054	1.1558898	-0.7498450
H	-4.3449934	-0.2701790	0.9123608
H	-3.8714074	-2.8649675	0.4542273
H	-1.9986216	-3.0361692	-1.4706562
H	1.4095866	0.8672430	-3.8221114
H	1.3132196	-1.3040136	-2.2744022
H	3.0178506	-0.9416666	-0.2259596
H	4.1956473	1.4325969	-0.5487621
H	3.1661272	2.5741018	-2.7541292
H	0.4476418	1.4536763	2.1622960
H	-1.1594859	2.8321705	0.5659107
H	0.3540603	4.2918938	-1.0971103
H	2.9211667	3.8235239	-0.4986715
H	2.9882230	2.0398480	1.5153161

References

[S1] a) P. M. Druce, B. M. Kingston, M. F. Lappert, T. R. Spalding, R. C. Srivastava, *J. Chem. Soc. A* **1969**, 2106–2110; b) T. Schaub, U. Radius, *Chem. Eur. J.* **2005**, *11*, 5024–5030; c) T. Schaub, M. Backes, U. Radius, *J. Am. Chem. Soc.* **2006**, *128*, 15964–15965; d) J. R. Herron, Z. T. Ball, *J. Am. Chem. Soc.* **2008**, *130*, 16486–16487.

[S2] N. Ignat'ev, P. Sartori, *J. Fluorine Chem.* **2000**, *103*, 57–61.

[S3] G. R. Fulmer, A. J. M. Miller, N. H. Sherden, H. E. Gottlieb, A. Nudelman, B. M. Stoltz, J. E. Bercaw, K. I. Goldberg, *Organometallics* **2010**, *29*, 2176–2179.

[S4] G. M. Sheldrick, *Acta Crystallogr. C.* **2015**, *71*, 3–8.

[S5] a) R. Ahlrichs, M. Bär, M. Häser, H. Horn, C. Kölmel, *Chem. Phys. Lett.* **1989**, *162*, 165–169; b) F. Furche, R. Ahlrichs, C. Hättig, W. Klopper, M. Sierka, F. Weigend, *WIREs Comput Mol Sci* **2014**, *4*, 91–100.

[S6] a) M. Häser, R. Ahlrichs, *J. Comput. Chem.* **1989**, *10*, 104–111; b) O. Treutler, R. Ahlrichs, *J. Chem. Phys.* **1995**, *102*, 346–354.

[S7] a) J. P. Perdew, *Phys. Rev. B* **1986**, *33*, 8822–8824; b) J. P. Perdew, *Phys. Rev. B* **1986**, *34*, 7406; c) A. D. Becke, *Phys. Rev. A* **1988**, *38*, 3098–3100.

[S8] a) J. P. Perdew, K. Burke, M. Ernzerhof, *Phys. Rev. Lett.* **1996**, *77*, 3865–3868; b) J. P. Perdew, M. Ernzerhof, K. Burke, *J. Chem. Phys.* **1996**, *105*, 9982–9985; c) J. P. Perdew, K. Burke, M. Ernzerhof, *Phys. Rev. Lett.* **1997**, *78*, 1396; d) C. Adamo, V. Barone, *J. Chem. Phys.* **1999**, *110*, 6158–6170; e) M. Ernzerhof, G. E. Scuseria, *J. Chem. Phys.* **1999**, *110*, 5029–5036; f) J. Tao, J. P. Perdew, V. N. Staroverov, G. E. Scuseria, *Phys. Rev. Lett.* **2003**, *91*, 146401; g) J. P. Perdew, J. Tao, V. N. Staroverov, G. E. Scuseria, *J. Chem. Phys.* **2004**, *120*, 6898–6911.

[S9] a) A. Schäfer, H. Horn, R. Ahlrichs, *J. Chem. Phys.* **1992**, *97*, 2571–2577; b) A. Schäfer, C. Huber, R. Ahlrichs, *J. Chem. Phys.* **1994**, *100*, 5829–5835; c) K. Eichkorn, O. Treutler, H. Öhm, M. Häser, R. Ahlrichs, *Chem. Phys. Lett.* **1995**, *242*, 652–660; d) F. Weigend, R. Ahlrichs, *Phys. Chem. Chem. Phys.* **2005**, *7*, 3297–3305; e) F. Weigend, *Phys. Chem. Chem. Phys.* **2006**, *8*, 1057–1065.

[S10] P. Deglmann, K. May, F. Furche, R. Ahlrichs, *Chem. Phys. Lett.* **2004**, *384*, 103–107.

[S11] K. O. Christe, D. A. Dixon, D. McLemore, W. W. Wilson, J. A. Sheehy, J. A. Boatz, *J. Fluorine Chem.* **2000**, *101*, 151–153.

[S12] I. Krossing, I. Raabe, *Chem. Eur. J.* **2004**, *10*, 5017–5030.

[S13] A. Klamt, G. Schüürmann, *J. Chem. Soc., Perkin Trans. 2* **1993**, 799– 805.
